# Environment heterogeneity creates fast amplifiers of natural selection in graph-structured populations

**DOI:** 10.1038/s41467-026-72784-z

**Published:** 2026-05-08

**Authors:** Cecilia Fruet, Arthur Alexandre, Alia Abbara, Claude Loverdo, Anne-Florence Bitbol

**Affiliations:** 1 Institute of Bioengineering, School of Life Sciences, École Polytechnique Fédérale de Lausanne (EPFL), Lausanne, Switzerland; 2 SIB Swiss Institute of Bioinformatics, Lausanne, Switzerland; 3 Laboratoire Jean Perrin (LJP), Sorbonne Université, CNRS, Paris, France; 4 Institut de Biologie Paris-Seine (IBPS), Sorbonne Université, CNRS, Inserm, Paris, France

**Keywords:** Evolutionary theory, Population genetics

## Abstract

Complex spatial structure, with partially isolated subpopulations, and environment heterogeneity, such as gradients in nutrients, oxygen, and drugs, both shape the evolution of natural populations. We investigate the impact of environment heterogeneity on mutant fixation in spatially structured populations with demes on the nodes of a graph. When migrations between demes are frequent, we find that environment heterogeneity can amplify natural selection and simultaneously accelerate mutant fixation and extinction, thereby fostering the quick fixation of beneficial mutants. We demonstrate this effect in the star graph, and more strongly in the line graph. We show that amplification requires mutants to have a stronger fitness advantage in demes with stronger migration outflow, and that this condition allows amplification in more general graphs. As a baseline, we consider circulation graphs, where migration inflow and outflow are equal in each deme. In this case, environment heterogeneity has no impact to first order, but increases the fixation probability of beneficial mutants to second order. Finally, when migrations between demes are rare, we show that environment heterogeneity can also foster amplification of selection, by allowing demes with sufficient mutant advantage to become refugia for mutants.

## Abstract (web summary)

Complex spatial structure and environment heterogeneity shape the evolution of natural populations. This study shows that environment heterogeneity can foster the quick fixation of beneficial mutants in populations structured into demes on a graph.

## Introduction

Natural microbial populations often possess complex spatial structures, with partially isolated subpopulations, e.g., in soil-associated or host-associated microbiota^1–5^. In addition, the environment in which natural microbial populations live is generally heterogeneous, featuring spatial variability in nutrient availability, temperature, pH, and concentrations of drugs or toxins. For instance, in soils, nutrient levels vary with depth, proximity to plant roots, and localized microbial metabolic activity^6,7^. This variability creates microhabitats that shape microbial community composition and function^8^. Aquatic environments are heterogeneous too^9^, influenced by factors such as oxygen gradients^10^. The human body also constitutes a spatially structured and heterogeneous environment for its microbiota^11^. In particular, the gut features gradients of oxygen, pH, bile salts, and antibiotic drugs when they are taken. This environment heterogeneity gives rise to heterogeneous selection pressures on microorganisms, leading to complex ecological and evolutionary dynamics^2,12,13^. To understand how natural microbial populations evolve, it is thus important to investigate the joint impact of spatial structure and environmental heterogeneity on population genetics.

The vast majority of theoretical studies investigating the evolution of spatially structured populations assume that the environment is homogeneous. They show interesting impacts of spatial structure on evolution, in particular on the fundamental process of mutant fixation, whereby a mutant type takes over the population. Considering well-mixed subpopulations, known as demes or patches, connected by migrations^14,15^, it was shown that the probability that a mutant fixes (i.e., takes over) in the population is not impacted by spatial structure, if migrations are sufficiently symmetric^16,17^, and demes do not get extinct^18^. Models on graphs where each node comprises one individual have allowed to consider more complex spatial structures^19^. In this framework, known as evolutionary graph theory, it was shown that specific graphs, under specific update rules defining the dynamics, can amplify or suppress natural selection^19–23^. Amplifiers of selection increase the fixation probability of a beneficial mutant compared to a well-mixed population with the same size, and decrease the one of deleterious mutants. Meanwhile, suppressors of selection decrease the fixation probability of beneficial mutants and increase that of deleterious ones. Amplifiers of natural selection have been the subject of sustained attention^19–25^, given their potential to enhance adaptation, which might be useful, e.g., in directed evolution. However, they generally slow down mutant fixation^26–29^, which limits their impact. Evolutionary graph theory models have been generalized by placing well-mixed demes with fixed size on graph nodes, also using specific update rules^30–34^. Recently, more general models, which do not assume strictly fixed deme sizes or update rules, were proposed by some of us^35,36^. Under rare migrations, these models generalize the findings of evolutionary graph theory, and show that whether a graph amplifies or suppresses natural selection strongly depends on the asymmetry of migration between demes^35^. Moreover, under frequent asymmetric migrations, suppression of selection was found to be pervasive and associated with an acceleration of mutant fixation or extinction^36^.

How does environmental heterogeneity across demes impact mutant fixation in spatially structured populations? In population genetics, environmental heterogeneity has been studied in continuous spatially extended populations, but in the context of species range limits and local adaptation rather than mutant fixation in the whole population^37–43^. Importantly, these studies typically consider antagonistic effects of selection across space, while here, our main focus is on mutants that are beneficial throughout the structure, with the strength of their beneficial effect varying across demes. In evolutionary graph theory models with one individual per node, graphs with nodes falling into two environment types have been considered^44–49^, mainly focusing on the optimal repartition of these two types of nodes to foster mutant fixation^46,47,49^ and on their impact on evolutionary timescales^44,48^. Here, we consider a more general model with demes on nodes of the graph, where mutant fitness advantage can be different in each deme. We investigate how environment heterogeneity affects mutant fixation in deme-structured populations on graphs, comparing heterogeneous and homogeneous environments.

We generalize the spatially structured population model introduced in ref. ^36^ to heterogeneous environments, modeled via a deme-dependent mutant fitness advantage. We first focus on the frequent migration regime. In circulation graphs, where migration inflow and outflow are equal in each deme, we find that environment heterogeneity has no impact to first order, but increases mutant fixation probability to second order. This simplest category of graphs serves as a baseline for the rest of our study. In the star and in the line, we show that environment heterogeneity substantially impacts mutant fixation probability, and that it can lead to amplification of selection, together with acceleration of mutant fixation and extinction. This stands in contrast with the usual tradeoff between amplification and slower dynamics. We determine the conditions for amplification of selection in the star and in the line analytically, using a branching process approach. We show that a key ingredient for amplification is that mutants should be strongly favored in demes with strong migration outflow. We generalize these findings to fully connected graphs with one special deme that differs from others both by mutant advantage and by migration outflow. Furthermore, we show that amplification can exist in all connected graphs with five nodes that are not circulations, with strong migration outflow from demes where mutants are advantaged. Finally, we turn to the rare migration regime and show that environment heterogeneity can turn suppressors of selection into amplifiers in this regime too, but for a different reason: demes with sufficient mutant advantage become refugia for mutants.

## Results

### Including environment heterogeneity in deme-structured populations on graphs

**Fig. 1:**
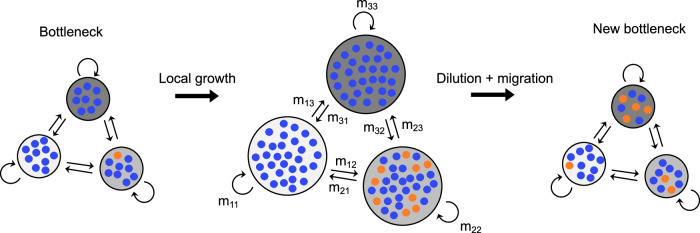
Serial dilution model for spatially structured populations with environmental heterogeneities. Schematic of an elementary step of the serial dilution model. Starting from a bottleneck, each deme undergoes a deterministic local growth step followed by a dilution and migration step, leading to a new bottleneck. Blue markers represent wild-type individuals and orange markers represent mutants. Gray backgrounds with different darkness represent different environments.

The impact of spatial structure on mutant fixation has traditionally been studied assuming homogeneous environments, where the fitness difference between mutants and wild-types is the same in the whole structured population^14–19,30–36,50–58^. Here, we use our model of deme-structured population on graphs^36^ and generalize it to include heterogeneous environments: each deme, located on a node of the graph, may have a different environment that can modulate the mutant fitness advantage. We denote the baseline mutant fitness advantage by *s*, and the local modulation in deme *i* by the prefactor *δ*_*i*_, so that the mutant fitness advantage in that deme is *sδ*_*i*_. In our serial dilution model, illustrated in Fig. 1, each deme starts at bottleneck size *K*, grows exponentially for a fixed time *t*, and then undergoes a dilution and migration step, returning to the original bottleneck size *K*^36^. These steps are then iterated. This description generalizes over structured Wright-Fisher models^59,60^ and allows explicit modeling of experiments with serial transfers^61,62^. The fitness advantage of mutants matters during the growth phase and results in an increase in the fraction of mutants in demes where mutants were present initially. This variation of mutant fraction only depends on *st*, which we thus refer to as the baseline effective fitness advantage (see “Methods”). After a growth phase, migrations take place with probability *m*_*ij*_ from deme *i* to deme *j* during each migration-dilution step, modeled using binomial samplings (see “Methods”).

Throughout, we focus on the fixation of a single mutant appearing uniformly at random in the structure, reflecting biologically realistic scenarios such as DNA replication errors or mutations due to external stress affecting all cells. We mainly address the frequent migration regime^36^, which is the most relevant experimentally^62–66^. In the final section, we turn to the rare migration regime, where mutant fixation or extinction in a deme occurs much faster than migration^35,51^.

Before investigating the role of environment heterogeneity, we briefly recall how spatial structure impacts mutant fixation in deme-structured populations on graphs with homogeneous environments. We showed in ref. ^36^ that circulation graphs, where each node has the same total incoming and outgoing migration flow, do not impact fixation probability. This extends Maruyama’s theorem^16,17^ and the circulation theorem of evolutionary graph theory models with one individual per node of a graph^19^. Furthermore, we showed that all other graphs suppress natural selection, and we found that this is associated with an acceleration of mutant fixation or extinction^36^. These results were obtained under frequent migrations using the branching process approximation.

### Environment heterogeneity in circulation graphs: a baseline

We first consider circulation graphs, which do not impact fixation probability under homogeneous environments. For frequent migrations, using a branching process approximation under the assumptions that *s* > 0, *K* ≫ 1 and 1/*K* ≪ *st* ≪ 1, the fixation probability of a mutant appearing uniformly at random can be expressed as

1 $$\rho=ast-\frac{b}{2}{(st)}^{2}\,,$$

to second order in the baseline effective fitness advantage *st* (see Supplementary Section 1.2). This expansion holds for any connected graph, but the coefficients *a* and *b* depend, a priori, on the graph and on the environment heterogeneity.

For circulation graphs, we find that *a* = 2〈*δ*〉 (see Supplementary Section 2.1.1). Hence, to first order in *st*, the fixation probability *ρ* = 2〈*δ*〉*st* only depends on the mean effective fitness advantage 〈*δ*〉*st* of the mutant across the structure, and is not affected by environment heterogeneity. We also find that the fixation probability does not depend on where the mutant starts. Furthermore, it coincides with the fixation probability in a well-mixed population with the same mean effective fitness advantage, matching Haldane’s classical result^67^. Thus, to first order in *st*, the circulation theorem extends to heterogeneous environments: in circulation graphs, neither spatial structure nor environment heterogeneity impacts mutant fixation probability.

By contrast, the second-order coefficient −*b* in the expansion of the fixation probability in Eq. (1) depends on environment heterogeneity even in circulation graphs (see Supplementary Section 2.1.3). For the clique (fully connected graph), corresponding to Wright’s island model^14,68^, our second-order calculation shows that environment heterogeneity slightly increases the fixation probability of beneficial mutants. For a specific cycle, corresponding to the circular stepping-stone model^15,16,69^, we obtain similar, though more complex, results (see Supplementary Section 2.1.3). These findings demonstrate that, beyond first order, environment heterogeneity has non-trivial theoretical effects even in circulation graphs, leading to a breakdown of the circulation theorem to second order. However, this effect remains quantitatively small, as it scales as (*st*)^2^. This motivates turning to non-circulation graphs to investigate whether environment heterogeneity has stronger effects there. Importantly, circulation graphs, and in particular the clique, will serve as a baseline independent of environment heterogeneity to first order in *st*.

### Environment heterogeneity strongly impacts mutant fixation probability in a star

**Fig. 2:**
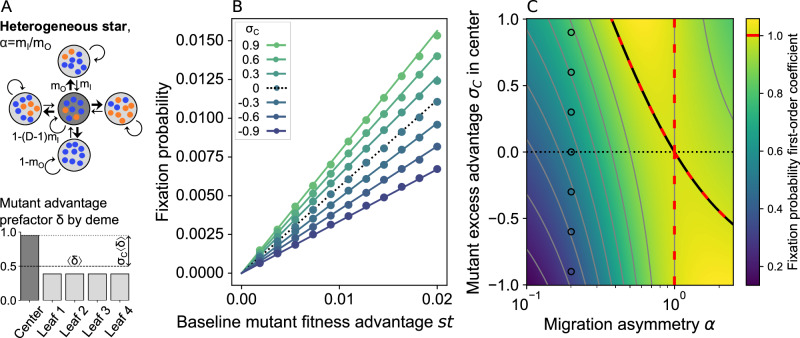
Impact of environmental heterogeneity on mutant fixation in the star. **A** Schematic of the heterogeneous star, with migration asymmetry *α* = *m*_*I*_/*m*_*O*_, and deme-dependent mutant advantage prefactor *δ* shown in the histogram below. The relative mutant fitness excess in the center is defined as *σ*_*C*_ = (*δ*_*C*_ − 〈*δ*〉)/〈*δ*〉. **B** Mutant fixation probability versus the baseline mutant fitness advantage *st*, for different relative mutant fitness excess *σ*_*C*_ in the center, with migration asymmetry *α* = 0.2. Markers: stochastic simulation results; lines: analytical predictions (Eq. S52). **C** Heatmap of the first-order coefficient in the expansion of the mutant fixation probability in the baseline effective mutant fitness advantage *st* (denoted by *a* in Eq. (1)), versus the migration asymmetry *α* and the relative mutant fitness excess *σ*_*C*_ in the center of the star. Red dashed lines: numerically-determined boundaries of the region where the star amplifies selection; solid black line: analytical prediction for this boundary, given by *f*_star_(*α*, *D*) in Eq. S69. Horizontal dotted line: homogeneous environment case (*σ*_*C*_ = 0). Markers: parameter values considered in (**B**). Parameter values: in (**B**, **C**), *D* = 5; *K* = 1000; 〈*δ*〉 = 0.5. In (**B**): *m*_*O*_ = 0.6; *α* = 0.2; each marker comes from 5 × 10^5^ stochastic simulation realizations.

We next consider the star graph, which comprises a central node connected to *D* − 1 leaf nodes by migrations (see Fig. 2A). The star has been the focus of many studies on the impact of spatial structure on mutant fixation under homogeneous environments. In evolutionary graph theory models with one individual per node, the star either amplifies or suppresses natural selection depending on update rules^19–22^. With demes on each node, in the rare migration regime, whether it amplifies or suppresses natural selection depends on migration asymmetry^35^. By contrast, for more frequent migrations, it can only suppress selection^36^.

How does environmental heterogeneity impact mutant fixation in the star with a deme on each node? To address this question, we compare a star with heterogeneous environments (see Fig. 2A) to a star with a homogeneous environment, where the mean mutant fitness advantage is the same, i.e., where the mutant fitness advantage is 〈*δ*〉*s* in each deme. In the branching process approach, we show that, to first order in *st*, the mutant fixation probability in the heterogeneous star only depends on selection through the mean mutant fitness effect, via 〈*δ*〉, and through the relative fitness excess in the center, defined as *σ*_*C*_ = (*δ*_*C*_ − 〈*δ*〉)/〈*δ*〉 (see Supplementary Section 2, Eq. S52). Fixation probability is also impacted by migration asymmetry *α* = *m*_*I*_/*m*_*O*_, where *m*_*I*_ is the probability of incoming migrations to the center from a leaf, while *m*_*O*_ is the probability of outgoing migrations from the center to a leaf. When there is more migration outflow from the center than inflow to the center (*α* < 1), we find that environment heterogeneity increases mutant fixation probability if the center features a larger mutant fitness advantage than the leaves (*σ*_*C*_ > 0). Conversely, if there is more inflow to the center than outflow (*α* > 1), heterogeneity increases mutant fixation probability if the center features a smaller mutant fitness advantage than the leaves (*σ*_*C*_ < 0), see Supplementary Section 3.1.2. This suggests that having a stronger mutant advantage in locations with more migration outflow enhances mutant fixation. Finally, if *α* = 1, to first order in *st*, the environment has no impact on fixation probability. This is consistent with our results above, as in this specific case, the star is a circulation graph.

Figure 2B shows the impact of environment heterogeneity, via *σ*_*C*_, on the mutant fixation probability in the star. We observe that the farther *σ*_*C*_ is from 0, the more the fixation probability deviates from the baseline of the homogeneous star. We observe that environment heterogeneity yields an increase in the fixation probability when *σ*_*C*_ > 0, and a decrease in it when *σ*_*C*_ < 0. As *α* < 1 in this figure, this is fully consistent with our analytical predictions. In fact, for each value of *σ*_*C*_ considered, Fig. 2B shows excellent agreement between our stochastic simulations and our analytical predictions.

Figure 2C further illustrates how migration asymmetry *α* and environmental heterogeneity, via *σ*_*C*_, impact the first-order coefficient *a* in Eq. (1) in the expansion in *st* of the mutant fixation probability in the star. This heatmap highlights the strong impact of both migration asymmetry and environmental heterogeneity on fixation probability in the star. Notably, environmental heterogeneity can enhance fixation probability in a star beyond that of an equivalent well-mixed population or circulation with the same average mutant fitness advantage 〈*δ*〉*s*. This reference fixation probability is 2〈*δ*〉*st* to first order, as shown above. Hence, a heterogeneous star can amplify natural selection. This effect is further illustrated in Supplementary Fig. 5. We find an analytical condition on *σ*_*C*_ for amplification to occur, see Supplementary Section 3.1.3. Figure 2C shows that it matches the numerical finding for the boundary of the area where the fixation probability in the heterogeneous star exceeds that of an equivalent well-mixed population. This result is particularly striking because the star with frequent asymmetric migrations is known to suppress natural selection in a homogeneous environment^36^. Even with rare migrations, no amplification is possible in the homogeneous star with *α* < 1^35^, while here, amplification exists for *α* < 1, for sufficiently large *σ*_*C*_. Therefore, our results demonstrate that the impact of environmental heterogeneity is strong enough to counteract this suppression and lead to amplification of selection.

### The heterogeneous line can substantially amplify selection and accelerate mutant fixation

**Fig. 3:**
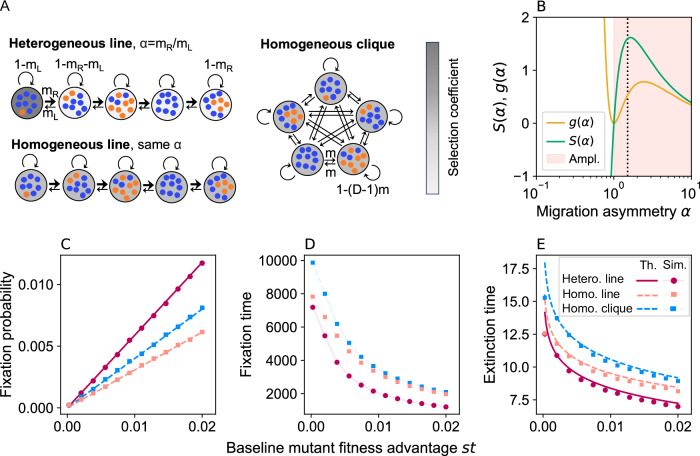
Amplification of selection and accelerated dynamics in the heterogeneous line. **A** Schematics of the structures considered: heterogeneous line with step environmental profile and *α* = 1.5; homogeneous line with same *α* and 〈*δ*〉; homogeneous clique with same 〈*δ*〉. **B** Condition for amplification of natural selection in the heterogeneous line shown in the top schematic. The functions *S* and *g*, defined respectively in Eqs. (2) and (3), are plotted versus migration asymmetry *α*. Pink-shaded region: range of *α* where *S*(*α*) > *g*(*α*), i.e., where the heterogeneous line amplifies natural selection. Dotted line: migration asymmetry (*α* = 1.5) chosen in (**C**–**E**). **C** Mutant fixation probability versus baseline mutant fitness advantage *st*, in the different spatial structures shown in (**A**). Predictions from the branching process theory ("Th.''), specifically Eq. S62, and results from stochastic simulations ("Sim.''), are shown. **D** Mutant fixation time, in number of bottlenecks, in the different structures considered, versus baseline mutant fitness advantage *st*. **E** Same as in (**D**), but for mutant extinction time. Theoretical predictions are from Eq. S13. Parameter values for all structures: *D* = 5, *K* = 1000; for all lines: *α* = 1.5, *m*_*L*_ = 0.3; for the clique: *m* = 0.15; for the line with step profile: *δ* = 1 in the leftmost deme, *δ* = 0 in other ones. Each marker comes from 5 × 10^5^ stochastic simulation realizations.

Another important spatial structure is the line of demes, or linear stepping-stone model^15,16,69^, see schematics in Fig. 3A. How does the line impact mutant fixation probability? In Supplementary Section 2, we derive the fixation probability of a mutant in a line of demes with frequent migrations in the branching process regime, both in the homogeneous and in the heterogeneous case, see Eq. S62. Note that the line was not addressed earlier in this regime, e.g., not in ref. ^36^. In the homogeneous case, as the star and all other graphs^36^, the line suppresses natural selection as soon as it has some migration asymmetry, i.e., *α* ≠ 1, where *α* = *m*_*R*_/*m*_*L*_ is the ratio of migration probabilities to the right and to the left. This is explicitly shown in Supplementary Section 3.2.1 and illustrated in Supplementary Fig. 6. Note that for *α* = 1, the line is a circulation.

How does environmental heterogeneity impact mutant fixation in the line? To address this question, we compare a line with an environment that can differ in each node *i*, through the mutant fitness advantage *δ*_*i*_*s*, to a homogeneous line with the same mean mutant fitness advantage 〈*δ*〉*s* (see Fig. 3A). Let us denote by *σ*_*i*_ = (*δ*_*i*_−〈*δ*〉)/〈*δ*〉 the relative mutant fitness excess in deme *i*. The fixation probability in the line with heterogeneous fitness is higher than in the homogeneous line if and only if:

2 $$S(\alpha )\equiv \mathop{\sum }_{i=1}^{D}{\sigma }_{i}{\alpha }^{-i} > 0,$$

see Supplementary Section 3.2.2. This entails that, in a heterogeneous line with monotonically decreasing mutant fitness advantage from left to right, the mutant fixation probability is higher than in the homogeneous line for *α* > 1, see Supplementary Section 3.2.2. The opposite holds for *α* < 1, and there is no impact of heterogeneity for *α* = 1 (when the line becomes a circulation), to first order in the baseline effective mutant fitness advantage *st*. Therefore, in the line, mutant spread and fixation are facilitated when the demes conferring the highest selective advantage to mutants are upstream of the overall migration flow.

We have shown that environment heterogeneity can increase fixation probability in the line. Can this result in amplification of selection? To address this question, we consider the first-order expansion in *st* of the fixation probability, and we compare the line to a well-mixed population with the same average mutant fitness advantage, i.e., the same 〈*δ*〉, or equivalently, to a clique with the same 〈*δ*〉 (see Fig. 3A). Comparing the fixation probabilities to first order, we show that a heterogeneous line has a higher fixation probability than a clique if and only if:

3 $$S(\alpha ) > g(\alpha )\equiv \frac{(D-1)\alpha -(D+1)+(D+1){\alpha }^{1-D}-(D-1){\alpha }^{-D}}{{\alpha }^{2}-1},$$

see Supplementary Section 3.2.3.

Figure 3B shows *S* and *g* as a function of *α* for the heterogeneous line shown in Fig. 3A, which has *D* = 5 demes and features a step profile of mutant fitness advantages, with *δ*_*i*_ = 1 for *i* = 1 and *δ*_*i*_ = 0 for *i* > 1. For such a decreasing profile of *δ*_*i*_, amplification of selection cannot exist for *α* < 1 (see Supplementary Section 3.2.3). We observe in Fig. 3B that amplification of selection exists when *α* > 1 (Eq. (3) is satisfied). Furthermore, for this particular environmental profile, *S*(*α*) > *g*(*α*) in asymptotic behavior for large *α*, see Supplementary Section 3.2.3. Note that for other environmental profiles, we observed that *g* and *S* cross for an α^*^ > 1, and that amplification exists in a finite region from *α* = 1 to *α* = α^*^.

Figure 3C shows the mutant fixation probability in the above-described heterogeneous line with a step environmental profile for *α* = 1.5, and evidences a strong amplification of selection compared to a homogeneous clique with the same average fitness. This stands in contrast to the homogeneous line with the same *α* and 〈*δ*〉, which suppresses selection, see Fig. 3C, and with a heterogeneous line with an opposite environmental profile but the same *α*, which further suppresses selection, see Supplementary Fig. 7. Moreover, Fig. 3D, E shows that the average mutant fixation and extinction times are both faster in our heterogeneous line than in the homogeneous clique with the same 〈*δ*〉. Hence, the heterogeneous line is able to both amplify selection and accelerate fixation at the same time. Importantly, this differs from the usual behavior of amplifiers in evolutionary graph theory, which slow down fixation^26–28^. This joint amplification and acceleration is observed for diverse environmental profiles, as illustrated in Supplementary Fig. 8. The results in Supplementary Fig. 8 suggest that larger environmental contrast between demes leads to more amplification of selection and to more acceleration of mutant fixation and extinction. Note that the same effect of joint amplification and acceleration, albeit less strong, exists for the heterogeneous star, see Supplementary Section 3.1.3 and Supplementary Fig. 5.

### Locally increasing mutant fitness advantage and migration outflow yield amplification of selection and acceleration of fixation

In the star and the line, we showed that environment heterogeneity can amplify natural selection and accelerate fixation when mutants have a stronger fitness advantage upstream of the overall migration flow. Do these findings hold beyond the star and the line, when migration asymmetries arise solely from differences in migration probabilities, rather than from the graph connection pattern? To address this question, we consider a graph where all nodes are connected by migrations, but where one special deme (called deme number 1) differs from all others both by migration outflow and by environment. The prefactor of mutant fitness advantage is *δ*_1_ in the special deme and *δ*_2_ in other demes.

**Fig. 4:**
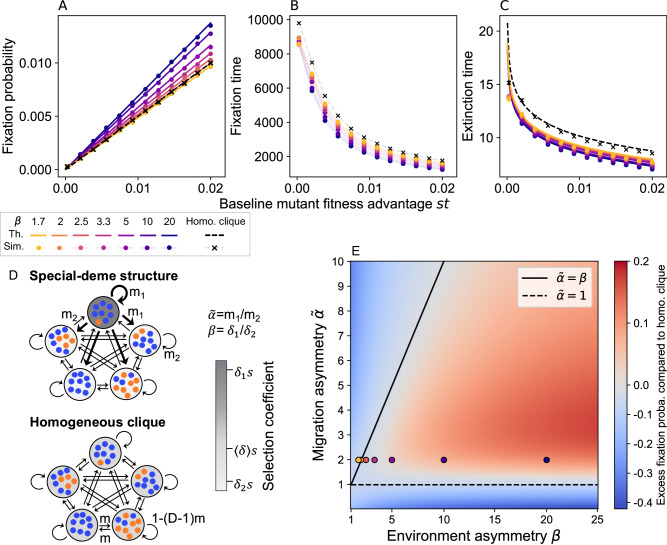
Amplification of selection and accelerated dynamics in structures with a special deme in terms of mutant advantage and migration outflow. **A** Mutant fixation probability versus baseline mutant fitness advantage *st* for fully-connected population structures with one special deme, characterized by stronger migration outflow ($\widetilde{\alpha }={m}_{1}/{m}_{2}=2$) and greater mutant advantage than others (*β* = *δ*_1_/*δ*_2_ > 1), see schematics in (**D**). Results for a homogeneous clique with the same 〈*δ*〉 are shown for reference. **B** Time to mutant fixation, in bottlenecks, versus baseline mutant fitness advantage *st*, for the same structures as in (**A**). **C** Same as in (**B**), but for extinction time. “Th.” indicates branching process theory (Eq. S90 for **A**, Eq. S13 for **C**), “Sim.” stochastic simulations (**A**–**C**). **D** The structures considered in this figure: a fully-connected structure with a special deme and a homogeneous clique. **E** The excess fixation probability in a structure with one special deme, compared to a homogeneous clique with the same 〈*δ*〉, is shown versus the migration asymmetry $\widetilde{\alpha }$ and the environment asymmetry *β*, in the case where migration probabilities are drawn from Dirichlet distributions (no strong symmetry in migration probabilities, generalizing over the structure considered in (**A**–**D**), see Supplementary Section 4.2). The plotted values represent the difference between the first-order coefficient in *st* of the fixation probability (denoted by *a* in Eq. (1), computed numerically following Supplementary Section 1.3, and averaged over many sets of migration probabilities), and the value for the homogeneous clique with the same 〈*δ*〉, which is 2〈*δ*〉. Solid and dashed black lines mark theoretical boundaries for amplification in the strongly symmetric structure considered in (**A**–**D**) (see Eq. (4) and main text): the solid line corresponds to $\widetilde{\alpha }=\beta$ and the dashed line to $\widetilde{\alpha }=1$. Markers show the $(\widetilde{\alpha },\beta )$ values considered in (**A**–**C**). Parameter values throughout: *D* = 5, 〈*δ*〉 = 0.25; for (**A**–**C**): *K* = 1000, $\widetilde{\alpha }=2$ and *m*_1_ = 0.33 for the special-deme structure, *m* = 0.15 for the homogeneous clique. Each value in the heatmap of (**E**) is obtained by averaging over 5 × 10^3^ structures, whose migration probabilities are drawn each time from Dirichlet distributions independently.

We first address the highly symmetric case in which all outgoing migration probabilities from the special deme are equal to *m*_1_, while all other migration probabilities are equal to *m*_2_, and $\widetilde{\alpha }={m}_{1}/{m}_{2}$ denotes migration asymmetry (Fig. 4A–D). When $\widetilde{\alpha } > 1$, meaning that the special deme has a stronger migration outflow than others, the spatial structure considered amplifies natural selection if and only if:

4 $$\frac{{\delta }_{1}}{{\delta }_{2}}\equiv \beta > \widetilde{\alpha },$$

see Supplementary Section 4.1. Hence, when the special deme sends out more individuals than others, selection is amplified if the mutant advantage in that deme is sufficiently stronger than elsewhere. More precisely, this environment contrast needs to be larger than the migration contrast. In the opposite case where $\widetilde{\alpha } < 1$, amplification occurs for $\beta < \widetilde{\alpha }$, see Supplementary Section 4.1. Hence, for amplification to exist in this structure, mutant advantage needs to be stronger upstream of the overall migration flow, and moreover, the environment needs to be more contrasted than the migration flows. Figure 4A shows that our prediction in Eq. (4) is satisfied: with $\widetilde{\alpha } > 1$, amplification is obtained for $\beta > \widetilde{\alpha }$, results coincide with those in the clique for $\beta=\widetilde{\alpha }$, and suppression occurs for $\beta < \widetilde{\alpha }$. Moreover, Fig. 4B–C shows that amplification of selection is accompanied by acceleration of fixation and extinction. This is consistent with our findings for the star and the line, and suggests that locally increasing mutant fitness advantage and migration outflow constitutes a general mechanism for amplification of selection and acceleration.

Do these findings depend on the strong symmetry in migration probabilities assumed above? To address this question, we consider a structure with a special deme, as before, but where for each deme, the migration probabilities incoming to that deme from all other ones are drawn from the same Dirichlet distribution. The parameters of this distribution are chosen such that the special (first) deme has a stronger expected migration outflow than others, see Supplementary Section 4.2, but with a substantial dispersion of migration probabilities, see Supplementary Fig. 9. Figure 4E shows that, in this more general structure, substantial amplification of selection exists in the parameter region where $\beta > \widetilde{\alpha } > 1$, consistent with the conditions derived for the highly-symmetric case above. This indicates that the mechanism yielding amplification is robust to heterogeneity in migration intensities within a fully connected graph.

### Amplification of selection extends to deleterious mutants and large graphs

So far, we focused on beneficial mutants (*s* > 0), allowing analytical insight using the branching process formalism. By definition, amplification of selection means that beneficial mutants have a higher fixation probability than in a well-mixed population or clique, while deleterious ones have a lower one^19^. To test whether amplification extends to deleterious mutants in our graph-structured populations with heterogeneous environments, we perform simulations in the star, the line, and the highly-symmetric structure with one special deme, under conditions where overall migration flows from demes with the strongest mutant fitness advantage to those with weaker advantage, and where we previously observed amplification for *s* > 0. We find that in all cases, amplification extends to deleterious mutants: their fixation probabilities are smaller than in a homogeneous clique with the same average environment 〈*δ*〉, see Supplementary Fig. 10.

Having confirmed that amplification extends to deleterious mutants, we next ask whether it persists in larger graphs. So far, we mainly considered relatively small graphs with five nodes. We now vary the number *D* of demes while keeping 〈*δ*〉 constant, in the same three highly-symmetric structures, using analytical expressions for the first-order coefficient in *st* of the mutant fixation probability derived from the branching process approach (denoted by *a* in Eq. (1)). In the star where all leaves have the same environment, and in the structure with one special deme, we hold the environment contrast between demes constant in addition to 〈*δ*〉. Under these conditions, we find that amplification can persist for all *D*, although its intensity decreases when *D* increases, see Supplementary Fig. 11. We further derive a condition on migration asymmetry and on contrast for amplification in the large-*D* limit in these structures, see Supplementary Sections 6.1 and 6.2. In contrast, in the line where all demes have the same environment, except the most upstream deme, amplification vanishes and suppression occurs beyond moderate *D* when 〈*δ*〉 and contrast are held constant, see Supplementary Fig. 12A. However, alternative conventions that hold 〈*δ*〉 but not contrast constant can produce amplification that increases with *D* in the line (Supplementary Fig. 12B; see Supplementary Section 6.3 for analytical conditions). These results demonstrate that amplification of selection can exist in large graphs with heterogeneous environments, when overall migration flows from demes with the strongest mutant fitness advantage to those with a weaker advantage. They also indicate that specific conditions on migration and environment are required to maintain significant amplification in large graphs.

### Amplification exists across graph structures with strong migration outflow from demes where mutants are advantaged

**Fig. 5:**
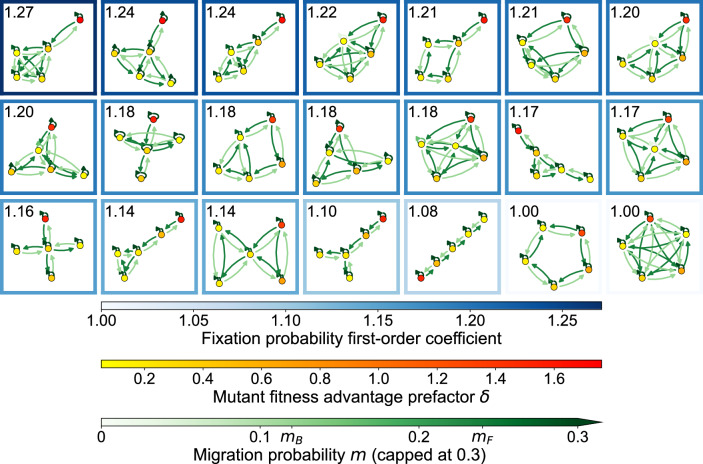
Amplification of selection across five-node graphs with strong migration outflow from demes where mutants are advantaged. Each panel shows a connected five-node graph, ordered by decreasing first-order coefficient in the expansion of the fixation probability in *st* (denoted by *a* in Eq. (1)). These coefficients are indicated in the top left corner of each panel, and shown through the panel frame’s color. They were calculated numerically as described in Supplementary Section 1.3. Migration probabilities and environment heterogeneity follow convention 1 defined in Supplementary Section 7, ensuring that overall migration flows from demes whith the strongest mutant fitness advantage to those with weaker advantage. For all graphs, 〈*δ*〉 = 0.5. Hence, circulations have a first-order coefficient 2〈*δ*〉 = 1, and coefficients larger than one indicate amplification of selection.

Does the amplification mechanism we identified persist in generic graph structures, beyond strongly symmetric or structurally simple graphs? To address this question, we now consider all connected graphs with five demes^21,70,71^, shown in Fig. 5. In all of them, we set migration probabilities and environment heterogeneity according to a convention that ensures that the overall migration flow is directed from the demes where mutants have the strongest fitness advantage to those where this advantage is smaller (see Supplementary Section 7). Figure 5 reports the value of the first-order coefficient in the expansion of the fixation probability in *st* in each structure (denoted by *a* in Eq. (1)), sorted from the highest to the lowest first-order coefficient. Amplification of selection exists in all graphs considered, except the cycle and the clique, which are both circulations. We further observe that amplification of selection is stronger when the deme where mutants are most advantaged has a small degree, while other nodes are strongly connected together. These results show that the amplification mechanism identified above extends from specific analytically tractable structures to diverse structures, indicating that amplification of selection can generically exist when overall migration flows from demes with the strongest mutant fitness advantage to those with a weaker advantage. Nevertheless, other conventions often lead to suppression of selection (see Supplementary Section 7 and Supplementary Fig. 14), suggesting that suppression remains the most generic effect of spatial structure under frequent migrations in the branching process regime, as for homogeneous environments^36^. Specific situations, such as the overall migration flow going in the same direction as the environment gradient, are required to reverse this trend.

### Heterogeneous environments can induce amplification of selection in the rare migration regime

So far, we considered frequent migrations and focused on the branching process regime, assuming that *K* ≫ 1 and 1/*K* ≪ *st* ≪ 1. In this regime, we showed that environment heterogeneity can turn suppressors of selection into amplifiers. Can this also occur in the rare-migration regime, where the timescale of migrations is much slower than mutant fixation or extinction within a deme? To address this question, let us return to the star graph and to the line graph. Their mutant fixation probabilities were determined analytically for homogeneous environments in the rare migration regime, respectively, in refs. ^35,72^. A key point under rare migrations is that a mutant that fixes always first takes over its deme of origin, before spreading to other demes, allowing a coarse-grained Markov chain description^35,51^. Here, we generalize these calculations to heterogeneous environments. For the star, we consider the case where the center has a different environment from the leaves, associated with the mutant advantage prefactor *δ*_*C*_, but the leaves all have the same environment, associated with *δ*_*L*_, see Supplementary Section 8.1. For the line, we perform a general calculation where all demes can have different environments, with a mutant advantage prefactor *δ*_*i*_ in deme *i*, see Supplementary Section 8.2.

**Fig. 6:**
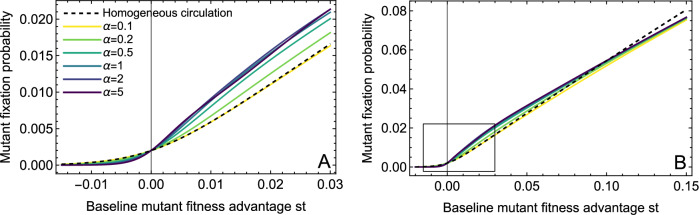
Amplification in the heterogeneous star with rare migrations. We show the mutant fixation probability, starting from one single mutant placed uniformly at random at a bottleneck (Eq. S118 using Eq. S113), in a heterogeneous star with various migration asymmetries *α*, and in a homogeneous circulation with the same mean mutant fitness advantage, i.e., with the same 〈*δ*〉. All fixation probabilities are plotted versus the baseline mutant fitness advantage *st*. **A**, **B** show different ranges of *st*. Parameter values: *D* = 5, *K* = 100. For the heterogeneous star, *δ*_*C*_ = 1 and *δ*_*L*_ = 0.1.

Figure 6 shows that the heterogeneous star in the rare migration regime can amplify natural selection, compared to a homogeneous circulation with the same mean mutant fitness, in a broad range of migration asymmetries *α* = *m*_*I*_/*m*_*O*_. Strikingly, this includes cases where *α* < 1, for which the star suppresses natural selection under rare migrations^35^. Hence, environment heterogeneity can turn suppressors of selection into amplifiers in the rare migration regime too. In Fig. 6, we observe amplification of selection for weakly deleterious and weakly beneficial mutants (recall that for deleterious mutants, amplification corresponds to having a smaller fixation probability than in the well-mixed or circulation case). The results of Fig. 6 are obtained with *δ*_*C*_ substantially larger than *δ*_*L*_, i.e., when beneficial (resp. deleterious) mutants are substantially more advantaged (resp. disadvantaged) in the center than in leaves. In this case, consider *st* such that 1/*K* ≪ 2*δ*_*C*_*st* ≪ 1 but 2*δ*_*L*_*st* ≲ 1/*K*. Then, if a mutant starting in the center fixes there, it should ultimately take over the whole population. Indeed, wild-type individuals migrating to the center from other leaves cannot take over again in the center, due to their substantial fitness disadvantage (their fixation probability is exponentially suppressed). The existence of a special “safe” deme for mutants sets the star apart from the homogeneous circulation with the same 〈*δ*〉, and causes amplification in this regime (see Supplementary Section 8.1 for further details, including an explanation of the suppression of selection observed for larger *st*). Recall that in the homogeneous star with *α* > 1, amplification of selection is observed under rare migrations^35^, but only when *st* ≲ 1/*K*^36^. In fact, in the rare migration regime, amplification effects are associated with the finite size of demes and only exist in that regime. Our new findings are consistent with this and shed light on how environmental heterogeneity can induce amplification in the rare migration regime.

In the case of the line, which is a suppressor of selection in the homogeneous case^72^ (see also refs. ^73,74^), we also find that environment heterogeneity can yield amplification of selection. This is shown in Supplementary Fig. 19, where we assume that only the leftmost deme has *δ*_*L*_ while the other ones have *δ*_*R*_ substantially smaller. A small amplification occurs in the regime where 1/*K* ≪ 2*δ*_*L*_*st* ≪ 1 but 2*δ*_*R*_*st* ≲ 1/*K*, see Supplementary Section 8.2. As in the star, the mutant benefits from the existence of a “safe” (leftmost) deme, where it cannot be re-invaded by wild-types. However, its location is less ideal than in the star, leading to a smaller effect. This illustrates the generality of the amplification mechanism by environment heterogeneity in the rare migration regime: it relies on refugia where mutants are safe from re-invasion, and is specific to finite-sized demes and to a range of small fitness differences between mutant and wild-type individuals.

## Discussion

In this work, we investigated how environment heterogeneity impacts mutant fixation in spatially structured populations with demes on nodes of a graph. We first focused on the frequent migration regime. We proved that the circulation theorem remains valid to first order in heterogeneous environments. However, to second order, environment heterogeneity increases the fixation probability of beneficial mutants in circulations. In non-circulation graphs, we demonstrated that environment heterogeneity can lead to amplification of selection, together with acceleration of mutant fixation and extinction. This result is striking for two reasons: first, without environment heterogeneity, no amplification is possible under frequent migrations^36^. Second, amplifiers of selection generally slow down fixation^26–29,70^. We found that this simultaneous amplification of selection and acceleration occurs in the star graph, and even more strongly in the line graph. We identified a key condition for amplification: mutants must be more favored in demes with stronger migration outflow. Qualitatively, individuals in a deme with strong outflow contribute more to the next generation. Thus, a fitness increase in this deme helps mutants more than in other demes, contributing to the amplification of selection. Under this condition, amplification can occur for deleterious as well as beneficial mutants and persists in large graphs. It also exists in more general graphs, including fully connected graphs with one special deme and all connected graphs with five nodes that are not circulations. Nevertheless, the most generic effect of spatial structure on mutant fixation remains suppression of selection, as in homogeneous environments^36^, since specific conditions on migration and environment are required to obtain amplification. Finally, in the rare migration regime, where mutant fixation or extinction in a deme is faster than migrations, environment heterogeneity can turn suppressors of selection into amplifiers.

While environment heterogeneity can foster amplification of selection under both frequent and rare migrations, the mechanisms differ between these two cases, as does the regime of mutant fitness advantage where amplification is observed. For frequent migrations, our branching process analysis focuses on the early and highly stochastic dynamics of a mutant lineage, long before it reaches a size comparable to that of a deme. In this regime, regions with both stronger mutant fitness advantage and larger migration outflow allow mutants to more successfully avoid extinction. Meanwhile, for rare migrations, fixation must occur within a deme before mutants can spread further. If the mutant fitness advantage is much larger than 1/*K* in a deme, this deme essentially cannot be re-invaded by wild-type individuals if the mutant type fixes there, since wild-type fixation is extremely unlikely. Such demes with sufficient mutant advantage become refugia for mutants, compared to other demes where mutant fitness advantage is smaller, and wild-types can take over again, thereby enabling amplification of selection. This effect is associated with the possibility that wild-types take over again in a deme, and thus requires small fitness advantages and finite deme sizes. This stands in contrast with the frequent-migration amplification effect.

Our results were obtained by extending to heterogeneous environments a general model of spatially structured populations on graphs, which does not rely on update rules^36^, and is close to experimental protocols of serial transfer^62–66,75,76^. Compared to previous studies of heterogeneous environments in evolutionary graph theory^44–49^, which generally focus on two environments and their repartition across nodes, we addressed generic heterogeneity across demes. We obtained results both in the frequent migration branching process regime and in the rare migration regime. While the latter can in some cases be mapped to evolutionary graph theory models with one individual per node^35^, the frequent migration regime is fundamentally different, as states where several demes comprise both mutants and wild-type individuals become central to the mutant’s fate. In population genetics, environmental heterogeneity in spatially extended populations has also been studied in the context of species range limits. In these works, when a mutation is beneficial in one region but deleterious in another, gene flow from the region where the mutation is deleterious can hinder local adaptation in the one where it is beneficial, an effect known as swamping^37–43^. These studies typically consider continuous spatial structures and antagonistic effects of selection across space^38,40,41^ or quantitative traits whose optimum varies spatially^39,42^, and focus on range expansion rather than mutant fixation or extinction. Despite these important differences, it is interesting to note that in swamping, migration from regions where a mutant is disadvantaged to regions where it is advantaged hampers adaptation, echoing our finding that the opposite migration flow (from more favorable to less favorable regions) can amplify natural selection.

Our analysis is relevant to experimental microbial populations, particularly in the frequent migration regime^61,62^, whereas the rare migration regime leads to very long fixation times. Moreover, our results have direct implications for natural microbial populations in spatially structured habitats, such as the gut. In the line graph, we showed that a decreasing mutant fitness along the main flow direction can foster amplification of selection. The gut can be considered as a one-dimensional system to a first approximation, and features a directional hydrodynamic flow together with a fitness gradient due to nutrient availability^77–79^. These ingredients have been shown to impact mutant fixation^80,81^. Our results for a discrete line of demes suggest additional effects for mutants with spatially-dependent advantages in the gut, in particular promoting and accelerating their fixation.

Our results, obtained for spatially structured populations with (almost) fixed sizes, also resonate with known ones for expanding populations. In particular, the presence of antibiotic concentration gradients can foster resistance evolution in expanding populations^82–85^. In these cases, expanding populations move toward zones where the mutant fitness advantage is larger. It would be interesting to generalize our model to include initially empty demes where the population can grow. Other promising directions would be to consider larger and more complex graphs^86^, including scale-free^87^ and small-world^88^ networks, and to include time-varying environments in demes^89,90^. Moreover, while our focus has been on the fate of a single mutation, it would be very interesting to investigate the impact of heterogeneous spatially structured populations on fitness landscape exploration^72,91,92^, longer-term evolution^93,94^, as well as on population diversity under frequent mutations^95–97^.

While here we focused on mutants whose fitness depends on the deme, the framework could also be extended to study social interactions, where fitness also depends on interactions between individuals within a deme. An intriguing perspective would be to investigate the impact of environment heterogeneity on social evolution in spatially structured populations, for instance, in the context of evolutionary games and the evolution of cooperation^98–106^. It would also be interesting to generalize such a study to group interactions^107,108^ and higher-order networks^109^, and to address cases where the environment or the graph coevolves with population composition^107,108^.

## Methods

### Serial dilution model on a graph with a heterogeneous environment

We model a spatially structured population as a set of well-mixed demes, each sitting on one node of a connected graph with *D* nodes. Migration probabilities *m*_*ij*_ are defined between any pair of demes (*i*, *j*) ∈ {1,..., *D*}^2^, including *i* = *j*, corresponding to individuals that stay in the same deme. They differ by their fitnesses, which are respectively *f*_*W*_ = 1 for wild-types (taken as reference) and *f*_*M*_ = 1 + *s* for mutants. Thus, *s* is the relative fitness advantage of mutants compared to wild-types. The population is composed of wild-types of fitness *f*_*W*_ = 1 (taken as reference) and mutants, whose fitness depends on the environment, and is denoted by *f*_*M*,*i*_ = 1 + *δ*_*i*_*s* in deme *i*. Here, *s* is the baseline mutant fitness advantage, and *δ*_*i*_ is an environment-dependent prefactor, which is nonnegative and of order unity if it is nonzero.

We generalize the model introduced in ref. ^36^ to heterogeneous environments. The dynamics of the population, modeling serial passage with migrations, is composed of alternations of phases of growth and migration-dilution, see Fig. 1.

Starting at a bottleneck, each deme separately undergoes deterministic exponential growth for a fixed time *t*. Fitnesses are understood as exponential growth rates. Therefore, the growth step is impacted by environment heterogeneity via the mutant fitness advantage *sδ*_*i*_, which depends on the deme *i*. Denoting by *x*_*i*_ the initial fraction of mutants in deme *i*, the fraction of mutants after growth in deme *i* is:

5 $${x}_{i}^{{\prime} }=\frac{{x}_{i}{e}^{{\delta }_{i}st}}{1+{x}_{i}({e}^{{\delta }_{i}st}-1)}\,.$$

Note that the impact of the baseline selective advantage of mutants is encoded by the product *st*. We thus call *st* the (effective) baseline fitness advantage.

After a growth phase, a dilution (regulation) and migration phase takes place. The number of mutants going from deme *i* to deme *j* is sampled from a binomial distribution with ${N}_{i}^{{\prime} }$ trials, with ${N}_{i}^{{\prime} }$ the size of the deme after growth, and a probability of success ${m}_{ij}{x}_{i}^{{\prime} }K/{N}_{i}^{{\prime} }$. Similarly for wild types, but with a probability of success ${m}_{ij}(1-{x}_{i}^{{\prime} })K/{N}_{i}^{{\prime} }$. Each deme *j* thus receives on average $K{\sum }_{i=1}^{D}\,{m}_{ij}$ individuals, leading to a new bottleneck state. We assume that for all *j*, ${\sum }_{i=1}^{D}{m}_{ij}=1$, so that all demes have the same average bottleneck size *K*. For each pair of demes (*i*, *j*), this phase corresponds to sampling approximately *Km*_*ij*_ bacteria from the total ${N}_{i}^{{\prime} }$ bacteria present in deme *i*, which contribute to the new bottleneck state of deme *j*.

Growth and dilution-migration phases are iterated. Importantly, this serial dilution model with migrations is very close to a structured Wright-Fisher model^59,60^. In fact, we showed in ref. ^36^ that for *s* ≪ 1, in the branching process approximation, results from structured Wright-Fisher models can be recovered from those of the serial dilution model by taking growth time *t* = 1. As a consequence, all our results here hold for structured Wright-Fisher models.

### Branching process description

The state of the population is described with a multi-type branching process, where each type corresponds to mutants in each deme^36^. The branching process description assumes that all mutant lineages are independent^110^. It holds when mutants are in small numbers and deme sizes are large, *K* ≫ 1. For mutants with substantial selective advantage *st* ≫ 1/*K*, extinction events happen when mutants are still rare^95,111^. Hence, the branching process approach holds when *K* ≫ 1 and *st* ≫ 1/*K*.

In the branching process regime, assuming that all nonzero migration probabilities are of order unity, the binomial distributions used to model a dilution-migration phase (see above) can be approximated by Poisson ones. Under these conditions, starting from one mutant in deme *i* at a bottleneck, the mutant extinction probability *p*_*i*_ satisfies:

6 $${p}_{i}=\exp \left[K{x}_{i}^{{\prime} }\mathop{\sum }_{j=1}^{D}{m}_{ij}({p}_{j}-1)\right],$$

for all *i*, where ${x}_{i}^{{\prime} }$ is given by Eq. (5) with *x*_*i*_ = 1/*K*. These equations generalize those of the homogeneous case^36^, as here the mutant fraction ${x}_{i}^{{\prime} }$ in deme *i* after growth involves *δ*_*i*_. To solve Eq. (6) for all *i*, we assume that the baseline mutant fitness advantage is positive but small, 0 < *st* ≪ 1, and perform a Taylor expansion, see Eq. (1) and Supplementary Section 1. The fixation probability starting from one mutant in deme *i* at a bottleneck is then *ρ*_*i*_ = 1−*p*_*i*_. We focus on the average fixation probability $\rho={\sum }_{i=1}^{D}{\rho }_{i}/D$ across the structure, assuming that mutants appear uniformly at random in the structure.

The average mutant extinction time can also be obtained in the branching process regime, see Supplementary Section 1.4.

### Rare migration regime

When the timescale of migrations is much slower than mutant fixation or extinction within a deme, one can consider that each deme is always fully wild type or fully mutant as far as migration events are concerned. This allows a coarse-grained Markov chain description^35,51^, which has allowed the determination of exact fixation probabilities in the homogeneous case^35,72^. Here, we extend this approach to the heterogeneous case, see Supplementary Section 8.1. Contrary to the branching process results, these results hold for all values of *st*, including for deleterious, neutral, and effectively neutral mutants. Besides, they connect to evolutionary graph theory models^35^. However, the rare migration regime is associated with slow dynamics. In contrast, the branching process calculations assume that migration probabilities are of order unity, so these two approaches cover entirely distinct regimes and provide complementary insights.

## Supplementary information

Supplementary Information

Transparent Peer Review file

## Data Availability

All the data relevant to this study are simulation data and are included in the paper or in the Supplementary Material. The code used to generate this simulation data, allowing us to reproduce our analyses, is available as specified below.

## Code availability

Our code is freely available at https://github.com/Bitbol-Lab/StructPop_Heterogeneity and archived on Zenodo^112^.

## References

[1] Fierer, N. Embracing the unknown: disentangling the complexities of the soil microbiome. *Nat. Rev. Microbiol.* **15**, 579–590 (2017). 28824177 10.1038/nrmicro.2017.87

[2] Donaldson, G. P., Lee, S. M. & Mazmanian, S. K. Gut biogeography of the bacterial microbiota. *Nat. Rev. Microbiol.* **14**, 20–32 (2016). 26499895 10.1038/nrmicro3552 PMC4837114

[3] Wu, F. et al. Modulation of microbial community dynamics by spatial partitioning. *Nat. Chem. Biol.* **18**, 394–402 (2022). 35145274 10.1038/s41589-021-00961-w PMC8967799

[4] Edwards, J., Johnson, C., Santos-Medellín, C. & Sundaresan, V. Structure, variation, and assembly of the root-associated microbiomes of rice. *Proc. Natl. Acad. Sci. USA* **112**, E911–E920 (2015). 25605935 10.1073/pnas.1414592112 PMC4345613

[5] Bickel, S. & Or, D. Soil bacterial diversity mediated by microscale aqueous-phase processes across biomes. *Nat. Commun.* **11**, 1–9 (2020). 31913270 10.1038/s41467-019-13966-w PMC6949233

[6] Jobbágy, E. G. & Jackson, R. B. The distribution of soil nutrients with depth: Global patterns and the imprint of plants. *Biogeochemistry* **53**, 51–77 (2001).

[7] Naylor, D., McClure, R. & Jansson, J. Trends in microbial community composition and function by soil depth. *Microorganism* **10**, 540 (2022). 10.3390/microorganisms10030540 PMC8954175 35336115

[8] Torsvik, V., Øvreås, L. & Thingstad, T.F. Prokaryotic diversity–magnitude, dynamics, and controlling factors. *Science* **296**, 1064–1066 (2002). 12004116 10.1126/science.1071698

[9] Stocker, R. Marine microbes see a sea of gradients. *Science* **338**, 628–633 (2012). 23118182 10.1126/science.1208929

[10] Fenchel, T. & Finlay, B. Oxygen and the spatial structure of microbial communities. *Biol. Rev. Camb. Philos. Soc.* **83**, 553–569 (2008). 18823390 10.1111/j.1469-185X.2008.00054.x

[11] Costello, E. K. et al. Bacterial community variation in human body habitats across space and time. *Science* **326**, 1694–1697 (2009). 19892944 10.1126/science.1177486 PMC3602444

[12] McCallum, G. & Tropini, C. The gut microbiota and its biogeography. *Nat. Rev. Microbiol.* **22**, 105–118 (2023). 37740073 10.1038/s41579-023-00969-0

[13] Verdon, N., Popescu, O., Titmuss, S. & Allen, R. J. Habitat fragmentation enhances microbial collective defence. *J. R. Soc. Interface* **22**, 20240611 (2025). 39933594 10.1098/rsif.2024.0611 PMC11813583

[14] Wright, S. Evolution in Mendelian populations. *Genetics* **16**, 97–159 (1931). 17246615 10.1093/genetics/16.2.97 PMC1201091

[15] Kimura, M. & Weiss, G. H. The stepping stone model of population structure and the decrease of genetic correlation with distance. *Genetics* **49**, 561–576 (1964). 17248204 10.1093/genetics/49.4.561 PMC1210594

[16] Maruyama, T. On the fixation probability of mutant genes in a subdivided population. *Genet. Res.* **15**, 221–225 (1970). 5480754 10.1017/s0016672300001543

[17] Maruyama, T. A simple proof that certain quantities are independent of the geographical structure of population. *Theor. Popul. Biol.* **5**, 148–154 (1974). 4825532 10.1016/0040-5809(74)90037-9

[18] Barton, N. The probability of fixation of a favoured allele in a subdivided population. *Genet. Res.* **62**, 149–157 (1993).

[19] Lieberman, E., Hauert, C. & Nowak, M. A. Evolutionary dynamics on graphs. *Nature* **433**, 312–315 (2005). 15662424 10.1038/nature03204

[20] Kaveh, K., Komarova, N. L. & Kohandel, M. The duality of spatial death-birth and birth-death processes and limitations of the isothermal theorem. *R. Soc. Open Sci.* **2**, 140465 (2015). 26064637 10.1098/rsos.140465 PMC4448870

[21] Hindersin, L. & Traulsen, A. Most undirected random graphs are amplifiers of selection for birth-death dynamics, but suppressors of selection for death-birth dynamics. *PLOS Comput. Biol.* **11**, 1–14 (2015). 10.1371/journal.pcbi.1004437 PMC4636432 26544962

[22] Pattni, K., Broom, M., Rychtář, J. & Silvers, L. J. Evolutionary graph theory revisited: When is an evolutionary process equivalent to the Moran process? *Proc. R. Soc. A Math. Phys. Eng. Sci.* **471**, 20150334 (2015).

[23] Tkadlec, J., Pavlogiannis, A., Chatterjee, K. & Nowak, M. A. Limits on amplifiers of natural selection under death-birth updating. *PLOS Comput. Biol.* **16**, e1007494 (2020). 31951609 10.1371/journal.pcbi.1007494 PMC6968837

[24] Adlam, B., Chatterjee, K. & Nowak, M. A. Amplifiers of selection. *Proc. R. Soc. A Math., Phys. Eng. Sci.* **471**, 20150114 (2015).

[25] Pavlogiannis, A., Tkadlec, J., Chatterjee, K. & Nowak, M. A. Amplification on undirected population structures: comets beat stars. *Sci. Rep.* **7**, 82 (2017). 28250441 10.1038/s41598-017-00107-w PMC5427850

[26] Frean, M., Rainey, P. B. & Traulsen, A. The effect of population structure on the rate of evolution. *Proc. R. Soc. B Biol. Sci.* **280**, 20130211 (2013). 10.1098/rspb.2013.0211 PMC3673044 23677339

[27] Hindersin, L., Möller, M., Traulsen, A. & Bauer, B. Exact numerical calculation of fixation probability and time on graphs. *Biosystems* **150**, 87–91 (2016). 27555086 10.1016/j.biosystems.2016.08.010

[28] Tkadlec, J., Pavlogiannis, A., Chatterjee, K. & Nowak, M. A. Population structure determines the tradeoff between fixation probability and fixation time. *Commun. Biol.* **2**, 138 (2019). 31044163 10.1038/s42003-019-0373-y PMC6478818

[29] Tkadlec, J., Pavlogiannis, A., Chatterjee, K. & Nowak, M. A. Fast and strong amplifiers of natural selection. *Nat. Commun.* **12**, 4009 (2021). 34188036 10.1038/s41467-021-24271-w PMC8242091

[30] Houchmandzadeh, B. & Vallade, M. The fixation probability of a beneficial mutation in a geographically structured population. *New J. Phys.* **13**, 073020 (2011).

[31] Houchmandzadeh, B. & Vallade, M. Exact results for fixation probability of bithermal evolutionary graphs. *Biosystems* **112**, 49–54 (2013). 23567507 10.1016/j.biosystems.2013.03.020

[32] Constable, G. W. & McKane, A. J. Population genetics on islands connected by an arbitrary network: an analytic approach. *J. Theor. Biol.* **358**, 149–165 (2014). 24882790 10.1016/j.jtbi.2014.05.033

[33] Yagoobi, S. & Traulsen, A. Fixation probabilities in network structured meta-populations. *Sci. Rep.* **11**, 17979 (2021). 34504152 10.1038/s41598-021-97187-6 PMC8429422

[34] Yagoobi, S., Sharma, N. & Traulsen, A. Categorizing update mechanisms for graph-structured metapopulations. *J. R. Soc. Interface* **20**, 20220769 (2023). 36919418 10.1098/rsif.2022.0769 PMC10015335

[35] Marrec, L., Lamberti, I. & Bitbol, A.-F. Toward a Universal Model for Spatially Structured Populations. *Phys. Rev. Lett.* **127**, 218102 (2021). 34860074 10.1103/PhysRevLett.127.218102

[36] Abbara, A. & Bitbol, A.-F. Frequent asymmetric migrations suppress natural selection in spatially structured populations. *PNAS Nexus* **2**, pgad392 (2023). 38024415 10.1093/pnasnexus/pgad392 PMC10667037

[37] Haldane, J.B.S. The relation between density regulation and natural selection. *Proc. R. Soc. Lond. B. Biol. Sci.* **145**, 306–308 (1956). 13359386 10.1098/rspb.1956.0039

[38] Slatkin, M. Gene flow and selection in a Cline. *Genetics* **75**, 733–756 (1973). 4778791 10.1093/genetics/75.4.733 PMC1213045

[39] Kirkpatrick, M. & Barton, N. H. Evolution of a species’ range. *Am. Nat.* **150**, 1–23 (1997). 18811273 10.1086/286054

[40] Lenormand, T. Gene flow and the limits to natural selection. *Trends Ecol. Evol.* **17**, 183–189 (2002).

[41] Kawecki, T. J. & Ebert, D. Conceptual issues in local adaptation. *Ecol. Lett.* **7**, 1225–1241 (2004).

[42] Polechová, J. & Barton, N. H. Limits to adaptation along environmental gradients. *Proc. Natl. Acad. Sci. USA* **112**, 6401–6406 (2015). 25941385 10.1073/pnas.1421515112 PMC4443383

[43] Kottler, E. J., Dickman, E. E., Sexton, J. P., Emery, N. C. & Franks, S. J. Draining the swamping hypothesis: little evidence that gene flow reduces fitness at range edges. *Trends Ecol. Evol.* **36**, 533–544 (2021). 33745756 10.1016/j.tree.2021.02.004

[44] Maciejewski, W. & Puleo, G. J. Environmental evolutionary graph theory. *J. Theor. Biol.* **360**, 117–128 (2014). 25016047 10.1016/j.jtbi.2014.06.040

[45] Kaveh, K., McAvoy, A. & Nowak, M. A. Environmental fitness heterogeneity in the Moran process. *R. Soc. Open Sci.* **6**, 181661 (2019). 30800394 10.1098/rsos.181661 PMC6366185

[46] Kaveh, K., McAvoy, A., Chatterjee, K. & Nowak, M. A. The Moran process on 2-chromatic graphs. *PLOS Comput. Biol.* **16**, 1–18 (2020). 10.1371/journal.pcbi.1008402 PMC7671562 33151935

[47] Brendborg, J., Karras, P., Pavlogiannis, A., Rasmussen, A.U. & Tkadlec, J. Fixation maximization in the positional Moran process. In *Proc. AAAI Conference on Artificial Intelligence* Vol. 36, 9304–9312 (AAAI Press, 2022).

[48] Svoboda, J., Tkadlec, J., Kaveh, K. & Chatterjee, K. Coexistence times in the Moran process with environmental heterogeneity. *Proc. R. Soc. A Math. Phys. Eng. Sci.* **479**, 20220685 (2023).

[49] Nemati, H., Kaveh, K. & Ejtehadi, M.R Counterintuitive properties of evolutionary measures: a stochastic process study in cyclic population structures with periodic environments. *J. Theor. Biol.* **564**, 111436 (2023). 36828246 10.1016/j.jtbi.2023.111436

[50] Nagylaki, T. The strong-migration limit in geographically structured populations. *J. Math. Biol.* **9**, 101–114 (1980). 7365330 10.1007/BF00275916

[51] Slatkin, M. Fixation probabilities and fixation times in a subdivided population. *Evolution* **35**, 477–488 (1981). 28563585 10.1111/j.1558-5646.1981.tb04911.x

[52] Whitlock, M. C. & Barton, N. H. The effective size of a subdivided population. *Genetics* **146**, 427–441 (1997). 9136031 10.1093/genetics/146.1.427 PMC1207958

[53] Nordborg, M. & Krone, S. M. Separation of time scales and convergence to the coalescent in structured populations. in *Modern Developments in Theoretical Population Genetics: The Legacy of Gustave Malécot* (eds Slatkin, M. & Veuille, M.) 194—232 (Oxford University Press, 2002).

[54] Whitlock, M. C. Fixation probability and time in subdivided populations. *Genetics* **164**, 767–779 (2003). 12807795 10.1093/genetics/164.2.767 PMC1462574

[55] Sjödin, P., Kaj, I., Krone, S., Lascoux, M. & Nordborg, M. On the meaning and existence of an effective population size. *Genetics* **169**, 1061–1070 (2005). 15489538 10.1534/genetics.104.026799 PMC1449138

[56] Hauert, C., Chen, Y.-T. & Imhof, L. A. Fixation times in deme structured, finite populations with rare migration. *J. Stat. Phys.* **156**, 739–759 (2014).

[57] Allen, B. et al. Evolutionary dynamics on any population structure. *Nature* **544**, 227–230 (2017). 28355181 10.1038/nature21723

[58] Kreger, J., Brown, D., Komarova, N. L., Wodarz, D. & Pritchard, J. The role of migration in mutant dynamics in fragmented populations. *J. Evol. Biol.* **36**, 444–460 (2023). 36514852 10.1111/jeb.14131 PMC10108075

[59] Lessard, S. An exact sampling formula for the Wright-Fisher model and a solution to a conjecture about the finite-island model. *Genetics* **177**, 1249–1254 (2007). 17660540 10.1534/genetics.107.077644 PMC2034630

[60] Burden, C. & Griffiths, R. Stationary distribution of a 2-island 2-allele Wright-Fisher diffusion model with slow mutation and migration rates. *Theor. Popul. Biol.* **124**, 70–80 (2018). 30308179 10.1016/j.tpb.2018.09.004

[61] Chakraborty, P.P., Nemzer, L. R. & Kassen, R. Experimental evidence that network topology can accelerate the spread of beneficial mutations. *Evol. Lett.* **7**, 447–456 (2023). 38045727 10.1093/evlett/qrad047 PMC10693003

[62] Abbara, A., Pagani, L., García-Pareja, C. & Bitbol, A.-F. Mutant fate in spatially structured populations on graphs: connecting models to experiments. *PLOS Comput. Biol.* **20**, e1012424 (2024). 39241045 10.1371/journal.pcbi.1012424 PMC11410244

[63] Kryazhimskiy, S., Rice, D. P. & Desai, M. M. Population subdivision and adaptation in asexual populations of Saccharomyces cerevisiae. *Evolution* **66**, 1931–1941 (2012). 22671557 10.1111/j.1558-5646.2011.01569.x

[64] Nahum, J. R. et al. A tortoise-hare pattern seen in adapting structured and unstructured populations suggests a rugged fitness landscape in bacteria. *Proc. Natl. Acad. Sci. USA* **112**, 7530–7535 (2015). 25964348 10.1073/pnas.1410631112 PMC4475941

[65] France, M. T. & Forney, L. J. The relationship between spatial structure and the maintenance of diversity in microbial populations. *Am. Nat.* **193**, 503–513 (2019). 30912968 10.1086/701799

[66] Chakraborty, P. P., Nemzer, L. R. & Kassen, R. Experimental evidence that network topology can accelerate the spread of beneficial mutations. *Evol. Lett.* **7**, 447–456 (2023). 38045727 10.1093/evlett/qrad047 PMC10693003

[67] Haldane, J. B. S. A mathematical theory of natural and artificial selection, Part V: selection and mutation. *Math. Proc. Camb. Philos. Soc.* **23**, 838–844 (1927).

[68] Crow, J. F. & Kimura, M. *An Introduction to Population Genetics Theory* (Blackburn, 2009).

[69] Maruyama, T. Stepping stone models of finite length. *Adv. Appl. Probab.* **2**, 229–258 (1970).

[70] Hindersin, L. & Traulsen, A. Counterintuitive properties of the fixation time in network-structured populations. *J. R. Soc. Interface* **11**, 20140606 (2014). 25142521 10.1098/rsif.2014.0606 PMC4233741

[71] Möller, M., Hindersin, L. & Traulsen, A. Exploring and mapping the universe of evolutionary graphs identifies structural properties affecting fixation probability and time. *Commun. Biol.* **2**, 137 (2019). 31044162 10.1038/s42003-019-0374-x PMC6478964

[72] Servajean, R., Alexandre, A. & Bitbol, A.-F. Impact of complex spatial population structure on early and long-term adaptation in rugged fitness landscapes. *Evolution* **79**, 935–950 (2025). 40037529 10.1093/evolut/qpaf025

[73] Broom, M. & Rychtář, J. An analysis of the fixation probability of a mutant on special classes of non-directed graphs. *Proc. R. Soc. A Math. Phys. Eng. Sci.* **464**, 2609–2627 (2008).

[74] Miller, J. W. A matrix equation approach to solving recurrence relations in two-dimensional random walks. *J. Appl. Probab.* **31**, 646–659 (1994).

[75] Wahl, L. M., Gerrish, P. J. & Saika-Voivod, I. Evaluating the impact of population bottlenecks in experimental evolution. *Genetics* **162**, 961–971 (2002). 12399403 10.1093/genetics/162.2.961 PMC1462272

[76] LeClair, J. S. & Wahl, L. M. The impact of population bottlenecks on microbial adaptation. *J. Stat. Phys.* **172**, 114–125 (2018).

[77] Cremer, J. et al. Effect of flow and peristaltic mixing on bacterial growth in a gut-like channel. *Proc. Natl. Acad. Sci. USA* **113**, 11414–11419 (2016). 27681630 10.1073/pnas.1601306113 PMC5068270

[78] Cremer, J., Arnoldini, M. & Hwa, T. Effect of water flow and chemical environment on microbiota growth and composition in the human colon. *Proc. Natl. Acad. Sci. USA* **114**, 6438–6443 (2017). 28588144 10.1073/pnas.1619598114 PMC5488924

[79] Salari, A. & Cremer, J. Diurnal variations in digestion and flow drive microbial dynamics in the gut. *PRX Life* **3**, 023012 (2025).

[80] Labavić, D., Loverdo, C. & Bitbol, A.-F. Hydrodynamic flow and concentration gradients in the gut enhance neutral bacterial diversity. *Proc. Natl. Acad. Sci. USA* **119**, e2108671119 (2022). 34969835 10.1073/pnas.2108671119 PMC8740595

[81] Ghosh, O. M. & Good, B. H. Emergent evolutionary forces in spatial models of luminal growth and their application to the human gut microbiota. *Proc. Natl. Acad. Sci. USA* **119**, e2114931119 (2022). 35787046 10.1073/pnas.2114931119 PMC9282425

[82] Zhang, Q. et al. Acceleration of emergence of bacterial antibiotic resistance in connected microenvironments. *Science* **333**, 1764–1767 (2011). 21940899 10.1126/science.1208747

[83] Greulich, P., Waclaw, B. & Allen, R. J. Mutational pathway determines whether drug gradients accelerate evolution of drug-resistant cells. *Phys. Rev. Lett.* **109**, 088101 (2012). 23002776 10.1103/PhysRevLett.109.088101

[84] Hermsen, R., Barrett Deris, J. & Hwa, T. On the rapidity of antibiotic resistance evolution facilitated by a concentration gradient. *Proc. Natl. Acad. Sci. USA* **109**, 10775–10780 (2012). 22711808 10.1073/pnas.1117716109 PMC3390829

[85] Baym, M. et al. Spatiotemporal microbial evolution on antibiotic landscapes. *Science* **353**, 1147–1151 (2016). 27609891 10.1126/science.aag0822 PMC5534434

[86] Kuo, Y.P., Nombela-Arrieta, C. & Carja, O. A theory of evolutionary dynamics on any complex population structure reveals stem cell niche architecture as a spatial suppressor of selection. *Nat. Commun.* **15**, 4666 (2024). 38821923 10.1038/s41467-024-48617-2 PMC11143212

[87] Barabási, A.-L. & Albert, R. Emergence of scaling in random networks. *Science* **286**, 509–512 (1999). 10521342 10.1126/science.286.5439.509

[88] Watts, D. J. & Strogatz, S. H. Collective dynamics of ‘small-world’ networks. *Nature* **393**, 440–442 (1998). 9623998 10.1038/30918

[89] Hernández-Navarro, L., Asker, M., Rucklidge, A. M. & Mobilia, M. Coupled environmental and demographic fluctuations shape the evolution of cooperative antimicrobial resistance. *J. R. Soc. Interface* **20**, 20230393 (2023). 37907094 10.1098/rsif.2023.0393 PMC10618063

[90] Hernández-Navarro, L., Asker, M. & Mobilia, M. Eco-evolutionary dynamics of cooperative antimicrobial resistance in a population of fluctuating volume and size. *J. Phys. A Math. Theor.* **57**, 265003 (2024).

[91] de Visser, J. A. G. M. & Krug, J. Empirical fitness landscapes and the predictability of evolution. *Nat. Rev. Genet.* **15**, 480–490 (2014). 24913663 10.1038/nrg3744

[92] Das, S. G., Mungan, M. & Krug, J. Epistasis-mediated compensatory evolution in a fitness landscape with adaptational tradeoffs. *Proc. Natl. Acad. Sci.* **122**, e2422520122 (2025). 40215274 10.1073/pnas.2422520122 PMC12012525

[93] Sharma, N. & Traulsen, A. Suppressors of fixation can increase average fitness beyond amplifiers of selection. *Proc. Natl. Acad. Sci. USA* **119**, e2205424119 (2022). 36067304 10.1073/pnas.2205424119 PMC9478682

[94] Sharma, N., Das, S. G., Krug, J. & Traulsen, A. Graph-structured populations elucidate the role of deleterious mutations in long-term evolution. *Nat. Commun.* **16**, 2355 (2025). 40064927 10.1038/s41467-025-57552-9 PMC11894086

[95] Desai, M. M. & Fisher, D. S. Beneficial mutation-selection balance and the effect of linkage on positive selection. *Genetics* **176**, 1759–1798 (2007). 17483432 10.1534/genetics.106.067678 PMC1931526

[96] Good, B. H., McDonald, M. J., Barrick, J. E., Lenski, R. E. & Desai, M. M. The dynamics of molecular evolution over 60,000 generations. *Nature* **551**, 45–50 (2017). 29045390 10.1038/nature24287 PMC5788700

[97] Blundell, J. R. et al. The dynamics of adaptive genetic diversity during the early stages of clonal evolution. *Nat. Ecol. Evol.* **3**, 293–301 (2019). 30598529 10.1038/s41559-018-0758-1 PMC6517070

[98] Turner, P. E. Prisoner’s dilemma in an RNA virus. *Nature* **398**, 441–443 (1999). 10201376 10.1038/18913

[99] Doebeli, M. & Hauert, C. Models of cooperation based on the prisoner’s dilemma and the snowdrift game. *Ecol. Lett.* **8**, 748–766 (2005).

[100] Ohtsuki, H., Hauert, C., Lieberman, E. & Nowak, M. A. A simple rule for the evolution of cooperation on graphs and social networks. *Nature* **441**, 502–505 (2006). 16724065 10.1038/nature04605 PMC2430087

[101] Ohtsuki, H. & Nowak, M. A. Evolutionary games on cycles. *Proc. R. Soc. B: Biol. Sci.* **273**, 2249–2256 (2006). 10.1098/rspb.2006.3576 PMC1635521 16901846

[102] Traulsen, A. & Hauert, C. Stochastic evolutionary game dynamics. *Rev. Nonlinear Dyn. Complex.* **2**, 25–61 (2009).

[103] Gokhale, C. S. & Traulsen, A. Evolutionary multiplayer games. *Dyn. Games Appl.* **4**, 468–488 (2014).

[104] Cremer, J. et al. Cooperation in microbial populations: theory and experimental model systems. *J. Mol. Biol.* **431**, 4599–4644 (2019). 31634468 10.1016/j.jmb.2019.09.023

[105] Moawad, A., Abbara, A. & Bitbol, A.-F. Evolution of cooperation in deme-structured populations on graphs. *Phys. Rev. E* **109**, 024307 (2024). 38491653 10.1103/PhysRevE.109.024307

[106] Ribière, D., Abbara, A. & Bitbol, A.-F. Promotion of cooperation in deme-structured populations with growth-merging dynamics. Preprint at *bioRxiv*10.64898/2026.01.25.701574 (2026).

[107] Perc, M. & Szolnoki, A. Coevolutionary games—a mini review. *Biosystems* **99**, 109–125 (2010). 19837129 10.1016/j.biosystems.2009.10.003

[108] Perc, M., Gómez-Gardeñes, J., Szolnoki, A., Floría, L. M. & Moreno, Y. Evolutionary dynamics of group interactions on structured populations: a review. *J. R. Soc. Interface* **10**, 20120997 (2013). 23303223 10.1098/rsif.2012.0997 PMC3565747

[109] Alvarez-Rodriguez, U. et al. Evolutionary dynamics of higher-order interactions in social networks. *Nat. Hum. Behav.* **5**, 586–595 (2021). 33398148 10.1038/s41562-020-01024-1

[110] Harris, T. E. *The Theory of Branching Processes* (Springer, 1963).

[111] Boenkost, F., Gonzalez-Casanova, A., Pokalyuk, C. & Wakolbinger, A. Haldane’s formula in Cannings models: the case of moderately strong selection. *J. Math. Biol.* **83**, 70 (2021). 34870765 10.1007/s00285-021-01698-9 PMC8648686

[112] Fruet, C., Alexandre, A., Abbara, A., Loverdo, C. & Bitbol, A.-F. Environment heterogeneity creates fast amplifiers of natural selection in graph-structured populations. https://github.com/Bitbol-Lab/StructPop_Heterogeneity; 10.5281/zenodo.19396437 (2026). 10.1038/s41467-026-72784-z PMC13369156 42103739

