# Supplementary material for: Environment heterogeneity creates fast amplifiers of natural selection in graph-structured populations
Source: Nat Commun. 2026 May 8;17:6228. doi: 10.1038/s41467-026-72784-z (PMC13369156; doi:10.1038/s41467-026-72784-z)
Supplement: Supplementary file 1 — Supplementary Information [file 41467_2026_72784_MOESM1_ESM.pdf]

Supplementary Information for "Environment heterogeneity creates fast amplifiers of natural selection in graph-structured populations"

Cecilia Fruet[1,2], Arthur Alexandre[1,2], Alia Abbara[1,2], Claude Loverdo[3,4], Anne-Florence Bitbol[1,2,*]

1 Institute of Bioengineering, School of Life Sciences, École Polytechnique Fédérale de Lausanne (EPFL), Lausanne, Switzerland
2 SIB Swiss Institute of Bioinformatics, Lausanne, Switzerland
3 Sorbonne Université, CNRS, Laboratoire Jean Perrin, LJP, Paris, France
4 Sorbonne Université, CNRS, Inserm, Institut de Biologie Paris-Seine, IBPS, Paris, France
* Corresponding author. Email: anne-florence.bitbol@epfl.ch

Contents

1 Branching process approach for frequent migrations **2**
1.1 Poisson approximation . . . 2
1.2 Expansion of the mutant fixation probability . . . 3
1.3 General determination of the mutant fixation probability to first order in *st* . . . 3
1.4 Average mutant extinction time . . . 4

2 Mutant fixation probability in analytically tractable spatial structures with heterogeneous environment **4**
2.1 Circulation graphs . . . 5
2.1.1 Circulation theorem to first order in *st* with heterogeneous environment . . . 6
2.1.2 Specific circulation graphs: clique and cycle with heterogeneous environment . . . 6
2.1.3 Beyond the circulation theorem: second order in *st* . . . 8
2.2 Star with heterogeneous environment . . . 12
2.3 Line with heterogeneous environment . . . 13

3 Comparison of fixation probabilities between structures **14**
3.1 Comparison of fixation probabilities in the star and in the clique . . . 14
3.1.1 Homogeneous star versus clique . . . 14
3.1.2 Homogeneous star versus heterogeneous star . . . 15
3.1.3 Heterogeneous star versus clique . . . 15
3.2 Comparison of fixation probabilities in the line and in the clique . . . 16
3.2.1 Homogeneous line versus clique . . . 17
3.2.2 Homogeneous line versus heterogeneous line . . . 17
3.2.3 Heterogeneous line versus clique . . . 18

4 Spatial structures with one special deme **21**
4.1 Highly symmetric structure with one special deme . . . 22
4.1.1 Fixation probability . . . 22
4.1.2 Condition for amplification of selection . . . 22
4.2 More general structures with one special deme . . . 23

5 Deleterious mutants **24**

6 Impact of the number of demes on the fixation probability **25**
6.1 Star . . . 25
6.2 Highly-symmetric structure with one special deme . . . 26
6.3 Line . . . 27

7 More general graph structures **29**

8 Rare migration regime **32**
8.1 Star with heterogeneous environment . . . 32
8.1.1 Mutant fixation probability . . . 32
8.1.2 Amplification of selection . . . 34
8.2 Line with heterogeneous environment . . . 34
8.2.1 Mutant fixation probability . . . 34
8.2.2 Amplification of selection . . . 42

1 Branching process approach for frequent migrations

1.1 Poisson approximation

For completeness, we briefly restate the stochastic process defined in the main text section "Methods", in order to set notations, and to introduce the Poisson approximation for the migration-dilution process. We then calculate mutant fixation probabilities in the branching process regime.

We consider spatially structured populations with D demes, each located on each node of a graph. We assume that there are two types of individuals, mutants and wild-types. Let us denote by s the baseline relative fitness advantage of mutants with respect to wild-types. Let us further model environment heterogeneity by introducing a deme-dependent prefactor δ_i in the mutant relative fitness advantage, such that in deme i, we have a relative fitness difference of $\delta_i s$ between the two types. In other words, taking wild-type fitness as reference ($f_W = 1$), we have $f_{M,i} = 1+\delta_i s$, where $f_{M,i}$ denotes mutant fitness in deme i, while f_W denotes wild-type fitness. We assume that δ_i is nonnegative, and of order unity if it is nonzero.

We start from the serial dilution model presented in Ref. [1], and we extend it to the heterogeneous case, where mutant fitness advantage is deme-dependent. In this model, demes undergo alternations of local deterministic exponential growth for time t, and of events of simultaneous dilution and migration leading to new bottlenecks, see Figure 1. We call m_{ij} the migration probability from deme i to deme j and assume $\sum_{i=1}^{D} m_{ij} = 1$ for all j.

We focus on the fate of a mutant that appears in deme i at a bottleneck, giving an initial mutant fraction $x_0^{(i)} = 1/K$ in that deme, where K is bottleneck size, and 0 in other demes. For this, we consider the first growth and migration-dilution events happening. The mutant fraction after exponential growth reads in deme i [1]:

$$x_0'^{(i)} = \frac{e^{\delta_i st}}{K + e^{\delta_i st} - 1} \equiv \frac{\lambda^{(i)}}{K}\,, \tag{S1}$$

where we introduced $\lambda^{(i)}$, which is a deme-specific growth factor. Then, we model the subsequent migration-dilution event through binomial sampling from each deme to each other demes. In particular, mutants are sampled from the grown deme i, of size N_i', to a destination deme j through the binomial law $\mathcal{B}\left(N_i', K m_{ij} x_0'^{(i)}/N_i'\right)$. Assuming $N_i' \gg 1$, $K m_{ij} x_0'^{(i)}/N_i' \ll 1$ and $\lambda_{ij} = K m_{ij} x_0'^{(i)} \sim 1$, we approximate the binomial law by a Poisson law with mean

$$\lambda_{ij} = K m_{ij} x_0'^{(i)} = \lambda^{(i)} m_{ij}\,. \tag{S2}$$

Compared to the homogeneous case considered in Ref. [1], the impact of heterogeneity is that the growth factor $\lambda^{(i)}$ depends on the deme i.

1.2 Expansion of the mutant fixation probability

Assuming $s > 0$, $K \gg 1$ and $1/K \ll st \ll 1$, and following a multi-type branching process description, as in Ref. [1], the probability p_i that mutants gets extinct, starting from one single mutant in deme i at the bottleneck, satisfies:

$$p_i = \exp\left[\lambda^{(i)} \sum_{j=1}^{D} m_{ij}(p_j - 1)\right] . \tag{S3}$$

Note that, while this equation regards the mutant extinction probability, the mutant fixation probability can then be obtained as $\rho_i = 1 - p_i$.

To solve the equations in Eq. S3, we perform a perturbative expansion in $st \ll 1$:

$$p_j = 1 - a_j st + \frac{b_j}{2}(st)^2 - \frac{c_j}{6}(st)^3 + o((st)^3) \qquad j = 1, \dots, D\,, \tag{S4}$$

where a_j, b_j and c_j are coefficients to be determined. Expanding Eq. S1 yields

$$\lambda^{(j)} = 1 + \delta_j st + \frac{\delta_j^2 (st)^2}{2} + o((st)^2) \qquad j = 1, \dots, D. \tag{S5}$$

Eq. S3 then yields, for $j = 1, \dots, D$:

$$\begin{aligned} p_j &= \exp\left[\left(1 + \delta_j st + \delta_j^2 \frac{(st)^2}{2} + o((st)^2)\right) \sum_{k=1}^{D} m_{jk} \left(-a_k st + \frac{b_k}{2}(st)^2 - \frac{c_k}{6}(st)^3 + o((st)^3)\right)\right] \\ &= \exp\left[-st \sum_{k=1}^{D} m_{jk} a_k + (st)^2 \sum_{k=1}^{D} m_{jk}\left(\frac{b_j}{2} - a_j\delta_j\right) + (st)^3 \sum_{k=1}^{D} m_{jk}\left(\frac{b_k}{2}\delta_j - a_k\frac{\delta_j^2}{2} - \frac{c_k}{6}\right) + o((st)^3)\right] . \end{aligned} \tag{S6}$$

This gives, to third order in $st \ll 1$:

$$\begin{aligned} p_j =& 1 - st \sum_k m_{jk} a_k + \frac{(st)^2}{2}\left[\left(\sum_{k=1}^{D} m_{jk} a_k\right)^2 + \sum_{k=1}^{D} m_{jk}\left(b_k - 2\delta_j a_k\right)\right] \\ &+ (st)^3 \left[-\frac{1}{6}\left(\sum_{k=1}^{D} m_{jk} a_j\right)^3 - \sum_{k=1}^{D} m_{jk} a_k \sum_{l=1}^{D} m_{jl}\left(\frac{b_l}{2} - a_l\delta_j\right) + \sum_{k=1}^{D} m_{jk}\left(\frac{b_k}{2}\delta_j - a_k\frac{\delta_j^2}{2} - \frac{c_k}{6}\right)\right] . \end{aligned} \tag{S7}$$

Eqs. S4 and S7 yield by identification, for $j = 1, \dots, D$:

$$\begin{cases} a_j = \sum_{k=1}^{D} m_{jk} a_k \,, \\ b_j = \sum_{k=1}^{D} m_{jk} b_k + a_j^2 - 2\delta_j a_j \,, \\ c_j = \sum_{k=1}^{D} m_{jk} c_k - 2a_j^3 + 3a_j b_j - 3\delta_j b_j + 3\delta_j a_j^2 - 3\delta_j^2 a_j . \end{cases} \tag{S8}$$

The first line in Eq. S8 is the same in a homogeneous environment [1], but the other ones differ, as they involve δ_j.

In the following sections, we use Eq. S8 to calculate the first-order coefficients a_i first numerically for any strongly connected graph in Section 1.3 and then analytically for different graphs with strong symmetries or simple structures in Section 2.

1.3 General determination of the mutant fixation probability to first order in st

Before performing explicit analytical calculations in symmetric structures (see Section 2), we present a general method that allows one to numerically obtain the first-order coefficient in st of the fixation probability in any strongly connected graph. A strongly connected graph is a graph where there is a path between each pair of vertices of the graph, in both directions.

Let us first consider the first line in Eq. S8, which can be rewritten in vector form as:

$$M\boldsymbol{a} = \boldsymbol{a}\,, \tag{S9}$$

with $\boldsymbol{a} = (a_1, \ldots, a_D)$. Thus, we are looking for an eigenvector of M for eigenvalue 1. As the graph is strongly connected, its migration matrix M is irreducible. Since M is an irreducible, non-negative matrix, and satisfies $\sum_i m_{ij} = 1$ for all j, the Perron–Frobenius theorem guarantees that 1 is an eigenvalue of M, and is simple.

To determine the norm of the corresponding eigenvector $\boldsymbol{a}$, we use the second line in Eq. S8. We isolate b_j, use $\sum_{k=1}^{D} m_{kj} = 1$, and sum over all possible j:

$$\begin{aligned}
b_j &= m_{jj} b_j + \sum_{k\neq j} m_{jk} b_k + a_j^2 - 2\delta_j a_j\,, \text{ i.e.} \\
b_j - \Big(1 - \sum_{k\neq j} m_{kj}\Big) b_j &= \sum_{k\neq j} m_{jk} b_k + a_j^2 - 2\delta_j a_j\,, \text{ thus} \\
\sum_j \sum_{k\neq j} m_{kj} b_j &= \sum_j \sum_{k\neq j} m_{jk} b_k + \sum_j a_j^2 - \sum_j 2\delta_j a_j\,, \text{ i.e.} \\
0 &= \sum_j a_j^2 - 2\sum_j \delta_j a_j\,.
\end{aligned} \tag{S10}$$

For given δ_j values, this equation provides a constraint on the a_i, which allows to determine the norm of $\boldsymbol{a}$. Indeed, considering an eigenvector $\boldsymbol{a}'$ of M with eigenvalue 1, the vector $\boldsymbol{a}$ we are looking for can be written as $\boldsymbol{a} = C\boldsymbol{a}'$, where C is a constant, which should satisfy:

$$C^2 \sum_j (a'_j)^2 = 2C \sum_j a'_j \delta_j\,. \tag{S11}$$

With $C \neq 0$, this finally yields $\boldsymbol{a} = C\boldsymbol{a}'$ with

$$C = \frac{2\sum_j a'_j \delta_j}{\sum_j (a'_j)^2}\,. \tag{S12}$$

1.4 Average mutant extinction time

The average extinction time $t_i^{(\mathrm{ex})}$ of the lineage of a single mutant introduced in deme i at a bottleneck can be calculated in the branching process approximation from the probability generating function [1, 2]. Specifically, in a multi-type branching process, the vector of extinction times, expressed in number of generations, $\boldsymbol{t}^{(\mathrm{ex})} = \left(t_1^{(\mathrm{ex})}, t_2^{(\mathrm{ex})}, \ldots, t_D^{(\mathrm{ex})}\right)$, is given by:

$$\boldsymbol{t}^{(\mathrm{ex})} = \boldsymbol{f}(0) + \sum_{n=1}^{\infty} n \left[\boldsymbol{f}^{(n)}(0) - \boldsymbol{f}^{(n-1)}(0)\right], \tag{S13}$$

with $\boldsymbol{f}$ the generating function, whose component i satisfies:

$$f_i : \boldsymbol{x} \mapsto \exp\left[\lambda^{(i)} \sum_j m_{ij}(x_j - 1)\right], \tag{S14}$$

with $\lambda^{(i)}$ given by Eq. S5.

2 Mutant fixation probability in analytically tractable spatial structures with heterogeneous environment

In this Section, we consider specific graphs with strong symmetries or simple structures, and we investigate the impact of environment heterogeneity across demes on the fixation probability of a

mutant appearing uniformly at random in one of the demes of the population. Using the branching process approach introduced above, we provide analytical expressions of the fixation probability to first order in st in the structures shown in Figure S1, and we push the expansion to second order in the clique and in a particular cycle. These two graphs are circulations, and we first analyze this category of graphs.

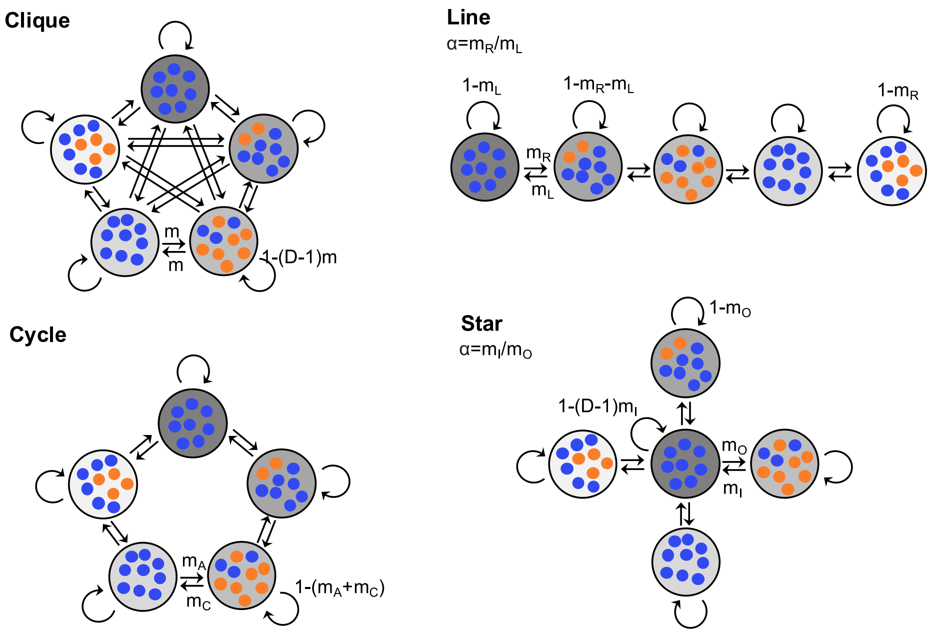

Figure S1: **Some spatial structures with environment heterogeneity considered in this study.** Schematics are shown for the clique with migration probability m, the line with migration probability to the right m_R and to the left m_L, the cycle with migration probability m_A in the anticlockwise direction and m_C in the clockwise direction, and the star, with incoming migration probability m_I from each leaf to the center, and outgoing migration probability m_O from the center to each leaf. Blue markers represent wild-type individuals and orange markers represent mutants. Gray backgrounds with different darkness represent different environments.

2.1 Circulation graphs

Circulations are graphs such that for each node i, $\sum_j m_{ji} = \sum_j m_{ij}$. In other words, for each deme i, the total incoming migration flow to deme i is equal to the total outgoing migration flow from deme i. Concrete examples are the clique and the cycle in Figure S1. With homogeneous environments, these structures, which possess strongly symmetric migrations, are known to exhibit the same mutant fixation probability as a well-mixed population with the same size, whatever the initial location of the mutant. A version of this result is known in population genetics models with demes on the nodes of highly-symmetric graphs [3, 4] as *Maruyama's theorem*, and it was further formalized in evolutionary graph theory models with one individual per node of a graph [5], as the *circulation theorem*. In Ref. [6], we showed that the circulation theorem extends to our model with demes in the rare migration regime, up to a finite-size effect correction. Then, in Ref. [1], we showed that it holds exactly under the branching process approximation, in the case of frequent migrations between demes. Thus, circulations are a class of graphs where spatial structure has (essentially) no effect on mutant fixation probability for homogeneous environments. In this Section, we investigate whether the circulation theorem still holds with environment heterogeneity. As in Section 1, we focus on the regime of frequent migrations and use the branching process approach.

2.1.1 Circulation theorem to first order in st with heterogeneous environment

For circulation graphs, using the Perron-Frobenius theorem and the first line of Eq. S8 (which is not affected by environment heterogeneity) allows us to show that all the a_i coefficients equal, using the approach presented in Ref. [1]. Let us briefly recall this proof for completeness. The the first line of Eq. S8 shows that the vector $(a_1, \dots, a_D)$ is an eigenvector of the matrix M of migration probabilities with eigenvalue 1. As the matrix M is non-negative and irreducible [1], the Perron-Frobenius theorem ensures that the largest real eigenvalue r of M is simple, positive, and satisfies:

$$\min_i \sum_j m_{ij} \leq r \leq \max_i \sum_j m_{ij} \,. \tag{S15}$$

As circulations satisfy $\sum_i m_{ij} = \sum_j m_{ij} = 1$, we have $r = 1$. Since $(1, \dots, 1)$ is an eigenvector of M associated to 1, it generates the eigenspace associated to the eigenvalue 1, and thus $(a_1, \dots, a_D)$ is proportional to $(1, \dots, 1)$, i.e. all the a_i coefficients equal. Let us call them a.

Moreover, the second line of Eq. S8 then provides $a = 2\langle\delta\rangle$ (see Eq. S10 in Section 1.3, written in the specific case where all a_i are equal). Hence, to first order in st, the probability of fixation of a mutant starting from any deme in a circulation is:

$$\rho = 2\langle\delta\rangle st \,, \tag{S16}$$

Hence, only the mean relative fitness advantage of the mutant across the structure matters for the mutant fixation probability in heterogeneous circulations. Furthermore, the result in Eq. S16 is the same as in a well-mixed population with the same $\langle\delta\rangle$ [7]. Thus, to first order in the branching process regime, the circulation theorem extends to structures with heterogeneous fitness differences across demes. Concretely, to first order, neither the spatial structure nor the environment heterogeneity within a circulation have any impact on fixation probability. All heterogeneous circulations have the same fixation probability as the corresponding homogeneous well-mixed population.

We show in Figure S2A our analytical prediction for the first-order fixation probabilities, given by Eq. S16, together with simulation results for four different circulations with heterogeneous environments: a clique, a cycle, a star with migration asymmetry $\alpha = m_I/m_O = 1$, and a line with migration asymmetry $\alpha = m_R/m_L = 1$ (see Figure S1). We also report results for the well-mixed population with mutant fitness advantage $\langle\delta\rangle s$. Our simulation results agree with our analytical prediction. We also show the mutant extinction time in Figure S2B, both from simulations and from a numerical calculation (see Section 1.4), for the four circulation graphs and the corresponding well-mixed population. We again observe good agreement between results from simulations and calculations.

2.1.2 Specific circulation graphs: clique and cycle with heterogeneous environment

In SI Sec. 2.1.1, we derived the fixation probability of a mutant in any circulation graph with heterogeneous environment to first order in st. The clique and the cycle graphs are circulations, for all values of m in the clique and of m_A and m_C in the cycle (see Figure S1). Note that this stands in contrast with the star and the line, which are circulations only when $\alpha = 1$ (see Figure S1). The clique and the cycle are thus fully covered by the general result in Eq. S16. Given their particularly high symmetry, more direct calculations of the first-order fixation probability can also be performed in the clique and the cycle. We provide them below for completeness.

Clique with heterogeneous environment. In the clique, $m_{ij} = m$ for all $i \neq j$. Hence, the first line of Eq. S8 becomes $a_j = m \sum_{k=1}^{D} a_k$ for all j. This implies that all a_j coefficients are equal, say to a, consistently with our general result derived above for circulations.

To obtain the value of a, we now use the second line of Eq. S8:

$$b_j m(D-1) = m \sum_{k \neq j} b_k + a^2 - 2a\delta_j \qquad j = 1, \dots, D \,. \tag{S17}$$

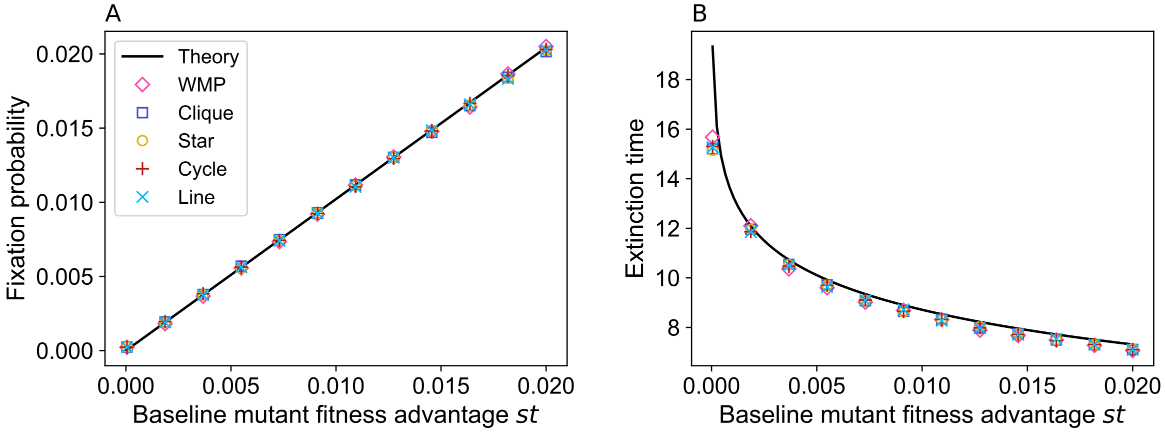

Figure S2: **Fixation probability and extinction time for structured populations on circulation graphs with environmental heterogeneity across demes.** Panel A: Fixation probability for four different graph structures: cycle, clique, line, and star (see Figure S1), under the condition $\sum_i m_{ij} = \sum_i m_{ji}$ for all nodes j, is shown as a function of the baseline mutant fitness advantage st. Panel B: Mutant extinction time, expressed in number of bottlenecks, as a function of the baseline mutant fitness advantage st, for the same structures. Solid lines represents results from the branching process theory (Eq. S16 in panel A, Eq. S13 in panel B), while markers represent results from stochastic simulations. The well-mixed population ("WMP") simulation result for the same $\langle\delta\rangle$ is included in both panels for reference. Parameter values: $D = 5$; $K = 1000$; δ_i values: $0.49, 0.32, 0.70, 0.41, 0.62$ (independently sampled from a uniform distribution on the interval $[0, 1]$), resulting in $\langle\delta\rangle = 0.51$; $m = m_I = m_O = 0.15$; $m_L = m_R = m_A = m_C = 0.3$; each marker comes from 5×10^5 simulation realizations.

Summing Eq. S17 over j yields:

$$\begin{aligned} m(D-1)\sum_{j=1}^{D} b_j &= m\sum_{j=1}^{D}\sum_{k\neq j} b_k + Da^2 - 2a\sum_{j=1}^{D}\delta_j \\ &= m(D-1)\sum_{j=1}^{D} b_j + Da^2 - 2a\sum_{j=1}^{D}\delta_j\,. \end{aligned} \tag{S18}$$

Thus, as $a \neq 0$, we obtain the first-order coefficient $a = 2\langle\delta\rangle$ for the clique, with $\langle\delta\rangle = \sum_{j=1}^{D}\delta_j/D$.

Therefore, the fixation probability ρ_i of a single mutant starting in deme i at a bottleneck in a clique reads to first order in st:

$$\rho_i = 1 - p_i = ast = 2\langle\delta\rangle st\,, \quad \text{for all } i\,, \tag{S19}$$

which coincides with our general result for circulations in Eq. S16.

Cycle with heterogeneous environment. Let us now consider the cycle, with migration probabilities to nearest neighbor demes that can differ in the clockwise and anticlockwise directions, and are denoted by m_C and m_A, respectively (see Figure S1).

Here, we use the fact that all a_i are equal to the same value a in a circulation, see the beginning of Section 2.1.1. The second line of Eq. S8 then reads:

$$b_j = \sum_{k=1}^{D} m_{jk}b_k + a^2 - 2\delta_j a = m_{jj}b_j + \sum_{k\neq j} m_{jk}b_k + a^2 - 2\delta_j a \qquad j = 1, \ldots, D. \tag{S20}$$

In the cycle (see Figure S1), we have $m_{ii} = 1 - m_A - m_C$. Hence, Eq. S20 becomes:

$$\begin{cases} (m_A + m_C)b_1 = m_A b_2 + m_C b_D + a^2 - 2\delta_1 a\,, \\ (m_A + m_C)b_j = m_A b_{j+1} + m_C b_{j-1} + a^2 - 2\delta_j a \qquad j = 2, \ldots, D-1\,, \\ (m_A + m_C)b_D = m_A b_1 + m_C b_{D-1} + a^2 - 2\delta_D a\,. \end{cases} \tag{S21}$$

The sum of Eqs. S21 reads:

$$\sum_{j=1}^{D}(m_A+m_C)b_j=\sum_{j=1}^{D}(m_A+m_C)b_j+Da^2-2a\sum_{j=1}^{D}\delta_j. \tag{S22}$$

Thus, $a=2\langle\delta\rangle$, which entails that the mutant fixation probability ρ_i to first order in st is

$$\rho_i=2\langle\delta\rangle st\,,\quad \text{for all } i\,. \tag{S23}$$

Again, this coincides with our general result for circulations in Eq. S16.

2.1.3 Beyond the circulation theorem: second order in st

Does the circulation theorem hold for heterogeneous environments beyond the first order in st? To investigate this, we calculate the second-order coefficient of the fixation probability. We start from the second line of Eq. S8, written in matrix form as:

$$\boldsymbol{b}=M\boldsymbol{b}+a^2\mathbf{1}-2a\boldsymbol{\delta}\,. \tag{S24}$$

Here, we introduced the vector of second-order coefficients $\boldsymbol{b}=(b_1,\dots,b_D)$, as well as the vectors $\boldsymbol{\delta}=(\delta_1,\dots,\delta_D)$, and $\mathbf{1}=(1,\dots,1)$. Meanwhile, M is the migration matrix with coefficients m_{ij}, and $a=2\langle\delta\rangle$ is the first-order coefficient (see Section 2.1.1). Recall that the first line of Eq. S8 implies that $\mathbf{1}$ is an eigenvector of M with eigenvalue 1.

To determine $\boldsymbol{b}$, we generalize the approach developed for homogeneous environments in Ref. [1], and use a Jordan decomposition of the migration matrix M. It allows us to complete $\mathbf{1}$ into a complex basis such that the complementary space $\mathcal{E}_\perp$ of the space $\mathcal{E}$ generated by $\mathbf{1}$ is stable under the action of M. We decompose $\boldsymbol{b}$ as follows: $\boldsymbol{b}=\boldsymbol{b}_\perp+\boldsymbol{b}_\parallel$, with $\boldsymbol{b}_\parallel=b_\parallel\mathbf{1}\in\mathcal{E}$ and $\boldsymbol{b}_\perp\in\mathcal{E}_\perp$. Similarly, we write $\boldsymbol{\delta}=\boldsymbol{\delta}_\perp+\boldsymbol{\delta}_\parallel$, with $\boldsymbol{\delta}_\parallel=\delta_\parallel\mathbf{1}$. We can then rewrite Eq. S24 as:

$$\boldsymbol{b}_\parallel+\boldsymbol{b}_\perp=M\boldsymbol{b}_\parallel+M\boldsymbol{b}_\perp+a^2\mathbf{1}-2a\boldsymbol{\delta}_\parallel-2a\boldsymbol{\delta}_\perp. \tag{S25}$$

Thus, in $\mathcal{E}$, since $M\boldsymbol{b}_\parallel=\boldsymbol{b}_\parallel$:

$$\begin{aligned}&\boldsymbol{b}_\parallel=\boldsymbol{b}_\parallel+a^2\mathbf{1}-2a\boldsymbol{\delta}_\parallel\,,\ \text{ i.e.}\\ &a^2=2a\delta_\parallel,\end{aligned} \tag{S26}$$

and in $\mathcal{E}_\perp$:

$$\boldsymbol{b}_\perp=M\boldsymbol{b}_\perp-2a\boldsymbol{\delta}_\perp. \tag{S27}$$

Since $a=2\langle\delta\rangle$ (see Section 2.1.1), Eq. S26 ensures that $\delta_\parallel=\langle\delta\rangle$. Thus, we have:

$$\boldsymbol{\delta}_\perp=\boldsymbol{\delta}-\boldsymbol{\delta}_\parallel=(\delta_1-\langle\delta\rangle,\delta_2-\langle\delta\rangle,\dots,\delta_D-\langle\delta\rangle)\,. \tag{S28}$$

As shown in Section 2.1.1, the largest eigenvalue of M is 1 and is associated to the eigenspace $\mathcal{E}_\parallel$, and all other eigenvalues have a strictly smaller modulus. Thus, $(\mathbb{I}-M)$ restricted to $\mathcal{E}_\perp$, where $\mathbb{I}$ denotes the identity matrix, has no zero eigenvalue, and is invertible. Hence, Eq. S27 yields:

$$\boldsymbol{b}_\perp=-4\langle\delta\rangle(\mathbb{I}-M)^{-1}\boldsymbol{\delta}_\perp\,, \tag{S29}$$

with $\boldsymbol{\delta}_\perp$ given in Eq. S28.

Next, to find $b_\parallel$, we use the third line of Eq. S8. Using $\sum_k m_{kj}=1$, and $a_j=a$ for all j, the third line of Eq. S8 becomes, for all $j\in[1,D]$,

$$\sum_{k=1}^{D}m_{kj}c_j=\sum_{k=1}^{D}m_{jk}c_k-2a^3+3ab_j-3\delta_jb_j+3\delta_ja^2-3\delta_j^2a\,. \tag{S30}$$

Summing this equation over all $j\in[1,D]$ yields:

$$\sum_{j=1}^{D}\sum_{k=1}^{D}m_{kj}c_j=\sum_{j=1}^{D}\sum_{k=1}^{D}m_{jk}c_k-2Da^3+3a\sum_{j=1}^{D}b_j-3\sum_{j=1}^{D}\delta_jb_j+3a^2\sum_{j=1}^{D}\delta_j-3a\sum_{j=1}^{D}\delta_j^2\,, \tag{S31}$$

which reduces to

$$3\sum_{j=1}^{D} b_j(a-\delta_j) = 2Da^3 - 3a^2D\langle\delta\rangle + 3a\sum_{j=1}^{D}\delta_j^2\,. \tag{S32}$$

Writing $b_j = b_\parallel + b'_j$ for all j, where b'_j is the j-th component of $\boldsymbol{b}_\perp$, and recalling that for circulations $a = 2\langle\delta\rangle$ (see Section 2.1.1), we obtain:

$$b_\parallel = \frac{4}{3}\langle\delta\rangle^2 + 2\langle\delta^2\rangle - \frac{1}{D}\sum_j b'_j\left(2-\frac{\delta_j}{\langle\delta\rangle}\right) = \frac{4}{3}\langle\delta\rangle^2 + 2\langle\delta^2\rangle - 2\langle b'\rangle + \frac{\langle b'\delta\rangle}{\langle\delta\rangle}\,, \tag{S33}$$

where b'_j can be obtained from Eq. S29.

Clique with heterogeneous environment. Recall that in the clique (see Figure 1B), $m_{ij} = m$ for all $i \neq j$. For a heterogeneous clique, $\boldsymbol{b}_\perp$ with components

$$b'_i = 4\langle\delta\rangle\frac{\langle\delta\rangle - \delta_i}{Dm} \tag{S34}$$

satisfies Eq. S29.

Let us now calculate $b_\parallel$ for a heterogeneous clique, using Eq. S33. Eq. S34 entails that $\langle b'\rangle = 0$, and allows us to write:

$$\langle b'\delta\rangle = \frac{1}{D}\sum_j b'_j\delta_j = \frac{4\langle\delta\rangle}{D^2m}\sum_j\left(\langle\delta\rangle-\delta_j\right)\delta_j = \frac{4\langle\delta\rangle}{Dm}\left(\langle\delta\rangle^2 - \langle\delta^2\rangle\right), \tag{S35}$$

so Eq. S33 yields:

$$b_\parallel = \frac{4}{3}\langle\delta\rangle^2 + 2\langle\delta^2\rangle + \frac{4}{Dm}\left(\langle\delta\rangle^2 - \langle\delta^2\rangle\right)\,. \tag{S36}$$

Eq. S34 and Eq. S36 show that, to second order in st, the mutant fixation probability in a heterogeneous clique generically depends on the deme i where the mutant starts, and on migration intensity m. Therefore, the circulation theorem does not hold to second order in st for heterogeneous structures.

In the particular case of the homogeneous clique with $\delta_i = \Delta$ for all i, Eq. S34 reduces to $b'_i = 0$ for all i, and Eq. S36 reduces to $b_\parallel = 10\Delta^2/3$, yielding a mutant fixation probability $\rho_i = 2\Delta st - 5(\Delta st)^2/3 + o((st)^2)$ for all i. This is in agreement with the result of Ref. [1] for homogeneous circulations (see Eq. S16 in the Supplementary Material of that paper, written for a mutant with effective fitness advantage Δst). Recall that the circulation theorem holds for homogeneous environments in the branching process approximation [1].

Note that the mutant fixation probability averaged over starting demes reads:

$$\rho = 2\langle\delta\rangle st - \frac{b_\parallel + \langle b'\rangle}{2}(st)^2 + o((st)^2) = 2\langle\delta\rangle st - \frac{b_\parallel}{2}(st)^2 + o((st)^2)\,, \tag{S37}$$

where $b_\parallel$ is given by Eq. S36. Thus, to second order, this fixation probability does not involve $\boldsymbol{b}_\perp$, and only depends on environment heterogeneity through $\langle\delta\rangle$ and $\langle\delta^2\rangle$.

Let us compare the mutant fixation probability, averaged over starting demes, in a heterogeneous clique and in a homogeneous clique with the same $\langle\delta\rangle$. Calling ρ and ρ^{homo} these two fixation probabilities, Eq. S37 yields:

$$\begin{aligned}\rho - \rho^{\text{homo}} &= \frac{(st)^2}{2}\left(b_\parallel^{\text{homo}} - b_\parallel\right) = \frac{(st)^2}{2}\left[\frac{10}{3}\langle\delta\rangle^2 - \frac{4}{3}\langle\delta\rangle^2 - 2\langle\delta^2\rangle - \frac{4}{Dm}\left(\langle\delta\rangle^2 - \langle\delta^2\rangle\right)\right]\\ &= \frac{2-Dm}{Dm}(st)^2\left(\langle\delta^2\rangle - \langle\delta\rangle^2\right)\,.\end{aligned} \tag{S38}$$

In the clique, $\sum_i m_{ij} = 1$ entails that $m_{ii} = 1-(D-1)m$, and as all migration probabilities are between 0 and 1, we have $(D-1)m \leq 1$ and, and thus $Dm \leq 1+m \leq 2$. This entails that $\rho \geq \rho^{\text{homo}}$.

Thus, in the clique, the fixation probability averaged over all initial mutant locations is increased by environment heterogeneity, compared to that of a homogeneous clique with the same $\langle\delta\rangle$. In other words, environment heterogeneity generically amplifies natural selection to second order in st. Furthermore, this effect increases with the environment variance $\mathrm{Var}(\delta)$ of the δ_i coefficients across demes is. It also increases when the migration probability m decreases. Figure S3A shows the second-order coefficient in st of the fixation probability. Besides being increased by environment heterogeneity, we observe that it can be positive, in which case the second order term increases the fixation probability, while for homogeneous environments, it always decreases it.

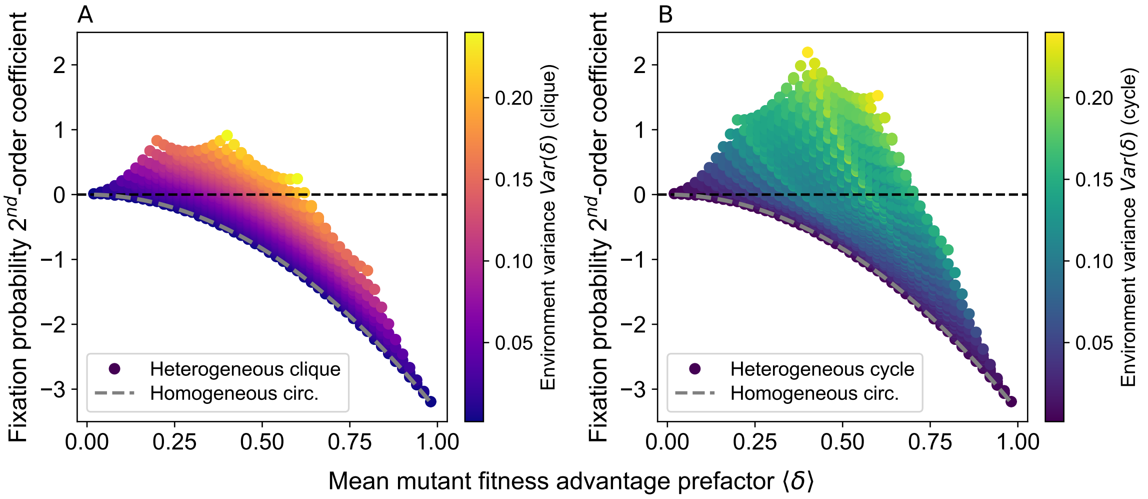

Figure S3: **Impact of environment heterogeneity on fixation probability in circulations.** As the fixation probability is the same in all circulations to first order in st, we focus on the impact of the second-order term. Specifically, we show the second-order coefficient $-b_{\|}$ in the expansion of fixation probability (Eqs. S37 and S42; see also Eq. 1 in the main text). Panel A: This second-order coefficient (expressed using Eq. S36) is shown versus the mean mutant fitness advantage prefactor $\langle\delta\rangle$ across the structure, for various heterogeneous cliques. Markers are colored according to the variance of the deme-specific mutant fitness advantage prefactor δ_i coefficient across demes i, a proxy for environment heterogeneity. Horizontal dashed black line: no deviation from the first-order approximation. Dashed gray line: homogeneous environment case. Panel B: Same as A, but for heterogeneous cycles with equal clockwise and anticlockwise migration probabilities (using Eq. S41). Parameters: $D = 5$, $m = 0.1$ in the clique, $m = 0.2$ in the cycle (same total of exchanges in the two structures). Numbers in $\{0, 0.1, \ldots, 0.9, 1\}$ are employed for each δ_i, and all combinations of them are considered.

A particular cycle with heterogeneous environment. Recall that in the cycle, migration probabilities are nonzero only to nearest neighbor demes (and to the original deme itself). Migrations to nearest neighbor demes can differ in the clockwise and anticlockwise directions, and are denoted by m_C and m_A, respectively (see Figure 1B). Here, we present explicit calculations in the specific case of a heterogeneous cycle with $D = 5$ and $m_A = m_C = m$. Solving Eq. S29 in this case leads to:

$$\boldsymbol{b}_\perp = \begin{pmatrix} b'_1 \\ b'_2 \\ b'_3 \\ b'_4 \\ b'_5 \end{pmatrix} = \frac{4\langle\delta\rangle}{5m} \begin{pmatrix} \delta_3 + \delta_4 - 2\delta_1 \\ \delta_4 + \delta_5 - 2\delta_2 \\ \delta_5 + \delta_1 - 2\delta_3 \\ \delta_1 + \delta_2 - 2\delta_4 \\ \delta_2 + \delta_3 - 2\delta_5 \end{pmatrix} . \tag{S39}$$

Let us now calculate $b_{\|}$ in this case, using Eq. S33. Eq. S39 entails that $\langle b'\rangle = 0$, and allows us to write:

$$\langle b'\delta\rangle = \frac{1}{5}\sum_{j=1}^{5} b'_j\delta_j = -\frac{8\langle\delta\rangle}{25m}\left(5\langle\delta^2\rangle - \sum_{j=1}^{5}\delta_j\delta_{(j+2)\,\mathrm{mod}\,5}\right), \tag{S40}$$

so Eq. S33 yields:

$$b_{\|} = \frac{4}{3}\langle\delta\rangle^2 + 2\langle\delta^2\rangle - \frac{8}{25m}\left(5\langle\delta^2\rangle - \sum_{j=1}^{5}\delta_j\delta_{(j+2)\,\mathrm{mod}\,5}\right). \tag{S41}$$

Note that in a homogeneous cycle with $D = 5$ and $m_A = m_C = m$, where $\delta_i = \Delta$ for all i, Eq. S39 reduces to $\boldsymbol{b}_\perp = \mathbf{0}$, and Eq. S41 reduces to $b_{\|} = 10\Delta^2/3$, which is the same as above for the homogeneous clique. This is consistent with the fact that the circulation theorem holds for homogeneous environments in the branching process approximation [1].

The mutant fixation probability averaged over starting demes reads:

$$\rho = 2\langle\delta\rangle st - \frac{b_\parallel + \langle b' \rangle}{2}(st)^2 + o((st)^2) = 2\langle\delta\rangle st - \frac{b_\parallel}{2}(st)^2 + o((st)^2)\,, \quad \text{(S42)}$$

where $b_\parallel$ is given by Eq. S40. As for the clique, to second order, this fixation probability does not involve $\boldsymbol{b}_\perp$.

Let us compare the mutant fixation probability, averaged over starting demes, in a heterogeneous cycle and in a homogeneous cycle with the same $\langle\delta\rangle$, still in the particular case where $D = 5$ and $m_A = m_C = m$. Calling ρ and ρ^{homo} these two fixation probabilities, Eq. S42 yields:

$$\begin{aligned}\rho - \rho^{\text{homo}} &= \frac{(st)^2}{2}\left(b_\parallel^{\text{homo}} - b_\parallel\right) = \frac{(st)^2}{2}\left[\frac{10}{3}\langle\delta\rangle^2 - \frac{4}{3}\langle\delta\rangle^2 - 2\langle\delta^2\rangle + \frac{8}{25m}\left(5\langle\delta^2\rangle - \sum_{j=1}^{5}\delta_j\delta_{(j+2)\,\mathrm{mod}\,5}\right)\right] \\ &= (st)^2\left[\langle\delta\rangle^2 - \langle\delta^2\rangle + \frac{4}{25m}\left(5\langle\delta^2\rangle - \sum_{j=1}^{5}\delta_j\delta_{(j+2)\,\mathrm{mod}\,5}\right)\right]. \end{aligned} \quad \text{(S43)}$$

Let us now investigate the sign of $\rho - \rho^{\text{homo}}$. Noticing that

$$\left(\sum_{i=1}^{5}\delta_i\right)^2 = \sum_{i=1}^{5}\delta_i^2 + 2\sum_{i=1}^{5}\delta_i\delta_{(i+1)\,\mathrm{mod}\,5} + 2\sum_{i=1}^{5}\delta_i\delta_{(i+2)\,\mathrm{mod}\,5}\,, \quad \text{(S44)}$$

we can write, in terms of rough orders of magnitude,

$$\sum_{i=1}^{5}\delta_i\delta_{(i+2)\,\mathrm{mod}\,5} \simeq \sum_{i=1}^{5}\delta_i\delta_{(i+1)\,\mathrm{mod}\,5} \simeq \frac{1}{4}\left[\left(\sum_{i=1}^{5}\delta_i\right)^2 - \sum_{i=1}^{5}\delta_i^2\right] = \frac{25}{4}\langle\delta\rangle^2 - \frac{5}{4}\langle\delta^2\rangle\,. \quad \text{(S45)}$$

Eq. S43 then yields:

$$\rho - \rho^{\text{homo}} \simeq (st)^2\left[\langle\delta\rangle^2 - \langle\delta^2\rangle + \frac{4}{25m}\left(5\langle\delta^2\rangle - \frac{25}{4}\langle\delta\rangle^2 + \frac{5}{4}\langle\delta^2\rangle\right)\right] \simeq \frac{1-m}{m}(st)^2\left(\langle\delta^2\rangle - \langle\delta\rangle^2\right). \quad \text{(S46)}$$

In our particular cycle with $m_A = m_C = m$, the condition $\sum_i m_{ij} = 1$ for all j gives $2m + m_{ii} = 1$, i.e. $m_{ii} = 1 - 2m$. Since all migration probabilities are between 0 and 1, we have $m \leq 1/2$. Hence, under the rough assumption of Eq. S45, Eq. S46 indicates that $\rho \gtrsim \rho^{\text{homo}}$ and that the smaller m is, and the larger the variance of the δ_i is, the larger $\rho - \rho^{\text{homo}}$ is expected to become. These approximate results are reminiscent of the exact results obtained for the clique in Eq. S38. Accordingly, we observe in Figure S3B a similar behavior of the second-order coefficient in st of the fixation probability in this particular cycle as in the clique (considered in Figure S3A).

Figure S4A shows the exact value of the deviation with respect to the homogeneous case of $b_\parallel$, from Eq. S43, for multiple heterogeneous cycles, differing by their environmental heterogeneity, via $\boldsymbol{\delta}$. We observe that the approximate second-order coefficient, from Eq. S46, yields the correct trend of its dependence in $\mathrm{Var}(\delta) = \langle\delta^2\rangle - \langle\delta\rangle^2$ (in agreement with a linear fit), but that there is substantial spread around that trend. We also observe that the discrepancy between the exact and the approximate formulas is small (resp. large) when $\left|\sum_{i=1}^5 \delta_i\delta_{(i+2)\,\mathrm{mod}\,5} - \sum_{i=1}^5 \delta_i\delta_{(i+1)\,\mathrm{mod}\,5}\right|$ is small (resp. large), as expected given the approximation made.

Since for the cycle the variance does not fully capture the impact of environment heterogeneity, we further explore the role of the spatial placement of heterogeneity in the cycle. A metric to quantify local variation between neighboring demes compared to the global variation is:

$$V = \frac{\left(\sum_{i=1}^5 |\delta_i - \delta_{(i+1)\,\mathrm{mod}\;5}|\right)^2}{5\mathrm{Var}(\delta)}, \quad \text{(S47)}$$

where δ_i denotes the deme-specific mutant fitness advantage prefactor, and $\mathrm{Var}(\delta)$ is the variance of these prefactors across demes. The numerator of V quantifies local environment variation, and the denominator overall environment heterogeneity. At a fixed value of the variance, a gradual local

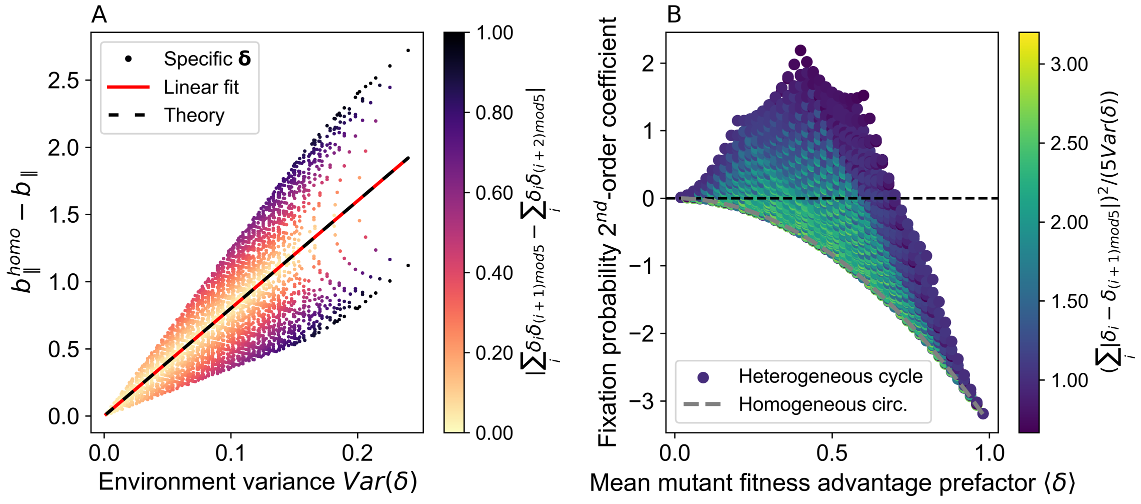

Figure S4: **Impact of environment heterogeneity on the second-order coefficient of the fixation probability in the cycle.** Panel A: Difference $b_{\parallel}^{\mathrm{homo}} - b_{\parallel}$ between the second-order coefficients for a cycle with heterogeneous and homogeneous environment (see Eq. S43), plotted against the environment variance $\mathrm{Var}(\delta)$. Markers are colored according to $\left|\sum_{i=1}^{5} \delta_i \delta_{(i+2) \bmod 5} - \sum_{i=1}^{5} \delta_i \delta_{(i+1) \bmod 5}\right|$. Solid red line: linear fit of the data. Dashed black line: approximate theoretical prediction from Eq. S46. Panel B: The second-order coefficient $-b_{\parallel}$ in the expansion of fixation probability (Eq. S42; see also Eq. 1 in the main text) is shown versus the mean mutant fitness advantage prefactor $\langle\delta\rangle$ across the structure, for various heterogeneous cycles, as in Figure S3B, but colored according to the ratio of local to global environmental variation, defined in Eq. S47. Dashed gray line: homogeneous environment case (Eq. S36). Parameter values for both panels: $D = 5$, $m_A = m_C = m = 0.2$ for the cycle. Numbers in $\{0, 0.1, \ldots, 0.9, 1\}$ are employed for each δ_i, and all combinations of them are considered. All cycles considered here have equal clockwise and anticlockwise migration probabilities.

variation results in a small numerator, resulting in a small value of V. Meanwhile, repeated abrupt alternations in δ_i for nearby demes result in a large numerator and hence a large value of V. In Figure S4B, we color the excess fixation probability in the cycle due to the second order term (i.e. $-b_{\parallel}$) based on the value of V in Eq. S47. We observe that when similar values of δ_i are clustered together (i.e. for small V), the excess fixation probability tends to be higher.

2.2 Star with heterogeneous environment

Let us now consider the star, with incoming migration probabilities from a leaf to the center that can differ from outgoing ones from the center to a leaf, and are denoted by m_I and m_O, respectively (see Figure 1B).

As noted earlier, the first line in Eq. S8 is not impacted by environment heterogeneity. Thus, as in Ref. [1], the first-order coefficients for the fixation probability for a mutant starting in the center and in a leaf, denoted respectively by a_C and a_L, satisfy $a_L = \alpha a_C$, where $\alpha = m_I/m_O$ represents migration asymmetry. Note that environment heterogeneity across leaves does not make these first-order coefficients dependent on the leaf where the mutant start.

Let $\mathcal{L}$ be the set of the indexes labeling the leaves of the star. The second line in Eq. S8, written for the center (with second-order coefficient b_C) and for one of the leaves $j \in \mathcal{L}$ (with second-order coefficient b_j), yields:

$$\begin{cases} (D-1)m_I b_C = m_O \sum_{k\in\mathcal{L}} b_k + a_C^2 - 2\delta_C a_C \,, \\ m_O b_j = m_I b_C + a_L^2 - 2\delta_j a_L \,, \qquad j \in \mathcal{L}. \end{cases} \tag{S48}$$

Summing Eqs. S48 over all demes gives:

$$(D-1)m_I b_C + m_O \sum_{j\in\mathcal{L}} b_j = (D-1)m_I b_C + m_O \sum_{k\in\mathcal{L}} b_k + a_C^2 + (D-1)a_L^2 - 2\delta_C a_C - 2\sum_{j\in\mathcal{L}} \delta_j a_L \,, \tag{S49}$$

i.e., after simplification, and using $a_L = \alpha a_C$:

$$a_C^2[1 + \alpha^2(D - 1)] = 2a_C\delta_C + 2\alpha a_C \sum_{j\in\mathcal{L}} \delta_j . \tag{S50}$$

Hence, as $a_C \neq 0$, we obtain:

$$a_C = \frac{2(\delta_C + \alpha \sum_{i\in\mathcal{L}} \delta_i)}{1 + \alpha^2(D - 1)} \quad \text{and} \quad a_L = \alpha a_C \, . \tag{S51}$$

Let us now consider a mutant appearing at a bottleneck in a deme chosen uniformly at random. Its fixation probability ρ reads, to first order in st:

$$\begin{aligned} \rho &= st \, \frac{a_C + (D-1)a_L}{D} = 2st \, \frac{[1 + \alpha(D-1)](\delta_C + \alpha \sum_{i\in\mathcal{L}} \delta_i)}{D[1 + \alpha^2(D-1)]} \\ &= 2\langle\delta\rangle st \, \frac{[1 + \alpha(D-1)][\sigma_C(1-\alpha) + 1 + \alpha(D-1)]}{D[1 + \alpha^2(D-1)]} \, , \end{aligned} \tag{S52}$$

where we introduced $\langle\delta\rangle = \sum_{i=1}^{D} \delta_i/D$, and $\sigma_C = (\delta_C - \langle\delta\rangle)/\langle\delta\rangle$. Importantly, the result in Eq. S52 only depends on selection through the mean mutant fitness effect, via $\langle\delta\rangle$, and through the relative fitness excess in the center, via σ_C. In other words, the heterogeneity of selection across leaves does not impact this fixation probability to first order. We show the first-order coefficient of the fixation probability as a function of α and σ_C in Figure 2A.

Note that for a homogeneous star, where $\sigma_C = 0$ and δ takes a unique value, say Δ, across the whole population, Eq. S52 reduces to:

$$\rho = 2\Delta st \, \frac{[1 + \alpha(D-1)]^2}{D[1 + \alpha^2(D-1)]} \, , \tag{S53}$$

i.e. the result from Ref. [1] for the homogeneous star. Note also that for $\alpha = 1$, when the star is a circulation, Eq. S52 yields the same result as for other circulations considered so far, namely $\rho = 2\langle\delta\rangle st$ (see Eqs. S19 and S23).

2.3 Line with heterogeneous environment

Let us now consider the line, with migration probabilities to the left that can differ from those to the right, and are denoted by m_L and m_R, respectively (see Figure 1B). Contrary to other structures discussed so far, the line was not considered in Ref. [1] in the homogeneous case. Hence, the results below are new even in the homogeneous case.

The first line in Eq. S8, written for each node of the line, yields:

$$\begin{cases} a_1 = (1 - m_L)a_1 + m_R a_2 \, , \\ a_j = (1 - m_L - m_R)a_j + m_R a_{j+1} + m_L a_{j-1} \qquad j = 2, \dots, D-1 \, , \\ a_D = (1 - m_R)a_D + m_L a_{D-1} \, . \end{cases} \tag{S54}$$

Introducing migration asymmetry $\alpha = m_R/m_L$, we get to:

$$\begin{cases} a_1 = \alpha a_2 \, , \\ a_j(1 + \alpha) = \alpha a_{j+1} + a_{j-1} \qquad j = 2, \dots, D-1 \, , \\ a_D = a_{D-1}/\alpha \, . \end{cases} \tag{S55}$$

It can be shown by induction that

$$a_j = \frac{B}{\alpha^{j-1}} \, , \tag{S56}$$

where B is a constant, is a solution of Eq. S55.

Let us now use the equations corresponding to the second line of Eq. S8, written for each node of the line, to determine the values of the constant B:

$$\begin{cases} b_1 m_L = m_R b_2 + a_1^2 - 2\delta_1 a_1 \, , \\ b_j(m_L + m_R) = m_R b_{j+1} + m_L b_{j-1} + a_j^2 - 2\delta_j a_j \qquad j = 2, \dots, D-1 \, , \\ b_D m_R = m_L b_{D-1} + a_D^2 - 2\delta_D a_D \, . \end{cases} \tag{S57}$$

Summing Eqs. S57 over all nodes of the line yields:

$$m_L b_1 + \sum_{j=2}^{D-1} b_j (m_L + m_R) + b_D m_R = m_R b_2 + m_L b_{D-1} + \sum_{j=2}^{D-1} b_{j+1} m_R + \sum_{j=2}^{D-1} b_{j-1} m_L + \sum_{j=1}^{D} a_j^2 - 2 \sum_{j=1}^{D} \delta_j a_j \,, \tag{S58}$$

which simplifies into

$$\sum_{j=1}^{D} a_j^2 = 2 \sum_{j=1}^{D} \delta_j a_j \,. \tag{S59}$$

Using Eqs. S56 and S59 then gives:

$$B = 2 \frac{\sum_{j=1}^{D} \delta_j \alpha^{-j+1}}{\sum_{j=1}^{D} \alpha^{-2j+2}} = 2 \frac{\alpha^2 - 1}{\alpha^2 - \alpha^{2(1-D)}} \sum_{j=1}^{D} \delta_j \alpha^{-j+1}, \tag{S60}$$

The first-order coefficient a_k of the fixation probability for a mutant starting in deme k, thus reads:

$$a_k = \frac{B}{\alpha^{k-1}} = 2\alpha^{-k} \frac{\alpha^2 - 1}{1 - \alpha^{-2D}} \sum_{j=1}^{D} \delta_j \alpha^{-j}. \tag{S61}$$

Let us now consider a mutant appearing at a bottleneck in a deme chosen uniformly at random. Its fixation probability ρ reads, to first order in st:

$$\rho = st \frac{1}{D} \sum_{k=1}^{D} a_k = \frac{2st}{D} \frac{1+\alpha}{1+\alpha^{-D}} \sum_{j=1}^{D} \delta_j \alpha^{-j}. \tag{S62}$$

In particular, for a homogeneous line where all δ_i are equal, say to Δ, this reduces to:

$$\rho = \frac{2\Delta st}{D} \frac{1+\alpha}{1+\alpha^{-D}} \sum_{j=1}^{D} \alpha^{-j} = \frac{2\Delta st}{D} \frac{(1+\alpha)(1-\alpha^D)}{(1-\alpha)(1+\alpha^D)} \,. \tag{S63}$$

Note that if $\alpha = 1$, when the line is a circulation, Eq. S56 reduces to $a_j = C$, with C a constant. Then, Eq. S59 yields $C = 2\langle\delta\rangle$, and hence $\rho = 2\langle\delta\rangle st$, which is the same result as for the other circulation graphs we considered so far.

3 Comparison of fixation probabilities between structures

3.1 Comparison of fixation probabilities in the star and in the clique

Let us focus on the heterogeneous star structure, and on its impact on mutant fixation probability. First, for reference, in Section 3.1.1, we briefly compare a homogeneous star to a clique. Next, in Section 3.1.2, we determine under what conditions heterogeneity can increase the fixation probability in the star, compared to a homogeneous star. Finally, in Section 3.1.3, we compare a heterogeneous star to a clique, to determine if a heterogeneous star can suppress or amplify selection.

In all cases, when we compare a structure with heterogeneous environment to another one with homogeneous environment, we match their average mutant fitness advantage, i.e. in our notations, we match their $\langle\delta\rangle$. Our comparisons all rely on the first-order fixation probabilities derived in Section 1 in the branching process approximation.

3.1.1 Homogeneous star versus clique

We first consider a homogeneous star with relative mutant fitness advantage $s\Delta$ in all demes. We compare it to a heterogeneous clique with relative mutant fitness advantage $s\delta_i$ in deme i, satisfying $\langle\delta\rangle = \Delta$. Using the fixation probabilities in Eqs. S53 and S19, we find that the homogeneous star has a first-order fixation probability lower than the heterogeneous clique if and only if:

$$\frac{1}{D} \frac{[1+(D-1)\alpha]^2}{[1+\alpha^2(D-1)]} < 1 \,. \tag{S64}$$

Note that the heterogeneity of the clique does not impact its fixation probability to first order (see Eq. S19), and thus, this condition is the same as that found in Ref. [1], when comparing the homogeneous star and the homogeneous clique. As shown in Ref. [1], this condition is always satisfied, except for $\alpha = 1$, when the two fixation probabilities are equal. Thus, the homogeneous star suppresses natural selection under frequent asymmetric migrations.

3.1.2 Homogeneous star versus heterogeneous star

Let us compare the fixation probabilities in a heterogeneous star and in a homogeneous star, given respectively in Eqs. S52 and S53 to first order in st. Our homogeneous star has relative mutant fitness advantage $s\Delta$ in all demes, while our heterogeneous star has relative mutant fitness advantage $s\delta_i$ in deme i, satisfying $\langle\delta\rangle = \Delta$. The first-order fixation probability is larger in the heterogeneous star than in the homogeneous star if and only if:

$$\frac{[1+\alpha(D-1)][\sigma_C(1-\alpha)+1+\alpha(D-1)]}{D[1+\alpha^2(D-1)]} > \frac{[1+\alpha(D-1)]^2}{D[1+\alpha^2(D-1)]}\,, \tag{S65}$$

As the denominator and the term $1+\alpha(D-1)$ are positive, this is equivalent to

$$\sigma_C(1-\alpha) > 0\,. \tag{S66}$$

Thus, the relative fitness excess in the center should have the same sign as $1-\alpha$ for heterogeneity to enhance mutant fixation probability. This means that if $\alpha < 1$ (i.e. $m_I < m_O$, more outflow from the center than inflow), heterogeneity benefits mutant fixation if the center features a larger mutant fitness advantage than the leaves. Conversely, if $\alpha > 1$ (i.e. $m_I > m_O$, more inflow to the center than outflow), heterogeneity benefits mutant fixation if the center features a smaller mutant fitness advantage than the leaves.

3.1.3 Heterogeneous star versus clique

We have shown that a homogeneous star has a smaller mutant fixation probability than a clique, but that a heterogeneous star can have a larger mutant fixation probability than a homogeneous one. What is the overall effect of a heterogeneous star structure compared to a clique? In particular, can environment heterogeneity allow the star to overcome the suppression of selection effect it has under homogeneous environments? To address this question, we compare a heterogeneous star to a clique. Recall that only average fitness matters to first order for the mutant fixation probability in the clique. Hence, we consider a clique which can be homogeneous or heterogeneous, but which has the same average fitness as our heterogeneous star.

The fixation probabilities in a heterogeneous star and in a clique are given respectively in Eqs. S52 and S19 to first order in st. The first-order fixation probability is larger in the heterogeneous star than in a clique if and only if:

$$\frac{[1+\alpha(D-1)][\sigma_C(1-\alpha)+1+\alpha(D-1)]}{D[1+\alpha^2(D-1)]} > 1\,. \tag{S67}$$

This condition is equivalent to:

$$\begin{aligned}&\sigma_C(1-\alpha)[1+\alpha(D-1)] > D[1+\alpha^2(D-1)] - [1+\alpha(D-1)]^2\,,\ \text{i.e.}\\ &\sigma_C(1-\alpha)[1+\alpha(D-1)] > (D-1)(1-\alpha)^2\,.\end{aligned} \tag{S68}$$

The condition in Eq. S68 can be expressed as:

- $\sigma_C > f_{\text{star}}(\alpha, D)$ if $\alpha < 1$,
- $\sigma_C < f_{\text{star}}(\alpha, D)$ if $\alpha > 1$,

where we introduced

$$f_{\text{star}}(\alpha, D) = \frac{(D-1)(1-\alpha)}{1+\alpha(D-1)}\,. \tag{S69}$$

We show $f_{\text{star}}(\alpha, D)$ versus α in Figure S5A, and highlight in pink the regions of the plane (α, σ_C) where amplification of selection exists (see also Figure 2). We further present an example of amplification of selection in Figure S5B. The associated fixation and extinction times are shown in Figure S5C-D. As observed with the line in the main text (see Figure 3), we simultaneously obtain amplification of selection and acceleration of fixation and extinction, albeit to a smaller extent.

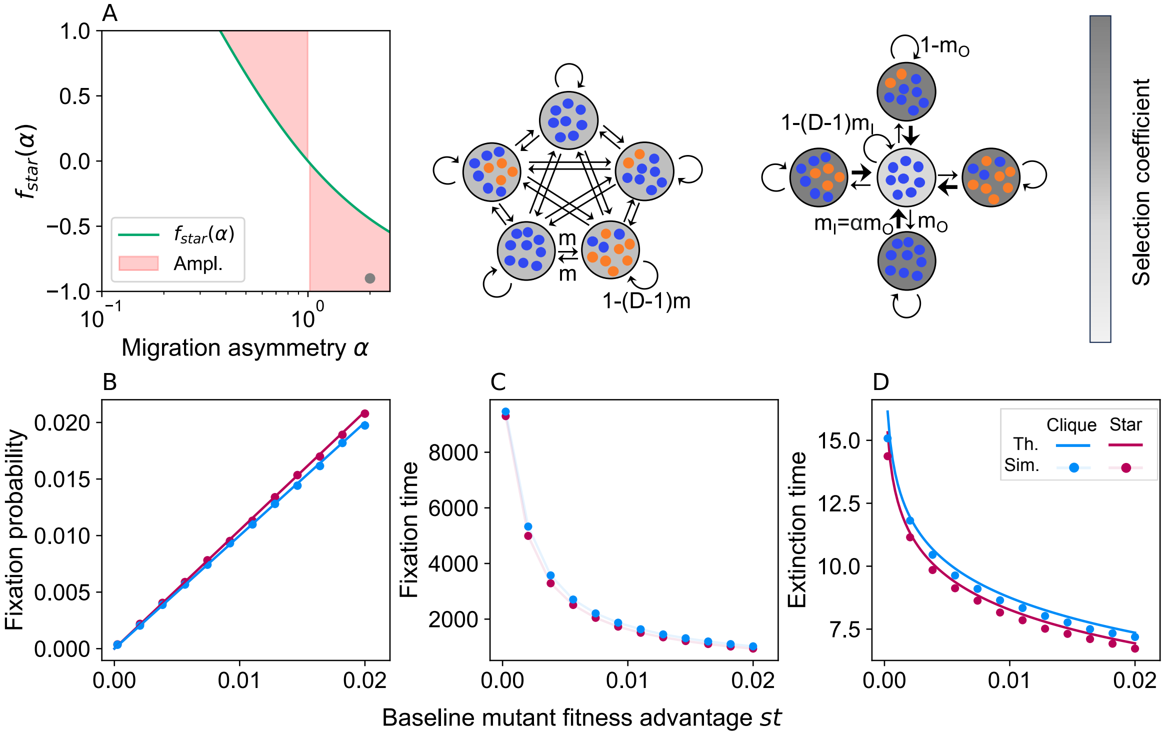

Figure S5: **Amplification of natural selection by the heterogeneous star with frequent migrations.** Panel A: $f_{\text{star}}(\alpha, D = 5)$ defined in Eq. S69 is plotted as a function of α. The pink region highlights the parameter range where Eq. S68 predicts that a heterogeneous star amplifies selection relative to a clique. The gray marker indicates the parameter set used in the other panels. Panel B: Fixation probability for a heterogeneous star and a clique with the same $\langle\delta\rangle$ as a function of the baseline mutant fitness advantage st. Panels C-D: Mutant fixation and extinction time, respectively, expressed in numbers of bottlenecks, as a function of the baseline mutant fitness st. Results from the branching process theory ("Th.") are shown in panels B (Eq. S52) and D (Eq. S13). Stochastic simulation results ("Sim.") are shown in panels B-D. Parameters: $D = 5$, $K = 1000$; for the star: $\alpha = 2$, $m_O = 0.1$; $\delta_C = 0.05$ in the center; $\delta_L = 0.6125$ in all leaves, yielding $\langle\delta\rangle = 0.5$ and $\sigma_C = -0.9$; for the clique: $\delta = 0.5$ in all demes and $m = 0.15$. Each marker comes from 5×10^5 stochastic simulation realizations.

3.2 Comparison of fixation probabilities in the line and in the clique

In this section, we focus on the line structure, and on its impact on mutant fixation probability. First, in Section 3.2.1, we compare a homogeneous line to a clique, to assess the baseline impact of the line spatial structure. Then, in Section 3.2.2 we compare a homogeneous line to a heterogeneous line, to assess the impact of environment heterogeneity in a line. Finally, in Section 3.2.3, we compare a heterogeneous line to a clique, to determine if a heterogeneous line can suppress or amplify selection.

As done above for the star, in all cases, when we compare a structure with heterogeneous environment to another one with homogeneous environment, we match their average mutant fitness advantage, i.e. in our notations, we match their $\langle\delta\rangle$. Besides, our comparisons again rely on the first-order fixation probabilities derived in Section 2 in the branching process approximation.

3.2.1 Homogeneous line versus clique

Let us consider a homogeneous line with relative mutant fitness advantage $s\Delta$ in all demes. Let us compare it to a heterogeneous clique with relative mutant fitness advantage $s\delta_i$ in deme i, satisfying $\langle\delta\rangle = \Delta$. We derived their fixation probabilities in Eqs. S63 and S19, respectively. Recall that the result for the clique was unaffected by environment heterogeneity – thus, our comparison will hold for homogeneous and heterogeneous cliques. A homogeneous line has a first-order mutant fixation probability lower than a heterogeneous clique if and only if:

$$\frac{1}{D}\frac{(1+\alpha)(1-\alpha^D)}{(1-\alpha)(1+\alpha^D)} < 1\,. \tag{S70}$$

This condition is always satisfied, except if $\alpha = 1$, when the two fixation probabilities are equal. It is illustrated in Figure S6. Hence, the homogeneous line suppresses selection, as long as it features migration asymmetry. This is consistent with our general result on homogeneous spatially structured populations from Ref. [1].

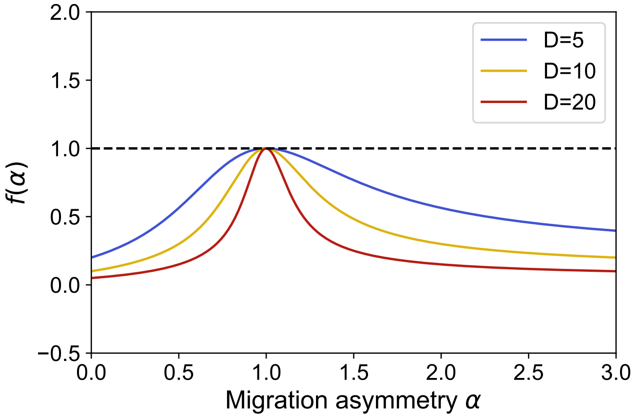

Figure S6: **Suppression of selection in the homogeneous line with migration asymmetry.** The function $f(\alpha) = (1+\alpha)(1-\alpha^D)/[D(1-\alpha)(1+\alpha^D)]$ involved in Eq. S70 is plotted versus the migration asymmetry α in the line for $D = 5, 10, 20$.

3.2.2 Homogeneous line versus heterogeneous line

Let us compare the fixation probabilities in a heterogeneous line and in a homogeneous line, given respectively in Eqs. S62 and S63 to first order in st. Our homogeneous line has relative mutant fitness advantage $s\Delta$ in all demes, while our heterogeneous line has relative mutant fitness advantage $s\delta_i$ in deme i, satisfying $\langle\delta\rangle = \Delta$. The first-order fixation probability is larger in the heterogeneous star than in the homogeneous star if and only if:

$$\frac{1}{1+\alpha^{-D}}\sum_{j=1}^{D}\delta_j\alpha^{-j} > \Delta\frac{(1-\alpha^D)}{(1-\alpha)(1+\alpha^D)}\,, \quad \text{i.e.}$$

$$\sum_{j=1}^{D}\frac{\delta_j}{\Delta}\alpha^{-j} > \frac{1-\alpha^{-D}}{\alpha-1}\,. \tag{S71}$$

Let us now introduce the relative mutant fitness excess σ_j in deme j such that for all j: $\delta_j = \langle\delta\rangle(1+\sigma_j) = \Delta(1+\sigma_j)$. Note that the σ_j satisfy $\sum_{j=1}^{D}\sigma_j = 0$. Eq. S71 then becomes:

$$\sum_{j=1}^{D}\alpha^{-j} + \sum_{j=1}^{D}\sigma_j\alpha^{-j} > \frac{1-\alpha^{-D}}{\alpha-1}\,, \quad \text{i.e.}$$

$$S(\alpha) \equiv \sum_{j=1}^{D}\sigma_j\alpha^{-j} > 0\,. \tag{S72}$$

Monotonic mutant fitness advantage variation along the line. Let us consider the case where the mutant fitness advantage varies monotonically along the line. Without loss of generality, let us assume that it monotonically decreases left to right, from deme 1 to deme D. Since the σ_j satisfy $\sum_{j=1}^{D} \sigma_j = 0$, there exists $k \in [1, D]$ such that

$$\sigma_1 > \dots \sigma_k \geq 0 > \sigma_{k+1} > \dots > \sigma_D . \tag{S73}$$

If $\alpha > 1$, then for all $j > k \geq 1$, we have $\alpha^{-j} < \alpha^{-k}$, while for all j satisfying $1 \leq j \leq k$, we have $\alpha^{-j} \geq \alpha^{-k}$. Combining this with Eq. S73 yields:

$$S(\alpha) = \sum_{j=1}^{k} \sigma_j \alpha^{-j} + \sum_{j=k+1}^{D} \sigma_j \alpha^{-j} > \alpha^{-k} \sum_{j=1}^{k} \sigma_j + \alpha^{-k} \sum_{j=k+1}^{D} \sigma_j = \alpha^{-k} \sum_{j=1}^{D} \sigma_j = 0 , \tag{S74}$$

which gives $S(\alpha) > 0$. Hence, Eq. S72 holds for all $\alpha > 1$ in our line with monotonically decreasing fitness advantage from left to right.

If $\alpha < 1$, a similar reasoning leads to $S(\alpha) < 0$. Hence, Eq. S72 never holds for $\alpha < 1$ in our line with monotonically decreasing fitness advantage from left to right.

Thus, the heterogeneous line with monotonically decreasing mutant fitness advantage from left to right features a larger mutant fixation probability than the homogeneous line for $\alpha > 1$, but a smaller one for $\alpha < 1$, and the same one for $\alpha = 1$. When $\alpha > 1$, most migrations are from left to right, i.e. demes where mutants are most advantaged are upstream of the overall migration flow. This favors mutant spread and fixation.

3.2.3 Heterogeneous line versus clique

Let us now compare a heterogeneous line to a clique. Recall that only average fitness matters to first order for the mutant fixation probability in the clique. Hence, we consider a clique which can be homogeneous or heterogeneous, but which has the same average fitness as our heterogeneous line. The fixation probabilities in a heterogeneous line and in a clique are given respectively in Eqs. S62 and S19 to first order in st. The first-order fixation probability is larger in the heterogeneous line than in a clique if and only if:

$$\frac{1}{D} \frac{1+\alpha}{1+\alpha^{-D}} \sum_{j=1}^{D} \delta_j \alpha^{-j} > \langle \delta \rangle . \tag{S75}$$

Let us now introduce, as above, σ_j such that $\delta_j = \langle \delta \rangle (1 + \sigma_j)$. Recall that $\sum_{j=1}^{D} \sigma_j = 0$. Eq. S75 becomes:

$$\frac{1}{D} \frac{1+\alpha}{1+\alpha^{-D}} \left(\sum_{j=1}^{D} \alpha^{-j} + S(\alpha) \right) > 1 , \quad \text{i.e.}$$

$$S(\alpha) > D \frac{1+\alpha^{-D}}{1+\alpha} - \frac{1-\alpha^{-D}}{\alpha - 1} , \quad \text{i.e.}$$

$$S(\alpha) > \frac{(D-1)\alpha - (D+1) + (D+1)\alpha^{1-D} - (D-1)\alpha^{-D}}{\alpha^2 - 1} \equiv g(\alpha) . \tag{S76}$$

If a heterogeneous line satisfies Eq. S76, it features a higher mutant fixation probability than a homogeneous clique, to first order in st. This means that environment heterogeneity then allows the line to overcome the suppression of selection that the homogeneous line features when compared to the clique.

No amplification is possible if the mutant fitness advantage monotonically decreases left to right and $\alpha < 1$. We showed in the previous section that if the mutant fitness advantage monotonically decreases left to right and $\alpha < 1$, the mutant fixation probability in the heterogeneous line is smaller than in the homogeneous line. Because the homogeneous line itself suppresses selection, we conclude that no amplification is possible if the mutant fitness advantage monotonically decreases left to right and $\alpha < 1$.

Another way to see this, which connects more directly to Figure 3A, is that if $\alpha < 1$, $S(\alpha) < 0$ (see previous section), while we then have $g(\alpha) > 0$ (see below), which entails $S(\alpha) < g(\alpha)$. To show that $g(\alpha) > 0$ if $\alpha < 1$, we notice that the denominator of $g(\alpha)$ is then negative, and thus we aim to show that the numerator is negative too. Denoting the numerator as

$$g_N(\alpha) = (D-1)\alpha - (D+1) + (D+1)\alpha^{1-D} - (D-1)\alpha^{-D}, \tag{S77}$$

its derivative with respect to α reads:

$$g'_N(\alpha) = (D-1)\left[1 + \alpha^{-D}(-D-1+D\alpha^{-1})\right], \tag{S78}$$

and its second derivative with respect to α is:

$$g''_N(\alpha) = D(D-1)(D+1)\,(\alpha-1)\,\alpha^{-D-2}. \tag{S79}$$

When $\alpha < 1$, $g''_N(\alpha) < 0$. Thus, g'_N is a decreasing function on $(0,1)$. As $\lim_{\alpha\to 1^-} g'_N(\alpha) = 0^+$, we have $g'_N(\alpha) > 0$ on $(0,1)$, which entails that g_N is an increasing function on $(0,1)$. As $g_N(1) = 0$, we conclude that $g_N(\alpha) < 0$ on $(0,1)$. Hence, $g(\alpha) > 0$ if $\alpha < 1$ as announced, and there cannot be amplification of selection for $\alpha < 1$.

Step environmental profile with one special upstream deme. Let us consider the case where the mutant is advantaged only in the leftmost deme ($\delta_1 = 1$, $\delta_i = 0$ for $i > 1$). We plot $S(\alpha)$ together with $g(\alpha)$ in such a case in Figure 3A in the main text. This Figure suggests that Eq. S76 is valid for all $\alpha > 1$, and that the difference between $S(\alpha)$ and $g(\alpha)$ becomes smaller and smaller when α increases. To better understand this, let us investigate asymptotics for $\alpha \to \infty$. In the simple case considered here, we have:

$$S(\alpha) = (D-1)\alpha^{-1} - \sum_{i=2}^{D} \alpha^{-i} \sim (D-1)\alpha^{-1} - \alpha^{-2}, \tag{S80}$$

where we have retained the two leading terms in powers of α when $\alpha \to \infty$. Doing the same for $g(\alpha)$, Eq. S76 gives:

$$g(\alpha) \sim (D-1)\alpha^{-1} - (D+1)\alpha^{-2}. \tag{S81}$$

Thus, in this case, $S(\alpha) > g(\alpha)$ in terms of asymptotic behavior for $\alpha \to \infty$. Note however that for other choices of σ_i that monotonically decrease from left to right, the curves representing $S(\alpha)$ and $g(\alpha)$ often cross again for some $\alpha > 1$, meaning that amplification then exists in a finite range of α.

Examples of other environmental profiles. Let us first continue our analysis of the simple step environmental gradient. In Figure 3B in the main text, we considered the case where mutants had an advantage only in the deme upstream of the main migration flow (special deme on the left and $\alpha > 1$). Figure 3A also shows that Eq. S76 does not hold with the same environment profile when $\alpha < 1$. In Figure S7A, we further show the case where the mutant is advantaged in the rightmost deme with $\alpha > 1$ (by symmetry, this is equivalent to having a special leftmost deme with asymmetry $1/\alpha$, giving $\alpha < 1$), and as predicted above, we obtain suppression of selection. This suppression is even stronger than in a homogeneous line. These results show that in the line, when the environmental profile has the same orientation as the overall migration flow, selection can be amplified. Meanwhile, when the environmental profile is homogeneous or opposes the overall migration flow, selection is suppressed.

Figure S7B-C (see also Figure 3C-D) also shows that while the line with similarly-oriented migration flow and environmental profile can accelerate fixation or extinction compared to the homogeneous clique and to the homogeneous line with the same migration asymmetry. Conversely, Figure S7B-C shows that the line with opposite migration flow and environmental profile tends to feature slower fixation or extinction than the homogeneous clique and to the homogeneous line with the same migration asymmetry.

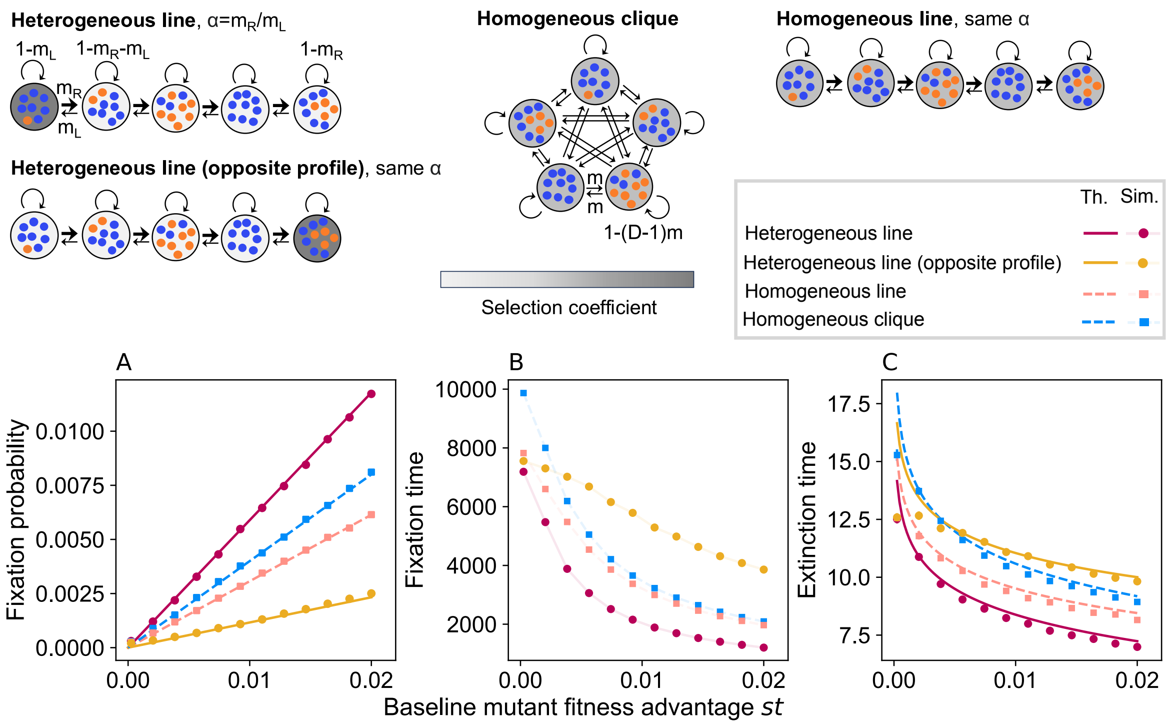

Figure S7: **Impact of the direction of the environmental profile in the line.** Top: Schematic of all spatial structures considered in this Figure: heterogeneous lines with overall migration flow and environmental profile that are either similarly oriented or opposite (left); homogeneous clique (middle), and homogeneous line with the same migration probabilities (right). All spatial structures have the same average mutant fitness advantage (same $\langle\delta\rangle$). Panel A: Mutant fixation probability in the different spatial structures considered, versus baseline mutant fitness advantage st. Panel B: Mutant fixation time, in number of bottlenecks, in the different spatial structures considered, versus baseline mutant fitness advantage st. Panel C: Same as in B, but for mutant extinction time. Results from the branching process theory ("Th.") are shown in panels A (Eq. S62) and C (Eq. S13). Stochastic simulation results ("Sim.") are shown in panels A-C. Parameter values for all structures: $D = 5$, $K = 1000$; for all lines: $\alpha = 1.5$, $m_L = 0.3$; for the clique: $m = 0.15$; for heterogeneous lines: $\delta = 1$ in the special deme, $\delta = 0$ in other ones. Each marker comes from 5×10^5 stochastic simulation realizations.

In Figure S8, we further investigate the effect of different environmental profiles in a line with $\alpha > 1$. We consider a linear and a Gaussian profile, both with the same orientation as the overall migration flow (i.e., the demes where the mutants are most advantaged are upstream). These profiles are intermediate cases between the step environmental profile and the homogeneous environment. We chose them so that the average mutant fitness advantage is the line is the same in all cases (same $\langle\delta\rangle$), see Figure S8A for a depiction of the spatial profiles considered. We observe in Figure S8B that these various cases yield some amplification of selection, and that larger environmental contrast between demes is associated to more amplification of selection. Figure S8C-D further suggest that larger environmental contrast between demes leads to more acceleration of mutant fixation and extinction.

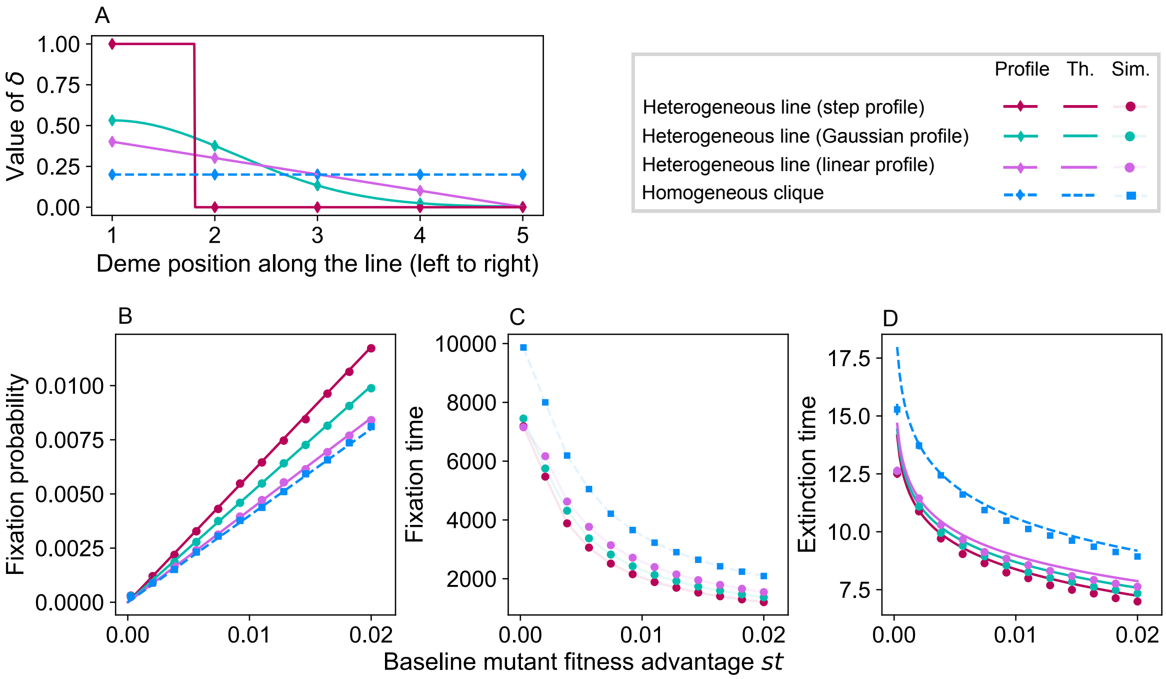

Figure S8: **Impact of different environmental profiles in the line.** Panel A: Different environmental profiles considered: step, Gaussian, linear, and homogeneous. For each of them, the mutant fitness advantage prefactor δ is shown versus position along the line. In what follows, we consider lines with $\alpha = 1.5$ and values δ_i taken in each of these profiles at regular intervals for $i \in \{1, \dots, 5\}$ (markers) for each of the 5 demes of the graph. The profiles are chosen so that all lines have the same $\langle \delta \rangle$. Panel B: Mutant fixation probability in the different structures considered, versus baseline mutant fitness advantage st. Panel C: Mutant fixation time, in number of bottlenecks, in the different structures considered, versus baseline mutant fitness advantage st. Panel D: Same as in C, but for mutant extinction time. Results from the branching process theory ("Th.") are shown in panels B (Eq. S19) and D (Eq. S13). Stochastic simulation results ("Sim.") are shown in panels B-D. Parameter values for all structures: $D = 5$, $K = 1000$; for all lines: $\alpha = 1.5$, $m_L = 0.3$; for the clique: $m = 0.15$. Each marker comes from 5×10^5 stochastic simulation realizations.

4 Spatial structures with one special deme

Our results for the heterogeneous star and of the heterogeneous line show that environment heterogeneity can allow spatially structured populations with frequent asymmetric migrations to amplify natural selection. They also suggest that this happens when the overall migration flow goes from a region where mutants are most beneficial to a region where they are less beneficial. To further explore this, we now consider a more general spatial structure, where all pairs of demes are connected by migrations, but where one deme has a migration outflow that differs from others, and can thus send out more individuals. We assess the impact of having a different environment in that special deme.

First, in Section 4.1, we consider the simplest version of this structure with a special deme where the environment and the outflow both differ from those of other demes, assuming a highly symmetric migration matrix. We determine mutant fixation probability to first order in st, and obtain a condition on the asymmetry in migrations and the environment heterogeneity to obtain amplification of selection. Next, in Section 4.2, we consider more general and less symmetric migration matrices, with coefficients drawn in Dirichlet distributions (see Ref. [1] for the homogeneous environment case), with one special deme where both the environment and the migration outflow differ from those of other demes.

4.1 Highly symmetric structure with one special deme

4.1.1 Fixation probability

Let us consider a simple structure where the first deme has a different migration outflow, i.e. it sends out more (or fewer) individuals than others. Specifically, we assume that all outgoing migrations from deme 1 happen with probability m_1, while all other migrations across the structure occur with probability m_2. Figure 4 illustrates the case $m_2 < m_1$. In addition to having a different outflow, the first deme is such that the relative mutant fitness advantage is $\delta_1 s$, while in all other demes it is $\delta_2 s$. Under these assumptions, only the first deme is different from others, and all other ones are equivalent to each other: this structure is highly symmetric.

Since $\sum_{k=1}^{D} m_{kj} = 1$ for all j, we have the following constraint on migration probabilities:

$$m_1 + (D-1)m_2 = 1\,. \tag{S82}$$

The first line of Eq. S8, written for the first node and for another one, yields:

$$a_1 = m_1 a_1 + (D-1)m_1 a_2\,, \tag{S83}$$
$$a_2 = m_2 a_1 + (D-1)m_2 a_2\,, \tag{S84}$$

Note that we have used the symmetry of the structure to write that $a_i = a_2$ for all $i \geq 2$. Using Eqs. S82, both Eq. S83 and Eq. S84 reduce to:

$$a_2 = \frac{a_1}{\tilde{\alpha}}\,, \quad \text{with} \quad \tilde{\alpha} = \frac{m_1}{m_2}\,. \tag{S85}$$

Note that here we introduced $\tilde{\alpha} = m_1/m_2$ while the most analogous quantity to our work with the star (here and in Refs. [1, 6]) would be $\alpha = m_2/m_1 = \tilde{\alpha}^{-1}$. This is motivated by the fact that our results in the next Sections take a simpler form with $\tilde{\alpha}$. However, to facilitate the comparison with the star, we express fixation probabilities as a function of $\tilde{\alpha}^{-1}$.

The second line of Eq. S8, written for the first node and for another one, yields:

$$b_1 = m_1 b_1 + (D-1)m_1 b_2 + a_1^2 - 2a_1\delta_1\,, \tag{S86}$$
$$b_2 = m_2 b_1 + (D-1)m_2 b_2 + a_2^2 - 2a_2\delta_2\,, \tag{S87}$$

Summing Eq. S86 with $(D-1)$ times Eq. S87, and using Eq. S82, we obtain:

$$a_1^2 + (D-1)a_2^2 - 2a_1\delta_1 + 2(D-1)a_2\delta_2 = 0\,, \tag{S88}$$

which yields

$$a_1 = 2\frac{\delta_1 + (D-1)\delta_2\tilde{\alpha}^{-1}}{1 + (D-1)\tilde{\alpha}^{-2}}\,. \tag{S89}$$

Eqs. S85 and S89 then provide the fixation probability ρ, to first order in st, of a mutant appearing at a bottleneck in a deme chosen uniformly at random in the structure:

$$\rho = st\,\frac{a_1 + (D-1)a_2}{D} = 2st\,\frac{[1 + \tilde{\alpha}^{-1}(D-1)][\delta_1 + (D-1)\delta_2\tilde{\alpha}^{-1}]}{D\,[1 + (D-1)\tilde{\alpha}^{-2}]}. \tag{S90}$$

Note that, for $\tilde{\alpha} = 1$, this reduces to $\rho = 2\langle\delta\rangle st$, as in all other structures considered so far.

4.1.2 Condition for amplification of selection

The fixation probabilities in our structure and in a clique are given respectively in Eqs. S90 and S19 to first order in st. The first-order fixation probability is larger in our structure than in a clique with the same average relative mutant fitness advantage if and only if

$$\begin{aligned}\frac{[1 + \tilde{\alpha}^{-1}(D-1)][\delta_1 + (D-1)\delta_2\tilde{\alpha}^{-1}]}{1 + (D-1)\tilde{\alpha}^{-2}} &> \delta_1 + (D-1)\delta_2\,, \quad \text{i.e.} \\ \delta_1\tilde{\alpha}^{-1}\left(1 - \tilde{\alpha}^{-1}\right) &> \delta_2\left(1 - \tilde{\alpha}^{-1}\right)\,.\end{aligned} \tag{S91}$$

Let us introduce

$$\beta = \frac{\delta_1}{\delta_2}\,, \tag{S92}$$

which characterizes environment heterogeneity in this structure. If $\tilde{\alpha} > 1$, i.e. if the special deme has a stronger outflow than others, Eq. S91 reduces to:

$$\beta > \tilde{\alpha}\,. \tag{S93}$$

Since here $\tilde{\alpha} > 1$, we find that the mutant should have a sufficiently stronger advantage in the special deme than in the other demes for the structure to amplify selection (δ_1 should be sufficiently larger than δ_2).

If $\tilde{\alpha} < 1$, i.e. if the special deme has a weaker outflow than others, Eq. S91 reduces to:

$$\beta < \tilde{\alpha}\,. \tag{S94}$$

Since here $\tilde{\alpha} < 1$, we find that the mutant should have a sufficiently stronger advantage in the other demes than in the special deme for the structure to amplify selection (δ_2 should be sufficiently larger than δ_1).

These results generalize our previous observations in the star and line that environment heterogeneity can allow spatially structured populations with frequent asymmetric migrations to amplify natural selection when the overall migration flow goes from a region where mutants are most beneficial to a region where they are less beneficial.

4.2 More general structures with one special deme

Let us now consider more general structures with one special deme. Specifically, let us consider Dirichlet cliques with one special deme. Dirichlet cliques are spatial structures with tunable migration asymmetries on each edge of a clique, which were introduced, and studied with a homogeneous environment, in Ref. [1]. They have migration probabilities $(m_{1j}, m_{2j}, \ldots, m_{Dj})$ sampled from a Dirichlet distribution with parameters $\eta_1, \ldots, \eta_D$ for each j. The Dirichlet distribution has probability distribution function:

$$\Phi_{(\eta_1,\ldots,\eta_D)}(x_1, \ldots, x_D) = \frac{1}{B(\eta_1, \ldots, \eta_D)} \prod_{i=1}^{D} x_i^{\eta_i - 1}, \tag{S95}$$

with $B(\eta_1, \ldots, \eta_D) = \prod_{i=1}^{D} \Gamma(\eta_k)/\Gamma(\sum_{i=1}^{D} \eta_k)$ and where Γ denotes the gamma function. Random variables $(X_1, \ldots, X_D)$ sampled from this distribution have respective expectation values $\mathbb{E}(X_i) = \eta_i / \sum_j \eta_j$. Considering $(\eta_1, \eta_2, \ldots, \eta_D) = (\eta, 1, \ldots, 1)$ allows us to make deme 1 special in terms of migration outflow. In fact, the expectation values are such that $\mathbb{E}(m_{1i})/\mathbb{E}(m_{i1}) = \eta$ for all i, which entails that, ignoring the variability of these migration probabilities, η maps to $\tilde{\alpha} = m_1/m_2$ in the highly-symmetric structure considered in the previous sections.

Construction of Figure 4. In Figure 4, we consider values of η both greater than and less than 1, in order to produce cases with either strong outflow or weak outflow from deme 1. For each value of η, we generate several (in practice, 5×10^3) Dirichlet cliques, and we associate all of them to $\tilde{\alpha} = \eta$. Note that despite this mapping to the highly-symmetric structure considered above, the migration probabilities are variable across various edges in a given Dirichlet clique, and across different Dirichlet cliques. As an illustration, Figure S9 shows example histograms for two different values of η of m_{1i} for all i, with expectation value m_1, and of m_{ij} for all $i \neq 1$ and all j, with expectation value m_2.

In Figure 4, we also vary environment heterogeneity, via the ratio $\beta = \delta_1/\delta_2$, while keeping fixed the average mutant fitness advantage $\langle\delta\rangle$ in each of the structures we consider. This leads to:

$$\delta_2 = \frac{D\langle\delta\rangle}{\beta + D - 1}\,, \qquad \text{and} \qquad \delta_1 = \beta\delta_2\,. \tag{S96}$$

We finally use the general method presented in Section 1.3 to obtain mutant fixation probability to first order in st for the Dirichlet cliques associated to a given $\tilde{\alpha}$. Comparing to the first-order mutant fixation probability for a standard clique then gives the regions of amplification of selection shown in Figure 4.

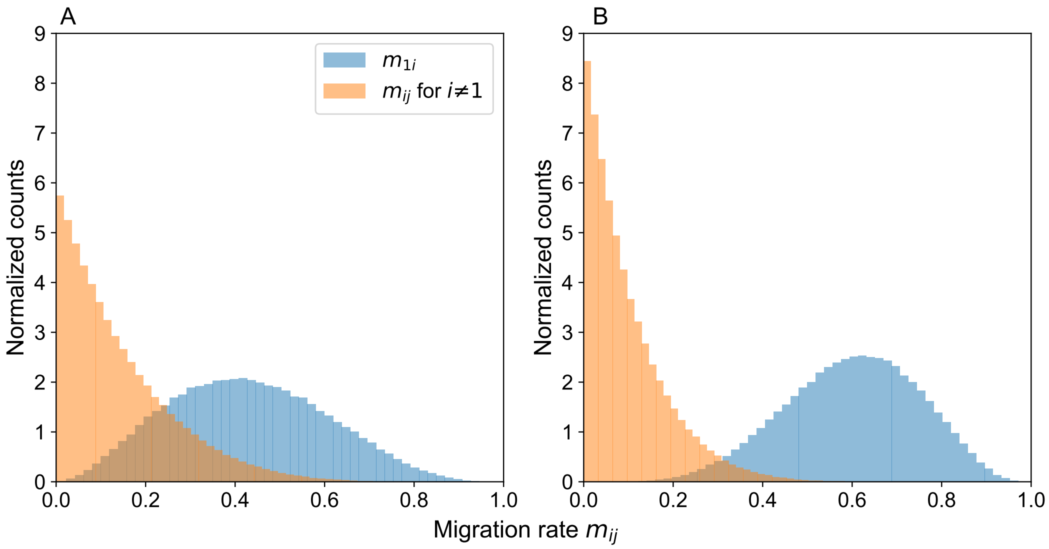

Figure S9: **Distribution of migration probabilities in Dirichlet cliques with one special deme.** Histograms of m_{1i} for all i, whose expectation value is m_1, are shown in blue, and histograms of m_{ij} for all $i \neq 1$ and all j, whose expectation value is m_2, are shown in orange. Parameter values: Panel A: $\eta = 3$, yielding $m_1 = 0.43$, $m_2 = 0.14$ and $\tilde{\alpha} = 3$; Panel B: $\eta = 6$, yielding $m_1 = 0.60$, $m_2 = 0.10$ and $\tilde{\alpha} = 6$. For each panel, 5×10^4 Dirichlet clique migration matrices were generated.

5 Deleterious mutants

We demonstrated that environment heterogeneity can induce amplification of selection in deme-structured populations on graphs, under frequent migrations. The branching process calculations that we performed so far hold under the conditions $s > 0$, $K \gg 1$ and $1/K \ll st \ll 1$ [1]. In particular, under the branching process approximation, the extinction of deleterious mutants ($s < 0$) is certain [1]. However, deleterious mutants have a nonzero fixation probability in finite-size populations due to genetic drift, which can be calculated under the diffusion approximation in well-mixed populations [8]. Does the amplification of selection we evidenced extend to deleterious mutations? The complete definition of amplification of selection by spatial structure is that it increases the fixation probability of beneficial mutants but decreases that of deleterious mutants, compared to a well-mixed population [5]. Here, we explore this point by using numerical simulations.

In Figure S10, we consider three different spatial structures where we demonstrated amplification of selection for $s > 0$, and we report the fixation probability for a range of selection coefficients that includes $s < 0$, obtained from stochastic simulations. In all cases where beneficial mutants have an increased fixation probability compared to the homogeneous clique with the same $\langle \delta \rangle$, we also observe that deleterious mutants have a decreased fixation probability compared to the homogeneous clique with the same $\langle \delta \rangle$. This suggests that the amplification of selection we evidenced extends to $s < 0$.

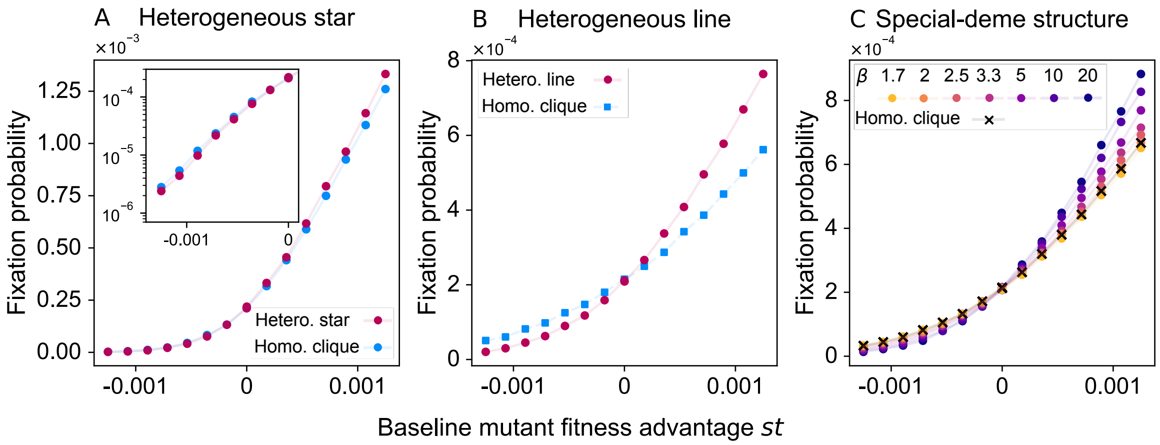

Figure S10: **Amplification of selection also holds for deleterious mutants.** Mutant fixation probability versus baseline mutant fitness advantage st over a range of st centered around zero, in different spatial structures where we observed amplification for $s > 0$. In all cases, the homogeneous clique with the same $\langle\delta\rangle$ is shown for reference. Panel A: heterogeneous star. The inset shows a magnification of the negative st region, with logarithmic vertical axis. Parameter values: as in Figure S5, $\alpha = 2$, $m_O = 0.1$, $\delta_C = 0.05$ in the center and $\delta_L = 0.625$ in all leaves, giving $\langle\delta\rangle = 0.5$. Panel B: heterogeneous line. Parameter values: as in Figure 3, $\alpha = 1.5$, $m_L = 0.3$, $\delta = 1$ in the leftmost deme and $\delta = 0$ in all others, resulting in $\langle\delta\rangle = 0.2$. Panel C: structure with one special deme for several values of environment heterogeneity $\beta = \delta_1/\delta_2$. Parameter values: as in Figure 4, $\tilde{\alpha} = 2$ and $\langle\delta\rangle = 0.25$. Recall that for $s > 0$, in the branching process regime, we expect amplification for $\beta > \tilde{\alpha} = 2$ (see Section 4.1). Parameter values common to all panels: $K = 1000$, $D = 5$, and $m = 0.15$ for the clique. Each marker comes from 5×10^6 stochastic simulation realizations (except for the first three values of st in panel A, for which 5×10^7 realizations were used).

6 Impact of the number of demes on the fixation probability

In this Section, we analyze the impact of varying the number D of demes on the fixation probability of a mutant in a spatially structured population on graphs with heterogeneous environments, in the branching process regime. We consider the spatial structures discussed above, namely the star, the line and highly-symmetric structures with one special deme. In all cases, we compare results to those obtained for a clique with a homogeneous environment that has the same number of demes, the same deme size and the same average mutant advantage (i.e., in practice, the same $\langle\delta\rangle$).

Note that, in the branching process approximation, we assume $K \gg 1$, and K does not enter in our expressions of the mutant fixation probability. Hence, we do not discuss further the impact of K here, and we vary D while assuming $K \gg 1$.

6.1 Star

The fixation probability of a mutant in a heterogeneous star graph is given by Eq. S52 to first order in st, considering a single mutant introduced at a bottleneck in a deme chosen uniformly at random. Let us now study the impact of D on this fixation probability. For this, we focus on the case where all leaves have the same environment, but the center may have a different one, i.e., $\delta_i = \delta_L$ for all $i \in \mathcal{L}$, but we may have $\delta_C \neq \delta_L$. We vary D while keeping $\langle\delta\rangle$ constant, as well as the contrast $C = (\delta_C - \delta_L)/\langle\delta\rangle$ between the center and the leaf environment. The fixation probability of a mutant in a heterogeneous star graph then reads

$$\begin{aligned}\rho &= 2st\,\frac{[1+\alpha(D-1)]\,[\delta_C+(D-1)\alpha\delta_L]}{D[1+\alpha^2(D-1)]}\\ &= 2\langle\delta\rangle st\,\frac{1+\alpha(D-1)}{D^2[1+\alpha^2(D-1)]}\,[CD+D-C+\alpha(D-1)(D-C)]\ ,\end{aligned} \tag{S97}$$

where we used $\delta_C = (CD+D-C)\langle\delta\rangle/D$ and $\delta_L = (D-C)\langle\delta\rangle/D$.

Large-D limit. For large D, Eq. S97 yields the following expansion:

$$\rho = 2\langle\delta\rangle st \left[1 + \frac{1}{D}\left(\frac{1}{\alpha} - 1\right)\left(1 - \frac{1}{\alpha} + C\right) + O\left(\frac{1}{D^2}\right)\right]. \tag{S98}$$

This fixation probability tends to that in the clique, $\rho = 2\langle\delta\rangle st$, see Eq. S19, when $D \to \infty$. Furthermore, in the large-D limit, the fixation probability of a mutant is larger in the heterogeneous star than in the clique if and only if

$$\left(\frac{1}{\alpha} - 1\right)\left(1 - \frac{1}{\alpha} + C\right) > 0. \tag{S99}$$

This condition is satisfied for the values of α and C considered in Figure S5.

Figure S11A shows the first-order coefficient in the expansion of the fixation probability in st, given by Eq. S97, versus D, in a star with other parameter values matching those of Figure S5. We observe that amplification persists but weakens when D is increased for this heterogeneous star, while suppression persists and weakens for the corresponding homogeneous star. These behaviors are well-predicted by our asymptotic expansion for large D from Eq. S98.

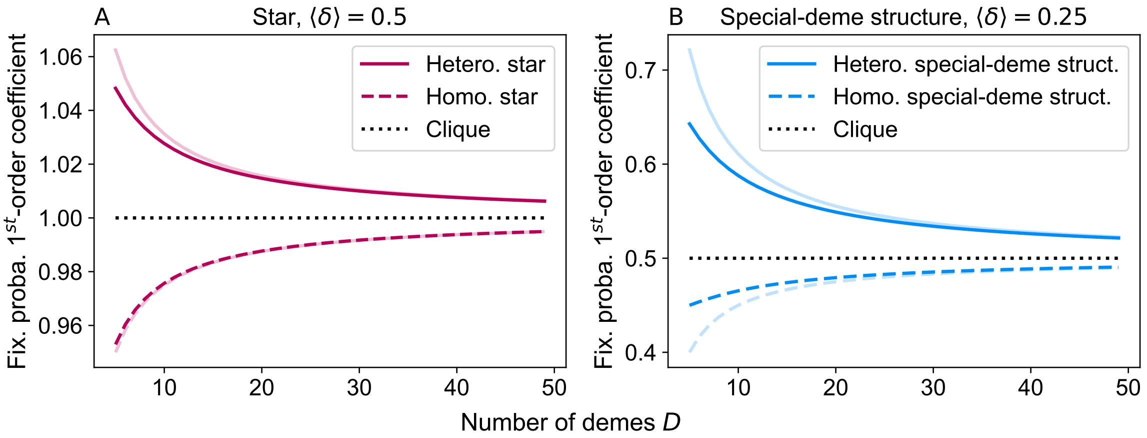

Figure S11: **Impact of the number of demes on the fixation probability in the star and in the structure with one special deme.** Panel A: First-order coefficient in the expansion of the fixation probability in st, given in Eq. S97, versus the number of demes D, for a star structure. Solid lines: heterogeneous environment; dashed lines: homogeneous environment; pale lines: corresponding large-D asymptotic predictions from Eq. S98. The case of the clique with the same $\langle\delta\rangle$ is shown for reference. Parameter values: as in Figure S5, $\langle\delta\rangle = 0.5$, $\alpha = 2$, and $C = -1.125$. Panel B: Same as in A, but for a highly symmetric structure with one special deme. First-order coefficient in st in Eq. S97 with the substitution $\alpha \to \tilde{\alpha}^{-1}$ and large-D asymptotic predictions from Eq. S98 are shown for heterogeneous and homogeneous environments. In both panels, the average mutant fitness advantage prefactor $\langle\delta\rangle$ and the contrast C are held fixed while varying D. Parameter values: as in Figure 4, $\langle\delta\rangle = 0.25$, $\tilde{\alpha} = 2$, and $C = 3.214$ (corresponding to $\beta = 10$).

6.2 Highly-symmetric structure with one special deme

The fixation probability of a mutant in a highly-symmetric structure with one special deme is given by Eq. S90 to first order in st, considering a single mutant introduced at a bottleneck in a deme chosen uniformly at random. With the substitution $\alpha \to \tilde{\alpha}^{-1}$ (see Section 4.1), this is the same as the fixation probability of a mutant in a heterogeneous star graph in the particular case where all leaves have the same environment, which we considered in Section 6.1, see Eq. S97. Hence, the conclusions we obtained there hold in this case too. In particular, Eq. S99 gives the condition for the probability of fixation to be larger than in the clique in the large-D limit, namely:

$$(\tilde{\alpha} - 1)(1 - \tilde{\alpha} + C) > 0. \tag{S100}$$

Figure S11B shows the first-order coefficient in the expansion of the fixation probability in st, given by Eq. S97 with $\alpha \to \tilde{\alpha}^{-1}$, versus D. Other parameter values match those of Figure 4, with $\beta = 10 > 2 = \tilde{\alpha}$. As for the star (see Figure S11A), we observe that amplification persists but weakens when D is increased for this heterogeneous structure, while suppression persists and weakens for the corresponding homogeneous structure.

6.3 Line

The fixation probability of a mutant in a heterogeneous line graph is given by Eq. S62 to first order in st, considering a single mutant introduced at a bottleneck in a deme chosen uniformly at random. As done for the star in the previous paragraph, we will now study the impact of D on this fixation probability. We focus on the case where all demes have the same environment, except the most upstream deme (with respect to the migration flow direction), labeled deme 1 under the assumption that $\alpha > 1$, which may have a different environment, i.e., $\delta_i = \delta_2$ for all $i > 1$, but we may have $\delta_1 \neq \delta_2$. As for the star, we vary D while keeping $\langle\delta\rangle$ constant. However, at first, we do not make the hypothesis that the environment contrast is constant. The fixation probability of a mutant in a heterogeneous line graph then reads

$$\rho = \frac{2st}{D}\frac{1+\alpha}{1+\alpha^{-D}}\left(\delta_1\alpha^{-1} + \delta_2\sum_{j=2}^{D}\alpha^{-j}\right) = \frac{2st}{\alpha D}\frac{1+\alpha}{1+\alpha^{-D}}\left(\delta_1 + \delta_2\frac{1-\alpha^{1-D}}{\alpha-1}\right). \tag{S101}$$

Large-D limit. Since our focus is on $\alpha > 1$, we obtain the following leading order term for $D \gg 1$:

$$\rho \sim 2st\frac{1+\alpha}{\alpha D}\left(\delta_1 + \frac{\delta_2}{\alpha-1}\right). \tag{S102}$$

Meanwhile, in the clique with the same $\langle\delta\rangle = [\delta_1 + (D-1)\delta_2]/D$, the fixation probability reads $\rho = 2st[\delta_1 + (D-1)\delta_2]/D$, see Eq. S19. Hence, in the large-D limit, the fixation probability of a mutant is larger in the heterogeneous line than in the clique if and only if

$$\delta_1 > \alpha D\delta_2\,. \tag{S103}$$

Convention 1: fixed average environment and contrast. Let us now keep the contrast $C = (\delta_1 - \delta_2)/\langle\delta\rangle$ between the deme 1 and others constant, in addition to $\langle\delta\rangle$, similarly to what we did for the star and the structure with one special deme. Eq. S101 then gives

$$\rho = 2\langle\delta\rangle st\frac{1+\alpha}{D\,(1+\alpha^{-D})}\left(\frac{C}{\alpha} + \frac{D-C}{D}\frac{1-\alpha^{-D}}{\alpha-1}\right), \tag{S104}$$

where we used $\delta_1 = (CD + D - C)\langle\delta\rangle/D$ and $\delta_2 = (D-C)\langle\delta\rangle/D$.

To leading order for $D \gg 1$, this yields

$$\rho \sim 2\langle\delta\rangle st\frac{1+\alpha}{D}\frac{C\alpha - C + \alpha}{\alpha(\alpha-1)}, \tag{S105}$$

which tends to 0 as $D \to \infty$, and hence becomes smaller than the fixation probability in the clique. Consistently, we note that in this case, Eq. S103 is not satisfied in the large-D limit. Hence, in this convention, the mutant fixation probability in the heterogeneous line becomes smaller than in the clique for large D, even though it can be larger for small D, as in Figure 3.

Figure S12A shows the first-order coefficient in the expansion of the fixation probability in st, given by Eq. S104, when varying D under convention 1, in a line with other parameter values matching those of Figure 3. We observe that amplification exists for $D \leq 10$, but weakens when D is increased for this heterogeneous line, and turns into suppression for $D > 10$. Meanwhile, suppression increases with D for the corresponding homogeneous line. Besides, the observed trends are well-predicted by our asymptotic expansion for large D from Eq. S105.

Convention 2: fixed average environment and $\delta_2/\delta_1 = 1/D^2$. Let us now set $\delta_2/\delta_1 = 1/D^2$, in addition to keeping $\langle\delta\rangle$ constant. Eq. S101 then gives

$$\rho = 2\langle\delta\rangle st \frac{1+\alpha}{\alpha\left(1+\alpha^{-D}\right)\left(D^2+D-1\right)}\left(D^2 + \frac{1-\alpha^{1-D}}{\alpha-1}\right), \tag{S106}$$

where we used $\delta_1 = D^3\langle\delta\rangle/(D^2+D-1)$ and $\delta_2 = D\langle\delta\rangle/(D^2+D-1)$.

To leading order for $D \gg 1$, this yields

$$\rho \sim 2\langle\delta\rangle st \frac{1+\alpha}{\alpha}, \tag{S107}$$

which is larger than the fixation probability in the clique $\rho = 2\langle\delta\rangle st$. Consistently, we note that in this case, Eq. S103 is satisfied in the large-D limit. Hence, in this convention, the mutant fixation probability in the heterogeneous line is larger than in the clique even for large D.

Figure S12B shows the first-order coefficient in the expansion of the fixation probability in st, given by Eq. S106, when varying D under convention 2, in a line with other parameter values matching those of Figure 3. We observe that amplification increases with D for this heterogeneous line, in contrast with the results from convention 1 (see Figure S12A). The large-D limit is well-predicted by Eq. S107. The case of the corresponding homogeneous line is unchanged, and suppression increases with D. Thus, the discrepancy between the homogeneous and the heterogeneous lines in terms of fixation probability increases with D.

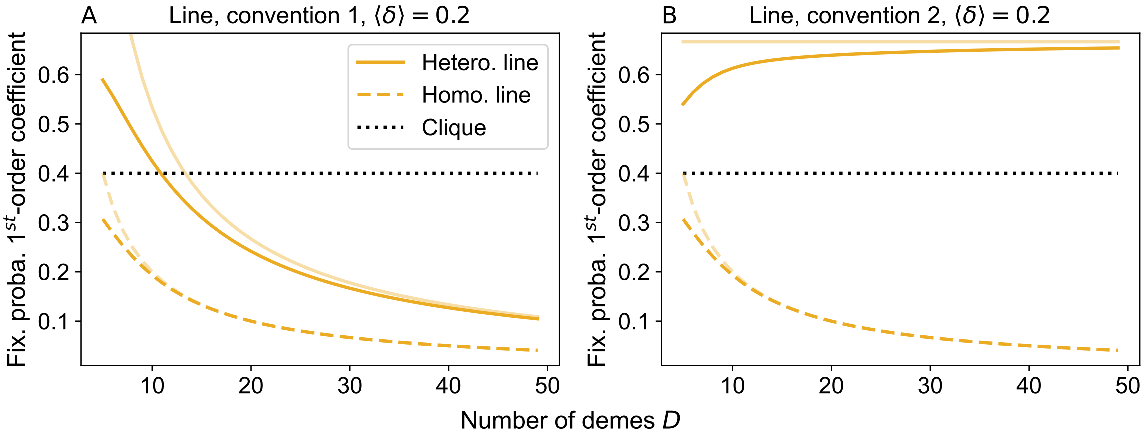

Figure S12: **Impact of the number of demes on the fixation probability in the line.** First-order coefficient in the expansion of the fixation probability in st, versus the number of demes D, for a line structure, shown under two conventions that specify how the environment changes with D. Panel A uses Eq. S104, derived under "convention 1", where the average mutant fitness advantage prefactor $\langle\delta\rangle$ and the contrast C are fixed while varying D. Solid lines: heterogeneous environment; dashed lines: homogeneous environment; pale lines: corresponding large-D asymptotic predictions from Eq. S105. The case of the clique with the same $\langle\delta\rangle$ is shown for reference. Panel B uses Eq. S106 for the heterogeneous line. This result is derived under "convention 2", where the average mutant fitness advantage prefactor $\langle\delta\rangle$ is fixed and the ratio δ_1/δ_2 is set to $1/D^2$ while varying D. The large-D asymptotic prediction for the heterogeneous line are from Eq. S107. Line styles are as in panel A, and the homogeneous line case is the same as in panel A. Parameter values: as in Figure 3, $\langle\delta\rangle = 0.2$, $\alpha = 1.5$ for both panels, $C = 5$ for panel A.

7 More general graph structures

So far, we mainly focused on graphs with strong symmetries or simple structures, namely circulations, the star, the line and the fully-connected structure with one special deme. For frequent migrations, in the branching process regime, we showed that the star, the line and the structure with one special deme can all amplify selection under some environment heterogeneities, while they are suppressors of selection when the environment is homogeneous. We found that this happened when there is an overall migration flow directed from a deme where mutants are strongly advantaged toward other demes where they are less advantaged. How general is this finding? To address this question, we consider all connected graphs with five demes, in the spirit of evolutionary graph theory works [9–11]. There are 21 distinct such graphs, shown in Figure S13. We compute the fixation probability in these graphs to first order in st under the branching process approximation, using our general method from Section 1.3. Importantly, there is an infinity of ways to set the migration probabilities m_{ij} and the mutant fitness advantage prefactors δ_i in these structures. We consider three different conventions setting migration probabilities and mutant fitness advantage prefactors. They are defined below.

Convention 1. We set migration probabilities m_{ij} in order to introduce a directional flow across the structure. We first number nodes as follows. Node number 1 is the node with smallest degree. If several nodes have that same degree, we select the node whose neighbors' degrees have the smallest sum, and if there is still ambiguity, node number 1 is selected uniformly at random among the possible ones. Next, we choose node number 2 as the neighbor of node number 1 that has the smallest degree (and if there is ambiguity, we use the same strategy as for node 1). We repeat this procedure until all nodes are labeled. We then introduce a forward migration probability m_F, and a backward migration probability m_B, such that $\alpha = m_F/m_B > 1$. For each pair of nodes $(i, j \neq i)$ that is connected in the graph of interest, we set all non-zero elements of the adjacency matrix in the upper-right triangle $(i < j)$ to $m_{ij} = m_F$ if $i < j$ and $m_{ij} = m_B$ if $i > j$. Finally, we set $m_{ii} = 1 - \sum_{j \neq i} m_{ji}$.

We define the mutant fitness advantage prefactor δ_i in each deme i to be proportional to the inverse degree of the corresponding node, multiplied by a decreasing sequence of the node index, chosen as $\{2^{-i}\}_{i=1}^{5}$. We further normalize the values so that the average mutant fitness advantage prefactor $\langle \delta \rangle$ is the same across all structures.

This procedure ensures that the overall migration flow is directed from the demes where the mutant has the strongest fitness advantage to those where this advantage is smaller.

Convention 2. We set $m_{ii} = 1/5$ for all i and $m_{ij} = 4/(5d_j)$ for each pair of nodes $(i, j \neq i)$ that is connected in the graph of interest, with d_j the degree of node j. This ensures that $\sum_i m_{ij} = 1$.

We define the mutant fitness advantage prefactor in each deme to be proportional to the degree of the corresponding node. Values are normalized so that the average mutant fitness advantage prefactor $\langle \delta \rangle$ is the same across all structures.

Convention 3. Migration probabilities are the same as in convention 2.

We define the mutant fitness advantage prefactor in each deme to be proportional to the inverse degree of the corresponding node. Again, values are normalized so that the average mutant fitness advantage prefactor $\langle \delta \rangle$ is the same across all structures.

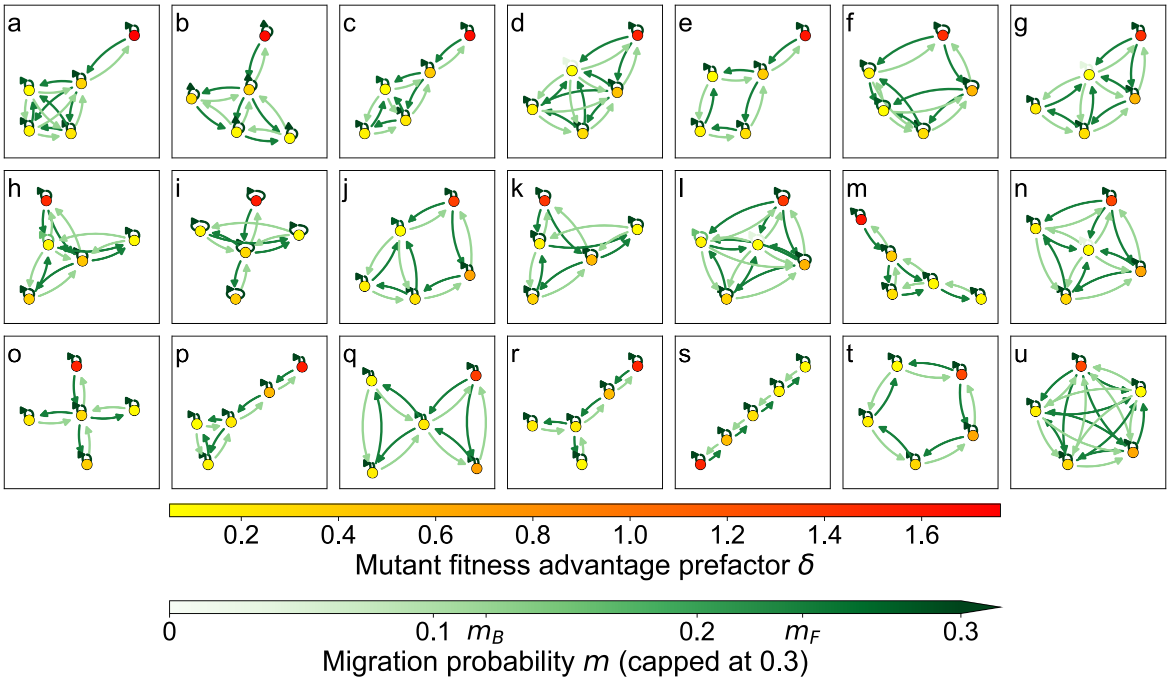

Figure S13: **Visualization of all distinct connected graphs with five nodes under convention 1.** Each lowercase letter (a–u) labels a distinct graph. Mutant fitness advantage prefactors and migration probabilities are those from convention 1 (see text), where the overall migration flow is directed from the demes where the mutant has the strongest fitness advantage to those where this advantage is smaller. Here, $\langle\delta\rangle = 0.5$, $m_B = 0.12$, $\alpha = m_F/m_B = 2$.

Figure S14 shows the first-order coefficient in the expansion of the fixation probability in st under the three different conventions defined above. Under convention 1, we observe that amplification of selection exists in all graphs considered, except the cycle and the clique, which have no effect (labeled t and u respectively, see Figure S13). Consistently, we showed above that, to first order in st, there is no amplification or suppression of selection in the clique and in the cycle, which are both circulations (see Sections 2.1.2 and 2.1.2). Conversely, under convention 2 and 3, we observe suppression of selection in all graphs except the cycle and the clique, and we note that this suppression tends to be stronger under convention 3 than under convention 2.

Under convention 1, the overall migration flow is directed from the demes where the mutant has the strongest fitness advantage to those where this advantage is smaller. Our results suggest that this can generically lead to amplification of selection. We further observe that this amplification of selection is stronger when the deme where mutants are most advantaged has degree 1, while other nodes are strongly connected together (graphs a, b, c, e), and next, similar graphs but where the deme that most advantages mutants has degree two (graphs d, f, g, h). Besides, the Pearson correlation between $\mathrm{Var}(\delta)$ and the first-order coefficient of the fixation probability is 0.71, illustrating the importance of environment heterogeneity for amplification. Note that a variant of convention 1, where migration probabilities are the same but the mutant fitness advantage prefactor in deme i is set to 2^{-i} also leads to amplification of selection, albeit a bit more weakly (mean first-order coefficient 1.10 versus 1.17 under convention 1). Nevertheless, the results obtained under conventions 2 and 3 suggest that suppression of selection remains the most generic effect of spatial structure under frequent migrations in the branching process regime, as for homogeneous environments [1]. Specific situations, such as the overall migration flow going in the same direction as the environment gradient, are required to inverse that trend. Overall, these results show that our conclusions obtained with specific graphs extend to others.

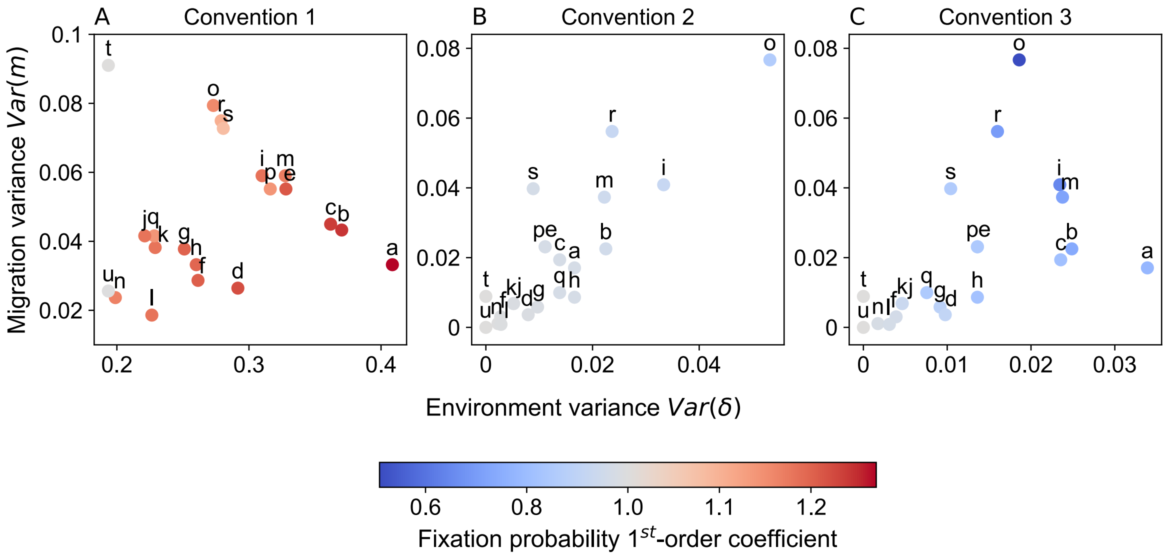

Figure S14: **Fixation probability in all distinct connected graphs with 5 nodes, under 3 different conventions of migration and environment heterogeneities.** In each panel, each marker corresponds to one of the 21 distinct connected graphs with 5 nodes shown in Figure S13. Its color indicates the value of the first-order coefficient in the expansion of the fixation probability in st, computed numerically as described in Section 1.3, and is labeled using the same lowercase letter as in Figure S13. Here, $\langle\delta\rangle = 0.5$, which entails that there is amplification of selection if the first-order coefficient is larger than $2\langle\delta\rangle = 1$ (red) and suppression of selection if it is smaller than that (blue). Results are plotted versus the variance $\mathrm{Var}(m)$ of nonzero migration probabilities and the variance $\mathrm{Var}(\delta)$ of the mutant fitness advantage prefactor across demes in the graph considered. Each panel corresponds to a different convention for defining migration probabilities and mutant fitness advantage prefactors in each graph, as defined in the text (in particular, panel A corresponds to convention 1, which is represented in Figure S13).

8 Rare migration regime

In this Section, we turn to the rare migration regime, that is to the case where fixation or extinction of a type within a deme happens on faster timescales than any migrations between demes. Recall that so far, all analytical calculations were made assuming frequent migrations, in the branching process framework. However, with rare migrations, another approach allows to obtain analytical results. Indeed, given the separation of timescales that exists in this regime, one can consider that each deme is either fully mutant or fully wild-type when migrations happen. One can then study mutant fixation using a Markov chain in a coarse-grained description, where migrations can result into a change of state of demes from fully mutant to fully wild-type, and vice-versa [6, 12]. Below, we consider the star and the line with rare migrations. We calculate their mutant fixation probabilities for heterogeneous environments, and we show that amplification of selection can be observed in this regime. Importantly, in this regime, we can address deleterious mutants as well as beneficial ones, and consider a broad range of fitness differences, while the branching process approach is restricted to beneficial mutants ($s > 0$) satisfying $st \ll 1$ and $st \gg 1/K$.

8.1 Star with heterogeneous environment

8.1.1 Mutant fixation probability

Let us consider a star with D demes, where migrations from each leaf to the center (resp. from the center to each leaf) occur with a rate per individual m_I (resp. m_O). In the rare migration regime, the state of the system can be fully described by two numbers: (1) a binary number indicating whether the center is wild-type or mutant, and (2) the number i of mutant leaves.

Below, we determine the probability of fixation of a mutant in the star in the rare migration regime, starting from a fully mutant deme chosen uniformly at random. For this, we generalize the calculation of Ref. [6], which assumed the same environment in each deme (see the Supplementary Material of that paper), to the case of heterogeneous environments. We focus on the case where the center has a different environment from the leaves. We assume that all leaves have the same environment, thereby preserving the symmetry of the star, where all leaves are equivalent. Thus, the key difference compared to the homogeneous case [6] is that here, the probability of fixation of a given type (mutant or wild-type) in the center is different from that in a leaf. In practice, here, for simplicity, we will assume that this arises from the fact that the mutant has a different fitness advantage in the center and in leaves.

Note that, compared to Ref. [6], we do not take into account the fact that the equilibrium deme size (or bottleneck size) of a deme can differ depending on whether the deme is mutant or wild type. Indeed, here, we consider serial dilution models, which are similar to Wright-Fisher models, and where this bottleneck size is set by a regulation, assumed to be independent from composition. (In models with individual-level birth and death probabilities and logistic regulation, such as that of Ref. [6], the steady-state deme size depends on fitness, and thus differs between mutants and wild-types, but this difference is small when fitnesses f satisfy $f \gg g$, where g represents death rate. The calculation below can easily be extended to take this difference into account if necessary [6].)

Upon a given migration event, the probability that the mutant type fixes in the center, if the center is initially wild-type and i leaves are mutant, reads

$$T_{(0,i)\to(1,i)} = \frac{m_I i}{m_I i + m_I(D-1-i) + m_O N_W (D-1)} \rho_M^C \,, \tag{S108}$$

where ρ_M^C is the probability of fixation of one mutant in a wild-type center. Indeed, this scenario happens if there is a migration from a mutant leaf to the center, and the mutant then fixes in the center. Similarly, the probability that the wild-type fixes in the center, if the center is initially mutant and i leaves are mutant, reads

$$T_{(1,i)\to(0,i)} = \frac{m_I(D-1-i)}{m_I i + m_I(D-1-i) + m_O(D-1)} \rho_W^C \,, \tag{S109}$$

where ρ_W^C is the probability of fixation of one wild-type individual in a mutant center. Then, the probability that the number of mutant leaves increases by 1 if the center is mutant is

$$T_{(1,i)\to(1,i+1)} = \frac{m_O(D-1-i)}{m_I i + m_I(D-1-i) + m_O(D-1)} \rho_M^L \,, \tag{S110}$$

where ρ_M^L is the probability of fixation of one mutant in a wild-type leaf. Finally, the probability that the number of mutant leaves decreases by 1 if the center is wild-type is

$$T_{(0,i)\to(0,i-1)} = \frac{m_O i}{m_I i + m_I(D-1-i) + m_O(D-1)} \rho_W^L \,, \tag{S111}$$

where ρ_W^L is the probability of fixation of one wild-type individual in a mutant leaf.

As in Ref. [6], we call $\Phi_{0,i}^{\text{star}}$ (resp. $\Phi_{1,i}^{\text{star}}$) the fixation probability of the mutant type starting from i fully mutant leaves and a wild-type center (resp. a mutant center). The fixation probabilities $\Phi_{0,i}^{\text{star}}$ and $\Phi_{1,i}^{\text{star}}$ satisfy the following recurrence relationship (see Refs. [6, 13]):

$$\begin{cases} \Phi_{0,0}^{\text{star}} = 0 \,, \\ \Phi_{1,i}^{\text{star}} = T_{(1,i)\to(0,i)} \Phi_{0,i}^{\text{star}} + T_{(1,i)\to(1,i+1)} \Phi_{1,i+1}^{\text{star}} \\ \qquad + \left[1 - T_{(1,i)\to(0,i)} - T_{(1,i)\to(1,i+1)}\right] \Phi_{1,i}^{\text{star}} \text{ for } 0 \le i \le D-2 \,, \\ \Phi_{0,i}^{\text{star}} = T_{(0,i)\to(1,i)} \Phi_{1,i}^{\text{star}} + T_{(0,i)\to(0,i-1)} \Phi_{0,i-1}^{\text{star}} \\ \qquad + \left[1 - T_{(0,i)\to(1,i)} - T_{(0,i)\to(0,i-1)}\right] \Phi_{0,i}^{\text{star}} \text{ for } 1 \le i \le D-1 \,, \\ \Phi_{1,D-1}^{\text{star}} = 1 \,, \end{cases} \tag{S112}$$

which yields:

$$\begin{cases} \Phi_{0,0}^{\text{star}} = 0 \,, \\ \Phi_{1,i}^{\text{star}} = \dfrac{-1 + \Gamma_1 \left[-1 + (1+\Gamma_0) \left(\frac{\Gamma_0(1+\Gamma_1)}{1+\Gamma_0}\right)^i\right]}{-1 + \Gamma_1 \left[-1 + (1+\Gamma_0) \left(\frac{\Gamma_0(1+\Gamma_1)}{1+\Gamma_0}\right)^{D-1}\right]} \text{ for } 0 \le i \le D-2 \,, \\ \Phi_{0,i}^{\text{star}} = \dfrac{(1+\Gamma_1) \left[-1 + \left(\frac{\Gamma_0(1+\Gamma_1)}{1+\Gamma_0}\right)^i\right]}{-1 + \Gamma_1 \left[-1 + (1+\Gamma_0) \left(\frac{\Gamma_0(1+\Gamma_1)}{1+\Gamma_0}\right)^{D-1}\right]} \text{ for } 1 \le i \le D-1 \,, \\ \Phi_{1,D-1}^{\text{star}} = 1 \,, \end{cases} \tag{S113}$$

with

$$\Gamma_1 = \alpha \frac{\rho_W^C}{\rho_M^L} \,, \tag{S114}$$

$$\Gamma_0 = \frac{1}{\alpha} \frac{\rho_W^L}{\rho_M^C} \,, \tag{S115}$$

where we have used migration asymmetry

$$\alpha = \frac{m_I}{m_O} \,. \tag{S116}$$

Let us now consider the probability ρ_1^{star} that a single mutant, appearing uniformly at random in the population, fixes in the whole population. In the rare migration regime, it first fixes in the deme where it appeared, and then it spreads. Let us denote by ρ_M^C and ρ_M^L, respectively, the fixation probability of a single mutant in the center and in a leaf. Note that they depend on whether we consider a Moran-like model (as in Ref. [6]) or a Wright-Fisher-like model (as our dilution model introduced in [1] and used here). For consistency with the rest of the paper, we employ the serial dilution model, yielding, for a single mutant placed in the center at a bottleneck:

$$\rho_M^C = \frac{1 - e^{-2\delta_C st}}{1 - e^{-2K\delta_C st}} \text{ and } \rho_W^C = \frac{1 - e^{2\delta_C st}}{1 - e^{2K\delta_C st}} \,, \tag{S117}$$

and similarly for ρ_M^L, but with δ_L taking the place of δ_C, if the mutant starts in a leaf. Here, we have denoted by $\delta_C st$ and $\delta_L st$ the mutant effective relative fitness advantages in the center and in a leaf, respectively.

Then, we have:

$$\rho_1^{\text{star}} = \frac{1}{D}\rho_M^C \Phi_{1,0}^{\text{star}} + \frac{D-1}{D}\rho_M^L \Phi_{0,1}^{\text{star}}\,, \tag{S118}$$

with $\Phi_{1,0}^{\text{star}}$ and $\Phi_{0,1}^{\text{star}}$ given in Eq. S113.

8.1.2 Amplification of selection

Figure 6 shows that the heterogeneous star in the rare migration regime is an amplifier of selection compared to a homogeneous circulation with the same mean mutant fitness when δ_C is substantially larger than δ_L, i.e. when beneficial (resp. deleterious) mutants are substantially more advantaged (resp. disadvantaged) in the center than in leaves. We observe this amplification for negative and for small absolute baseline mutant fitness advantages, corresponding to weakly deleterious and weakly beneficial mutants, in a broad range of migration asymmetries $\alpha = m_I/m_O$. Recall that the homogeneous star is a suppressor of selection for $\alpha < 1$ and an amplifier of selection for $\alpha > 1$ [6], and that the amplification effect is observed under rare migrations for $\alpha > 1$ when $st \lesssim 1/K$ [1]. To better understand these results, let us consider different selection regimes, under rare migrations, and assuming $s > 0$.

First, if $1/K \ll 2\delta_C st \ll 1$ and $1/K \ll 2\delta_L st \ll 1$, then the fixation probabilities in a single deme (see Eq. S117) reduce to

$$\rho_M^C \approx 1 - e^{-2\delta_C st} \approx 2\delta_C st - 2\delta_C^2 s^2 t^2 \quad \text{and} \quad \rho_W^C \approx 0\,, \tag{S119}$$

and similarly in the leaf (note that ρ_W^C is in fact exponentially suppressed). In this regime, once a mutant has fixed in a deme, the wild type is unable to retake over that deme, and fixation in the whole population always ensues. Thus, the mutant fixation probability in the star, averaged over the deme where the mutant starts, reads:

$$\rho \approx \frac{1}{D}\rho_M^C + \frac{D-1}{D}\rho_M^L \approx 2\langle\delta\rangle st - 2\langle\delta^2\rangle s^2 t^2\,. \tag{S120}$$

Under our assumptions, we have $1/K \ll 2\langle\delta\rangle st \ll 1$, and hence, we can express the difference of fixation probability between a heterogeneous and a homogeneous structure with the same $\langle\delta\rangle$ as:

$$\rho - \rho^{\text{homo}} \approx -2(st)^2\left(\langle\delta^2\rangle - \langle\delta\rangle^2\right) < 0\,, \tag{S121}$$

which demonstrates that in this regime, environment heterogeneity leads to suppression of selection, and explains the observations of Figure 6B for larger st.

Second, if $\delta_C > \delta_L$, there may be a regime where $1/K \ll 2\delta_C st \ll 1$ but $2\delta_L st \lesssim 1/K$. In this case, the situation is the same as before for a mutant starting in the center: if it fixes there, it should ultimately take over the whole population. Indeed, wild-type individuals migrating to the center cannot take over due to their substantial fitness disadvantage (their fixation probability is exponentially suppressed). However, the situation is different for a mutant starting in a leaf: once it has fixed there, it may be re-invaded by wild-type individuals from the center, before the mutant can fix in the center. In a homogeneous circulation with the same $\langle\delta\rangle$, if D is large enough, we will have $2\langle\delta\rangle st \lesssim 1/K$, as in a leaf, and thus the homogeneous circulation will not benefit from a "safe" deme for mutants: this leads to amplification in this regime. Moreover, the heterogeneous star structure with $\delta_C > \delta_L$ means that a mutant starting in a leaf will take over the whole structure if it just takes over the center, making this structure quite favorable to mutant fixation in this regime. Takeover of the center starting from a leaf is more likely if α is large, leading to more amplification in that case.

8.2 Line with heterogeneous environment

8.2.1 Mutant fixation probability

Here, we extend our derivation of mutant fixation probability in a line with rare migrations, made in the homogeneous case in Ref. [14], to heterogeneous case.

Equation on the fixation probability. Let us consider a line graph made of D demes in the rare migration regime. We denote by m_L (respectively m_R) the migration rate to the left (respectively to the right). We introduce environment heterogeneity by considering D_L demes on the left-hand side of the line where the mutant relative fitness advantage is $\delta_L s$, and $D_R = D - D_L$ demes on the right-hand side where the mutant relative fitness advantage is $\delta_R s$. Considering the serial dilution model, the fixation probability of one mutant (resp. wild-type) in a wild-type (resp. mutant) deme is

$$\rho_M^b = \frac{1-e^{-2\delta_b st}}{1-e^{-2K\delta_b st}} \qquad \text{and} \qquad \rho_W^b = \frac{1-e^{2\delta_b st}}{1-e^{2K\delta_b st}}, \tag{S122}$$

with $b \in \{L, R\}$.

As pointed out in Ref. [14], in the rare migration regime, the fact that mutants have to spread from the deme where the mutant appeared to other ones in order to fix entails that, at all times, there is one contiguous cluster of demes of mutants. Let us denote by $\phi_{i,j}$ the probability that mutants fix in the line starting from a cluster of $j-i$ adjacent fully mutant demes. Here, $i \in [0, D]$ indicates the position of the first mutant deme along the line, while $j \in [0, D]$ is the position of the first wild-type deme along the line, with the constraint that $i \leq j$ (the case $j = D$ corresponds to a situation where there are no wild-type demes to the right of the mutant cluster). Note that exceptionally, in this derivation, demes are numbered from 0 to $D-1$.

The equation satisfied by the fixation probability $\phi_{i,j}$ is

$$\phi_{i,j} = p_1\phi_{i,j+1} + p_2\phi_{i-1,j} + p_3\phi_{i,j-1} + p_4\phi_{i+1,j}\,, \tag{S123}$$

where the indices i, j correspond respectively to the position of the first mutant (type M) deme and the position of the next first wild type (type W). Here, in contrast with the homogeneous case addressed in Ref. [14], the transition probabilities p_1, p_2, p_3, p_4 depend on where the cluster of mutant demes is located, because of the environment heterogeneity. Specifically, they depend on the zones where the indices i, j are located, represented in Fig. S15.

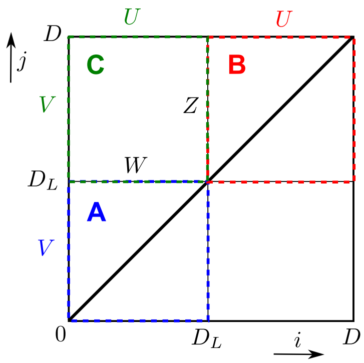

Figure S15: **Schematic of the zones where the indices i and j can vary.** Each zone (A, B, C) is associated with specific values of the transition probabilities p_1, p_2, p_3, and p_4. Boundary conditions (U, V, W, Z) are shown. The black diagonal corresponds to mutant extinction, where $\phi_{i,i} = 0$ for all $i \in [0, D]$, while the upper-left corner corresponds to mutant fixation on the graph, with $\phi_{0,D} = 1$.

Their expressions in these different zones, illustrated in Fig. S16, are the following:

- Zone A ($0 < i < j < D_L$):

$$p_1^A = \frac{\alpha}{(1+\alpha)(1+\gamma_L)}, p_2^A = \frac{1}{(1+\alpha)(1+\gamma_L)}, \tag{S124}$$

$$p_3^A = \frac{\gamma_L}{(1+\alpha)(1+\gamma_L)}, p_4^A = \frac{\alpha\gamma_L}{(1+\alpha)(1+\gamma_L)}, \tag{S125}$$

- Zone B ($D_L < i < j < D$):

$$p_1^B = \frac{\alpha}{(1+\alpha)(1+\gamma_R)}, p_2^B = \frac{1}{(1+\alpha)(1+\gamma_R)}, \tag{S126}$$

$$p_3^B = \frac{\gamma_R}{(1+\alpha)(1+\gamma_R)}, p_4^B = \frac{\alpha\gamma_R}{(1+\alpha)(1+\gamma_R)}, \tag{S127}$$

- Zone C ($i < D_L, j > D_L$):

$$p_1^C = \frac{\alpha\beta_M}{1+\gamma_R\beta_M+\alpha(\gamma_L+\beta_M)}, p_2^C = \frac{1}{1+\gamma_R\beta_M+\alpha(\gamma_L+\beta_M)}, \tag{S128}$$

$$p_3^C = \frac{\gamma_R\beta_M}{1+\gamma_R\beta_M+\alpha(\gamma_L+\beta_M)}, p_4^C = \frac{\alpha\gamma_L}{1+\gamma_R\beta_M+\alpha(\gamma_L+\beta_M)}, \tag{S129}$$

with migration asymmetry $\alpha = m_R/m_L$, and introducing $\beta_a = \rho_a^R/\rho_a^L$ ($a \in (W, M)$), and $\gamma_b = \rho_W^b/\rho_M^b$, ($b \in (L, R)$). The boundary conditions are denoted by $\phi_{i,i} = 0$, $\phi_{0,j} = V_j$, $\phi_{i,D} = U_i$, see Fig. S15, where U_i and V_j are expressed below.

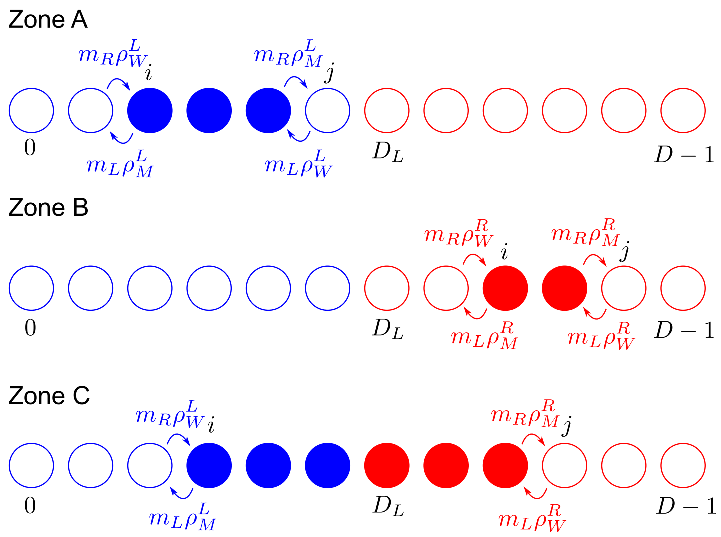

Figure S16: Example configurations for each region A, B and C.

Boundary conditions.

- Expression of $V_j = \phi_{0,j}$:

$$\begin{cases} (\alpha+\gamma_L)V_j = \alpha V_{j+1} + \gamma_L V_{j-1} \text{ , if } j < D_L, \\ (\alpha+\gamma_R)V_j = \alpha V_{j+1} + \gamma_R V_{j-1} \text{ , if } j > D_L, \end{cases} \tag{S130}$$

and for $j = D_L$,

$$(\alpha\beta_M + \gamma_L)V_{D_L} = \alpha\beta_M V_{D_L+1} + \gamma_L V_{D_L-1}. \tag{S131}$$

The solution is

$$V_j = \begin{cases} A_L + B_L \left(\frac{\gamma_L}{\alpha}\right)^j \text{ , if } j < D_L, \\ A_R + B_R \left(\frac{\gamma_R}{\alpha}\right)^j \text{ , if } j > D_L. \end{cases} \tag{S132}$$

The boundary conditions are $A_L + B_L = 0$ and $A_R + B_R \left(\frac{\gamma_R}{\alpha}\right)^D = 1$. Branching left and right solutions imposes that $A_L + B_L \left(\frac{\gamma_L}{\alpha}\right)^{D_L} = A_R + B_R \left(\frac{\gamma_R}{\alpha}\right)^{D_L}$. It yields

$$A_L = \frac{1}{Z_V}\beta_M \left(\frac{\gamma_R}{\gamma_L}\right)^{D_L} \left(1 - \frac{\gamma_R}{\alpha}\right), \tag{S133}$$

$$B_L = -\frac{1}{Z_V}\beta_M \left(\frac{\gamma_R}{\gamma_L}\right)^{D_L} \left(1 - \frac{\gamma_R}{\alpha}\right), \tag{S134}$$

$$A_R = \frac{1}{Z_V}\left[\beta_M \left(\frac{\gamma_R}{\gamma_L}\right)^{D_L} \left(1 - \frac{\gamma_R}{\alpha}\right)\left(1 - \left(\frac{\gamma_L}{\alpha}\right)^{D_L}\right) + \left(1 - \frac{\gamma_L}{\alpha}\right)\left(\frac{\gamma_R}{\alpha}\right)^{D_L}\right], \tag{S135}$$

$$B_R = \frac{1}{Z_V}\left(1 - \frac{\gamma_L}{\alpha}\right), \tag{S136}$$

with

$$Z_V = \beta_M \left(\frac{\gamma_R}{\gamma_L}\right)^{D_L} \left(1 - \frac{\gamma_R}{\alpha}\right)\left(1 - \left(\frac{\gamma_L}{\alpha}\right)^{D_L}\right) + \left(1 - \frac{\gamma_L}{\alpha}\right)\left(\left(\frac{\gamma_R}{\alpha}\right)^{D_L} - \left(\frac{\gamma_R}{\alpha}\right)^{D}\right). \tag{S137}$$

- Expression of $U_i = \phi_{i,D}$:

$$\begin{cases} (1+\alpha\gamma_L)U_i = \alpha\gamma_L U_{i+1} + U_{i-1} \text{ , if } i < D_L, \\ (1+\alpha\gamma_R)U_i = \alpha\gamma_R U_{i+1} + U_{i-1} \text{ , if } i > D_L, \end{cases} \tag{S138}$$

and for $i = D_L$

$$(1+\alpha\beta_M\gamma_R)\,U_{D_L} = \alpha\beta_M\gamma_R U_{D_L+1} + U_{D_L-1}. \tag{S139}$$

The general solution is

$$U_i = \begin{cases} E_L + \frac{F_L}{(\alpha\gamma_L)^i} \text{ , if } i < D_L, \\ E_R + \frac{F_R}{(\alpha\gamma_R)^i} \text{ , if } i > D_L. \end{cases} \tag{S140}$$

The boundary conditions are $E_L + F_L = 1$ and $E_R + \dfrac{F_R}{(\alpha\gamma_R)^D} = 0$. Branching both solutions imposes that $E_L + \dfrac{F_L}{(\alpha\gamma_L)^{D_L}} = E_R + \dfrac{F_R}{(\alpha\gamma_R)^{D_L}}$. It yields

$$E_L = \frac{1}{Z_U}\left[-\frac{\beta_M}{(\alpha\gamma_L)^{D_L}}\left(1 - \frac{1}{\alpha\gamma_R}\right) + \left(\frac{\gamma_R}{\gamma_L}\right)^{D_L-1}\left(1 - \frac{1}{\alpha\gamma_L}\right)\left(\frac{1}{(\alpha\gamma_R)^{D_L}} - \frac{1}{(\alpha\gamma_R)^{D}}\right)\right], \tag{S141}$$

$$F_L = \frac{1}{Z_U}\beta_M\left(1 - \frac{1}{\alpha\gamma_R}\right), \tag{S142}$$

$$E_R = -\frac{1}{Z_U}\left(\frac{\gamma_R}{\gamma_L}\right)^{D_L-1}\left(1 - \frac{1}{\alpha\gamma_L}\right)\frac{1}{(\alpha\gamma_R)^D}, \tag{S143}$$

$$F_R = \frac{1}{Z_U}\left(\frac{\gamma_R}{\gamma_L}\right)^{D_L-1}\left(1 - \frac{1}{\alpha\gamma_L}\right), \tag{S144}$$

with

$$Z_U = \beta_M \left(1 - \frac{1}{\alpha\gamma_R}\right)\left(1 - \frac{1}{(\alpha\gamma_L)^{D_L}}\right) + \left(\frac{\gamma_R}{\gamma_L}\right)^{D_L-1}\left(1 - \frac{1}{\alpha\gamma_L}\right)\left(\frac{1}{(\alpha\gamma_R)^{D_L}} - \frac{1}{(\alpha\gamma_R)^{D}}\right). \tag{S145}$$

Expression of $\phi_{i,j}$ in the different zones. In each zone, the fixation probability is given by [15]

$$\phi^k_{i,j} = \sum_{a=1}^{L^k_x-1}\left[p^k_1\phi_{a,L^k_y}T^k_{(a,L^k_y-1)i,j} + p^k_3\phi_{a,0}T^k_{(a,1)i,j}\right] + \sum_{b=1}^{L^k_y-1}\left[p^k_2\phi_{0,b}T^k_{(1,b)i,j} + p^k_4\phi_{L^k_x,a}T^k_{(L^k_x-1,b)i,j}\right] \tag{S146}$$

with

$$T^k_{(a,b)i,j} = \frac{4}{L^k_xL^k_y}\left(\frac{p^k_2}{p^k_4}\right)^{\frac{i-a}{2}}\left(\frac{p^k_3}{p^k_1}\right)^{\frac{j-b}{2}}\sum_{r=1}^{L^k_x-1}\sum_{s=1}^{L^k_y-1}\frac{\sin\left(\frac{ir\pi}{L^k_x}\right)\sin\left(\frac{ar\pi}{L^k_x}\right)\sin\left(\frac{bs\pi}{L^k_y}\right)\sin\left(\frac{js\pi}{L^k_y}\right)}{1-2\sqrt{p^k_2p^k_4}\cos\left(\frac{r\pi}{L^k_x}\right)-2\sqrt{p^k_1p^k_3}\cos\left(\frac{s\pi}{L^k_y}\right)}, \tag{S147}$$

with $k \in (A, B, C)$, $i \in [1, ..., L^k_x - 1]$ and $j \in [1, ..., L^k_y - 1]$.

- Zone A ($0 < i, j < D_L$): $L^A_x = L^A_y = D_L$

$$\begin{aligned}\phi^A_{i,j} &= \sum_{a=1}^{D_L-1}\left[p^A_1\phi_{a,D_L}T^A_{(a,D_L-1)i,j} + p^A_2\phi_{0,a}T^A_{(1,a)i,j} + p^A_3\phi_{a,0}T^A_{(a,1)i,j} + p^A_4\phi_{D_L,a}T^A_{(D_L-1,a)i,j}\right]\\ &= \sum_{a=1}^{D_L-1}\left[p^A_1W_aT^A_{(a,D_L-1)i,j} + p^A_2V_aT^A_{(1,a)i,j} + p^A_3\phi_{a,0}T^A_{(a,1)i,j} + p^A_4\phi_{D_L,a}T^A_{(D_L-1,a)i,j}\right],\end{aligned} \tag{S148}$$

where $V_a = \phi_{0,a}$ was computed previously, but $W_a = \phi_{a,D_L}$ is unknown for now. We determine $\phi_{a,0}$ and $\phi_{D_L,a}$ using the constraint

$$\phi^A_{i,i} = 0 = \sum_{a=1}^{D_L-1}\left[p^A_1W_aT^A_{(a,D_L-1)i,i} + p^A_2V_aT^A_{(1,a)i,i} + p^A_3\phi_{a,0}T^A_{(a,1)i,i} + p^A_4\phi_{D_L,a}T^A_{(D_L-1,a)i,i}\right], \tag{S149}$$

and the property $T_{(a,b)i,i} = \gamma_L^{a-b}T_{(b,a)i,i}$, which yields

$$0 = \sum_{a=1}^{D_L-1}\left[T^A_{(a,D_L-1)i,i}\left(p^A_1W_a + p^A_4\phi_{D_L,a}\gamma_L^{D_L-1-a}\right) + T^A_{(a,1)i,i}\left(p^A_3\phi_{a,0} + p^A_2V_a\gamma_L^{1-a}\right)\right]. \tag{S150}$$

Building on Ref. [13], one can determine the solution:

$$\phi_{D_L,a} = -\gamma_L^{a-D_L}W_a, \tag{S151}$$

$$\phi_{a,0} = -\gamma_L^{-a}V_a. \tag{S152}$$

Finally,

$$\phi^A_{i,j} = \sum_{a=1}^{D_L-1} p^A_1W_a\left[T^A_{(a,D_L-1)i,j} - \gamma_L^{a-D_L+1}T^A_{(D_L-1,a)i,j}\right] + p^A_2V_a\left[T^A_{(1,a)i,j} - \gamma_L^{1-a}T^A_{(a,1)i,j}\right]. \tag{S153}$$

- Zone B ($D_L < i, j < D$): $L_x^B = L_y^B = D - D_L = D_R$

$$\begin{aligned}
\phi^B_{i+D_L,j+D_L} &= \sum_{a=1}^{D_R-1} \Big[p_1^B \phi_{a+D_L,D} T^B_{(a,D_R-1)i,j} + p_2^B \phi_{D_L,a+D_L} T^B_{(1,a)i,j} \\
&\quad + p_3^B \phi_{a+D_L,D_L} T^B_{(a,1)i,j} + p_4^B \phi_{D,a+D_L} T^B_{(D_R-1,a)i,j}\Big] \\
&= \sum_{a=1}^{D_R-1} \Big[p_1^B U_{a+D_L} T^B_{(a,D_R-1)i,j} + p_2^B Z_{a+D_L} T^B_{(1,a)i,j} \\
&\quad + p_3^B \phi_{a+D_L,D_L} T^B_{(a,1)i,j} + p_4^B \phi_{D,a+D_L} T^B_{(D_R-1,a)i,j}\Big], \qquad \text{(S154)}
\end{aligned}$$

where $Z_{a+D_L} = \phi_{D_L,a+D_L}$, which is unknown. Using the same technique as for Zone A, we find

$$\phi_{D,a+D_L} = -\gamma_R^{a-D_R} U_{a+D_L}, \qquad \text{(S155)}$$

$$\phi_{a+D_L,D_L} = -\gamma_R^{-a} Z_{a+D_L}. \qquad \text{(S156)}$$

Finally,

$$\begin{aligned}
\phi^B_{i+D_L,j+D_L} &= \sum_{a=1}^{D_R-1} p_1^B U_{a+D_L} \left[T^B_{(a,D_R-1)i,j} - \gamma_R^{a-D_R+1} T^B_{(D_R-1,a)i,j}\right] \\
&\quad + p_2^B Z_{a+D_L} \left[T^B_{(1,a)i,j} - \gamma_R^{1-a} T^B_{(a,1)i,j}\right]. \qquad \text{(S157)}
\end{aligned}$$

- Zone C ($0 < i < D_L$, $D_L < j < D$): $L_x^C = D_L$, $L_y^C = D_R$

$$\begin{aligned}
\phi^C_{i,j+D_L} &= \sum_{a=1}^{D_L-1} \left[p_1^C \phi_{a,D} T^C_{(a,D_R-1)i,j} + p_3^C \phi_{a,D_L} T^C_{(a,1)i,j}\right] \\
&\quad + \sum_{b=1}^{D_R-1} \left[p_2^C \phi_{0,b+D_L} T^C_{(1,b)i,j} + p_4^C \phi_{D_L,b+D_L} T^C_{(D_L-1,b)i,j}\right] \\
&= \sum_{a=1}^{D_L-1} \left[p_1^C U_a T^C_{(a,D_R-1)i,j} + p_3^C W_a T^C_{(a,1)i,j}\right] \\
&\quad + \sum_{b=1}^{D_R-1} \left[p_2^C V_{b+D_L} T^C_{(1,b)i,j} + p_4^C Z_{b+D_L} T^C_{(D_L-1,b)i,j}\right]. \qquad \text{(S158)}
\end{aligned}$$

Equations on W and Z. We would like to determine the remaining unknown boundary conditions W and Z. First, the equation on W (see Fig. S17) is, for all $i \in [1, D_L - 1]$:

$$W_i = \phi_{i,D_L} = p_1^W \phi_{i,D_L+1} + p_2^W \phi_{i-1,D_L} + p_3^W \phi_{i,D_L-1} + p_4^W \phi_{i+1,D_L}, \qquad \text{(S159)}$$

with

$$p_1^W = \frac{\alpha\beta_M}{1+\gamma_L+\alpha(\beta_M+\gamma_L)}, p_2^W = \frac{1}{1+\gamma_L+\alpha(\beta_M+\gamma_L)}, \qquad \text{(S160)}$$

$$p_3^W = \frac{\gamma_L}{1+\gamma_L+\alpha(\beta_M+\gamma_L)}, p_4^W = \frac{\alpha\gamma_L}{1+\gamma_L+\alpha(\beta_M+\gamma_L)}, \qquad \text{(S161)}$$

where $W_0 = V_{D_L}$ and $W_{D_L} = 0$.

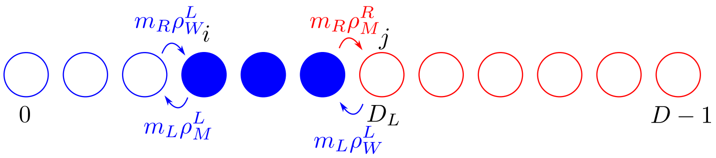

Figure S17: Example configuration in the specific case where $j = D_L$.

Eq. S159 can be rewritten as:

$$\begin{aligned}
W_i &= p_1^W \phi^C_{i,D_L+1} + p_2^W W_{i-1} + p_3^W \phi^A_{i,D_L-1} + p_4^W W_{i+1} \\
&= p_1^W \Bigg\{ \sum_{a=1}^{D_L-1} \left[p_1^C U_a T^C_{(a,D_R-1)i,1} + p_3^C W_a T^C_{(a,1)i,1} \right] \\
&\quad + \sum_{b=1}^{D_R-1} \left[p_2^C V_{b+D_L} T^C_{(1,b)i,1} + p_4^C Z_{b+D_L} T^C_{(D_L-1,b)i,1} \right] \Bigg\} \\
&\quad + p_3^W \Bigg\{ \sum_{a=1}^{D_L-1} p_1^A W_a \left[T^A_{(a,D_L-1)i,D_L-1} - \gamma_L^{a-D_L+1} T^A_{(D_L-1,a)i,D_L-1} \right] \\
&\quad + p_2^A V_a \left[T^A_{(1,a)i,D_L-1} - \gamma_L^{1-a} T^A_{(a,1)i,D_L-1} \right] \Bigg\} \\
&\quad + p_2^W W_{i-1} + p_4^W W_{i+1}. \qquad \text{(S162)}
\end{aligned}$$

Similarly, the equation on Z (see Fig. S18) is, for all $j \in [1, D_R - 1]$:

$$Z_{j+D_L} = \phi_{D_L,j+D_L} = p_1^Z \phi_{D_L,j+D_L+1} + p_2^Z \phi_{D_L-1,j+D_L} + p_3^Z \phi_{D_L,j+D_L-1} + p_4^Z \phi_{D_L+1,j+D_L}, \qquad \text{(S163)}$$

with

$$p_1^Z = \frac{\alpha}{\gamma_R + 1/\beta_M + \alpha(1+\gamma_R)}, \qquad p_2^Z = \frac{1/\beta_M}{\gamma_R + 1/\beta_M + \alpha(1+\gamma_R)}, \qquad \text{(S164)}$$

$$p_3^Z = \frac{\gamma_R}{\gamma_R + 1/\beta_M + \alpha(1+\gamma_R)}, \qquad p_4^Z = \frac{\alpha\gamma_R}{\gamma_R + 1/\beta_M + \alpha(1+\gamma_R)}. \qquad \text{(S165)}$$

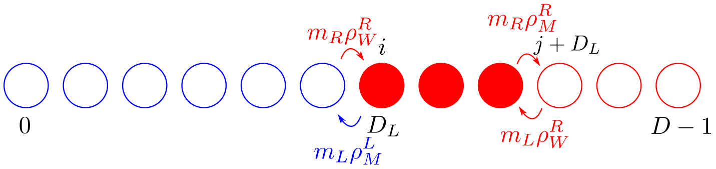

Figure S18: Example configuration in the specific case where $i = D_L$.

Again, we find

$$\begin{aligned}
Z_{j+D_L} &= p_1^Z Z_{j+D_L+1} + p_2^Z \phi^C_{D_L-1,j+D_L} + p_3^Z Z_{j+D_L-1} + p_4^Z \phi^B_{D_L+1,j+D_L} \\
&= p_2^Z \Bigg\{ \sum_{a=1}^{D_L-1} \left[p_1^C U_a T^C_{(a,D_R-1)D_L-1,j} + p_3^C W_a T^C_{(a,1)D_L-1,j} \right] \\
&\quad + \sum_{b=1}^{D_R-1} \left[p_2^C V_{b+D_L} T^C_{(1,b)D_L-1,j} + p_4^C Z_{b+D_L} T^C_{(D_L-1,b)D_L-1,j} \right] \Bigg\} \\
&\quad + p_4^Z \Bigg\{ \sum_{a=1}^{D_R-1} p_1^B U_{a+D_L} \left[T^B_{(a,D_R-1)1,j} - \gamma_R^{a-D_R+1} T^B_{(D_R-1,a)1,j} \right] \\
&\quad + p_2^B Z_{a+D_L} \left[T^B_{(1,a)1,j} - \gamma_R^{1-a} T^B_{(a,1)1,j} \right] \Bigg\} \\
&\quad + p_1^Z Z_{j+D_L+1} + p_3^Z Z_{j+D_L-1}. \qquad \text{(S166)}
\end{aligned}$$

It is convenient to rewrite Eqs. S162 and S166 in the form of a linear system:

$$\sum_{k=0}^{D_L} A_{i,k} W_k + \sum_{\ell=0}^{D_R} B_{i,\ell} Z_{\ell+D_L} = E_i \qquad (0 \leq i \leq D_L), \tag{S167}$$

$$\sum_{k=0}^{D_L} C_{j,k} W_k + \sum_{\ell=0}^{D_R} D_{j,\ell} Z_{\ell+D_L} = F_j \qquad (0 \leq j \leq D_R). \tag{S168}$$

where for all $0 < i < D_L$ and $0 < j < D_R$:

$$\begin{aligned} A_{i,k} = {} & p_3^W p_1^A \left[T^A_{(k,D_L-1)i,D_L-1} - \gamma_L^{k-D_L+1} T^A_{(D_L-1,k)i,D_L-1} \right] \\ & + p_1^W p_3^C T^C_{(k,1)i,1} + p_2^W \delta_{k,i-1} + p_4^W \delta_{k,i+1} - \delta_{k,i}, \end{aligned} \tag{S169}$$

$$B_{i,\ell} = p_1^W p_4^C T^C_{(D_L-1,\ell)i,1}, \tag{S170}$$

$$C_{j,k} = p_2^Z p_3^C T^C_{(k,1)D_L-1,j}, \tag{S171}$$

$$\begin{aligned} D_{j,\ell} = {} & p_4^Z p_2^B \left[T^B_{(1,\ell)1,j} - \gamma_R^{1-\ell} T^B_{(\ell,1)1,j} \right] \\ & + p_2^Z p_4^C T^C_{(D_L-1,\ell)D_L-1,j} + p_1^Z \delta_{\ell,j+1} + p_3^Z \delta_{\ell,j-1} - \delta_{\ell,j}, \end{aligned} \tag{S172}$$

$$\begin{aligned} E_i = {} & -p_1^W p_1^C \sum_{a=1}^{D_L-1} U_a T^C_{(a,D_R-1)i,1} - p_1^W p_2^C \sum_{b=1}^{D_R-1} V_{b+D_L} T^C_{(1,b)i,1} \\ & - p_3^W p_2^A \sum_{a=1}^{D_L-1} V_a \left[T^A_{(1,a)i,D_L-1} - \gamma_L^{1-a} T^A_{(a,1)i,D_L-1} \right], \end{aligned} \tag{S173}$$

$$\begin{aligned} F_j = {} & -p_2^Z p_1^C \sum_{a=1}^{D_L-1} U_a T^C_{(a,D_R-1)D_L-1,j} - p_2^Z p_2^C \sum_{b=1}^{D_R-1} V_{b+D_L} T^C_{(1,b)D_L-1,j} \\ & - p_4^Z p_1^B \sum_{a=1}^{D_R-1} U_{a+D_L} \left[T^B_{(a,D_R-1)1,j} - \gamma_R^{a-D_R+1} T^B_{(D_R-1,a)1,j} \right], \end{aligned} \tag{S174}$$

and

$$A_{0,k} = \delta_{0,k}, \qquad A_{D_L,k} = \delta_{D_L,k}, \qquad D_{0,\ell} = \delta_{0,\ell}, \qquad D_{D_R,\ell} = \delta_{D_R,\ell}, \tag{S175}$$

$$B_{0,\ell} = 0, \qquad B_{D_L,\ell} = 0, \qquad C_{0,k} = 0, \qquad C_{D_R,k} = 0, \tag{S176}$$

$$E_0 = V_{D_L}, \qquad E_{D_L} = 0, \qquad F_0 = 0, \qquad F_{D_R} = U_{D_L}. \tag{S177}$$

Conclusion. Numerically solving the linear system in Eqs. S167 and S168 (using matrix inversion) provides the values of W and Z. Then, the fixation probability of mutants, starting from one mutant placed uniformly at random in the line graph is

$$\rho_1^{\text{line}} = \frac{1}{D} \left\{ \rho_M^L \left[V_1 + \sum_{i=1}^{D_L-2} \phi^A_{i,i+1} + W_{D_L-1} \right] + \rho_M^R \left[Z_{D_L+1} + \sum_{i=D_L+1}^{D-2} \phi^B_{i,i+1} + U_{D-1} \right] \right\}, \tag{S178}$$

with

$$\begin{aligned} \phi^A_{i,i+1} = {} & \sum_{a=1}^{D_L-1} p_1^A W_a \left[T^A_{(a,D_L-1)i,i+1} - \gamma_L^{a-D_L+1} T^A_{(D_L-1,a)i,i+1} \right] \\ & + \sum_{a=1}^{D_L-1} p_2^A V_a \left[T^A_{(1,a)i,i+1} - \gamma_L^{1-a} T^A_{(a,1)i,i+1} \right], \end{aligned} \tag{S179}$$

$$\begin{aligned} \phi^B_{i+D_L,i+D_L+1} = {} & \sum_{a=1}^{D_R-1} p_1^B U_{a+D_L} \left[T^B_{(a,D_R-1)i,i+1} - \gamma_R^{a-D_R+1} T^B_{(D_R-1,a)i,i+1} \right] \\ & + \sum_{a=1}^{D_R-1} p_2^B Z_{a+D_L} \left[T^B_{(1,a)i,i+1} - \gamma_R^{1-a} T^B_{(a,1)i,i+1} \right]. \end{aligned} \tag{S180}$$

8.2.2 Amplification of selection

In Fig. S19, the fixation probability obtained in Eq. S178, for a mutant in the heterogeneous line with rare migrations, is plotted as a function of the baseline relative mutant fitness advantage st. Several distinct regimes emerge as st is increased while $st \ll 1$. First, for very small st, selection is suppressed compared to the homogeneous circulation. Then, for slightly larger st, we observe a small amplification of selection, provided the migration asymmetry is less than 1. Next, suppression is again observed. While these results are complex, due to the interplay of migration and environment heterogeneity and to different regimes of selection, they show that the line can amplify selection in the rare migration regime when the environment is heterogeneous.

A qualitative analysis can be made along the lines of Section 8.1.2. Here, for simplicity, and as in Figure S19, we assume that only one deme on the left has δ_L and the other ones have δ_R.

First, if $1/K \ll 2\delta_L st \ll 1$ and $1/K \ll 2\delta_R st \ll 1$, the difference of fixation probability between a heterogeneous and a homogeneous structure with the same $\langle\delta\rangle$ can be expressed as for the star, see Eq. S121, and thus, environment heterogeneity leads to suppression of selection, explaining the observations of Figure S19 for larger st.

Second, if $\delta_L > \delta_R$, there may be a regime where $1/K \ll 2\delta_L st \ll 1$ but $2\delta_R st \lesssim 1/K$. Following the same reasoning as for the star, we conclude that a mutant starting in the left-most deme will take over in the whole population if it fixes there. Meanwhile, a mutant starting elsewhere that fixes in its original deme may be re-invaded by wild-type individuals. This is more likely in the line than in our heterogeneous star, because there are more ways to get re-invaded before full fixation. Nevertheless, the benefit of having one deme where the mutant is substantially advantaged and safe from re-invasions makes the heterogeneous line slightly amplify selection in this regime, compared to a homogeneous circulation.

Finally, when both δ_L and δ_R are very small, the benefit from having a deme where the mutant is fitter is minimal, and the heterogeneous line behaves as a suppressor of selection, as the homogeneous line [14].

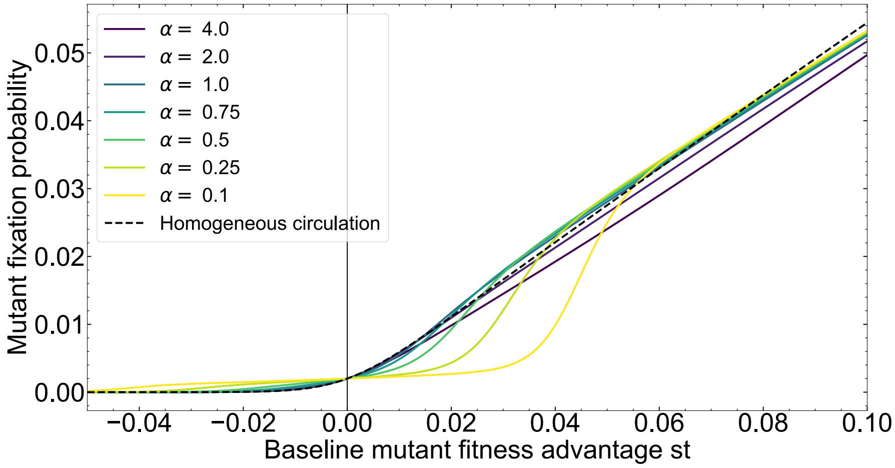

Figure S19: **Fixation probability in the heterogeneous line with rare migrations.** We plot the fixation probability starting from one mutant appearing uniformly at random in the line graph, given by Eq. (S178), as a function of the baseline mutant fitness advantage st. Parameters: $D = 5$, $D_L = 1$, $\delta_L = 1$, $\delta_R = 0.1$, $K = 100$. The case of a homogeneous circulation with the same average mutant relative fitness advantage is shown for reference.

References

[1] Alia Abbara and Anne-Florence Bitbol. Frequent asymmetric migrations suppress natural selection in spatially structured populations. *PNAS Nexus*, 2:pgad392, 2023.

[2] T. E. Harris. *The Theory of Branching Processes*. Springer, 1963.

[3] T. Maruyama. On the fixation probability of mutant genes in a subdivided population. *Genetics Research*, 15(2):221–225, 1970.

[4] T. Maruyama. A simple proof that certain quantities are independent of the geographical structure of population. *Theoretical Population Biology*, 5(2):148–154, 1974.

[5] Erez Lieberman, Christoph Hauert, and Martin A Nowak. Evolutionary dynamics on graphs. *Nature*, 433:312–315, 2005.

[6] Loïc Marrec, Irene Lamberti, and Anne-Florence Bitbol. Toward a Universal Model for Spatially Structured Populations. *Physical Review Letters*, 127(21):218102, 2021.

[7] J. B. S. Haldane. A mathematical theory of natural and artificial selection, part v: Selection and mutation. *Mathematical Proceedings of the Cambridge Philosophical Society*, 23(7):838–844, 1927.

[8] J. F. Crow and M. Kimura. *An Introduction to Population Genetics Theory*. Blackburn, 2009.

[9] L. Hindersin and A. Traulsen. Counterintuitive properties of the fixation time in network-structured populations. *Journal of the Royal Society Interface*, 11:20140606, 2014.

[10] L. Hindersin and A. Traulsen. Most undirected random graphs are amplifiers of selection for birth-death dynamics, but suppressors of selection for death-birth dynamics. *PLOS Computational Biology*, 11(11):1–14, 2015.

[11] Marius Möller, Laura Hindersin, and Arne Traulsen. Exploring and mapping the universe of evolutionary graphs identifies structural properties affecting fixation probability and time. *Communications Biology*, 2(1):137, 2019.

[12] M. Slatkin. Fixation probabilities and fixation times in a subdivided population. *Evolution*, 35(3):477–488, 1981.

[13] Mark Broom and Jan Rychtář. An analysis of the fixation probability of a mutant on special classes of non-directed graphs. *Proceedings of the Royal Society A: Mathematical, Physical and Engineering Sciences*, 464(2098):2609–2627, 2008.

[14] Richard Servajean, Arthur Alexandre, and Anne-Florence Bitbol. Impact of complex spatial population structure on early and long-term adaptation in rugged fitness landscapes. *Evolution*, 79:935–950, 2025.

[15] James W Miller. A matrix equation approach to solving recurrence relations in two-dimensional random walks. *Journal of applied probability*, 31(3):646–659, 1994.
