# Supplementary material for: Environment heterogeneity creates fast amplifiers of natural selection in graph-structured populations
Source: Nat Commun. 2026 May 8;17:6228. doi: 10.1038/s41467-026-72784-z (PMC13369156; doi:10.1038/s41467-026-72784-z)
Supplement: Supplementary file 2 — Transparent Peer Review file [file 41467_2026_72784_MOESM2_ESM.pdf]

nature portfolio

Peer Review File

Environment heterogeneity creates fast amplifiers of natural selection in graph-structured populations

Corresponding Author: Professor Anne-Florence Bitbol

This file contains all reviewer reports in order by version, followed by all author rebuttals in order by version.

Version 0:

Reviewer comments:

Reviewer #1

(Remarks to the Author)
This study examines how environmental heterogeneity influences the fixation of mutants in spatially structured populations, where subpopulations (demes) are located on the nodes of a graph. The research explores how differences in local environments interact with migration between demes to shape evolutionary outcomes, particularly the probability and speed of mutant fixation.

When migration between demes is frequent, environmental heterogeneity is shown to amplify natural selection. Under these conditions, both mutant fixation and extinction occur more rapidly, promoting the swift establishment of beneficial mutants. This effect is demonstrated in specific network structures such as the star graph, even more strongly in the line graph, and also in a broader class of graphs. Amplification arises when mutants possess a greater fitness advantage in demes characterized by stronger migration outflow. In circulation graphs, where migration inflow and outflow are balanced at every deme, environmental heterogeneity exerts no influence at first order but increases the fixation probability of beneficial mutants at second order.

When migration between demes is infrequent, environmental heterogeneity can also enhance the amplification of selection. In this regime, demes that provide a sufficient fitness advantage to mutants may serve as refuges, protecting mutant lineages and facilitating their eventual spread throughout the population.

These are interesting and novel results. The manuscript is very comprehensive and well-written. Overall, I have enjoyed reading and I am in favour of publication subject to the consideration of the following comments.

1) Research on the importance of spatially structured populations in evolution has in the past considered significantly more realistic interaction networks. In this regard the work is missing out on the opportunity to consider more complex networks, beyond the star graph, the line graph, and the currently considered artificial graphs. While I appreciate that these interaction graphs are to a degree still analytically traceable, simulation procedures would allow the consideration of more general networks with properties that correspond more closely to real world complex networks. Scale-free networks, small-world networks, and the most recently considered higher-order networks (e.g. Evolutionary dynamics of higher-order interactions in social networks, Nat. Hum. Behav. 5, 586-595 (2021)) come to mind. The authors should consider this extension to their current research, at least in discussion and future work, if actual results are for some reason unattainable.

2) When referring to the importance of models on graphs and evolutionary graph theory, and in particular that specific graphs, under specific update rules defining the dynamics, can amplify or suppress natural selection, I would recommend the reviews Evolutionary dynamics of group interactions on structured populations: A review, J. R. Soc. Interface 10, 20120997 (2013) and Coevolutionary games - A mini review, BioSystems 99, 109-125 (2010), the later also when authors mention time-varying environments as promising future research avenues.

3) The abstract open prominently with the fact that complex spatial structure and environment heterogeneity shape the evolution of natural populations, but the work then does make clear which are these natural populations the authors have in mind and for which this research would be most applicable to. The applications and implications should be spelled out more explicitly, and I would expect here also a delineation whether this research applies just to biological system or also to social systems.

If a revision is granted, I will be happy to review a revised manuscript.

Reviewer #2

(Remarks to the Author)
Review:

Summary:

The environmental heterogeneity arises in many real-world situations and has a subtle interplay with population structure.
The authors extend their previous work to consider the effects of environmental heterogeneity in structured populations.
They adapt the Wright-Fisher-like model to describe the heterogeneity.
Multiple demes with capacity K are connected in a migration graph.
First, all demes experience exponential growth for time t, where the mutants have deme-dependent fitness \delta_i s for deme i.
Then, to every deme, the new population of size K is sampled from the deme itself and all neighboring demes given by migration probabilities.
The process repeats until complete fixation or extinction of the mutants.

The model is robust and captures many real-world scenarios.
This work is an initial intensive exploration of the model.
The results are not surprising, but they explain the most important scenarios.
In general, it is good work that requires some rewriting.

One important result is for circulations, where the effects of the first order are always the same (proportional to st). The heterogeneity can increase the effects of the second order.

In general, heterogeneous graphs are amplifiers if demes with higher outflow than inflow have a higher than average mutant advantage.

These effects are examined on the star (with simulations).

On the line, there is an elegant formula for the amplification of mutants.
(It is the effect of consecutive biases in the outflows and inflows)

Finally, the rare migration regime is examined where the deme first homogenizes, and then it spreads.

Major Comments:

The model is described in three places: the first paragraph of Results, Models and Methods, and SI, section 1.1.
The results are not understandable without the model described in SI.
The work would benefit from describing the model directly in the Results section.
(It should definitely include Figure S1, at least panel A. I like panel B too.)
To understand any result, knowledge of the model is vital.

The order of the presentation of results in the main text is not good.
The graph that achieves the biggest change in fixation probability (probably deemed the most interesting result) is stated first.
However, there are three sources of heterogeneity: (i) the mutant fitness heterogeneity, (ii) the migration inflow and outflow heterogeneity, and (iii) the graph heterogeneity.
I suggest changing the order of presentation such that points (ii) and (iii) are added first separately and then combined.
(This means the first should be either circulations or complete graphs with strong outflow deme)

There should also be a paragraph (before results) that considers baselines that the results are compared against.
This would unify the results.

Minor Comments:

I would like to see the explanations of the observed and proven effects.
Something like: Inhabitants of a deme with strong outflow are more likely to survive, which means that any increase in fitness in this deme
helps mutants more than in other demes.

I would like to see how the effects change for large D (the size of the deme).
The only figure that varies the constant D is S3.

Some writing is not very inviting, for instance, in SI, section 1.1, end of the third paragraph, the index i appears for m_{ij} and m_{ki} in one sentence.

For the high migration regime, \delta_i \ge 0, what would \delta_i < 0 change in computations?

Reviewer #3

(Remarks to the Author)
Fruet et al., use evolutionary graph theory to understand how different spatial structures impact the fixation probability of beneficial mutations (s) whose advantage over the wild-type differ between demes (d_i), herein called “environment heterogeneity”. In a couple of special spatial structures as well as in a generalized spatial framework, they show that environment heterogeneity can lead to amplifier demes with an increased fixation probability and decreased fixation time; this is in contrast to previous models of homogeneous environments that found amplifier (or suppressor) demes had a positive relationship between fixation probability and fixation time. They identify the conditions when amplification happens (\beta < \alpha < 1 or 1 < \alpha < \beta) and show that these boundaries hold even without strong migrational symmetry. The intuition is that amplifiers demes are ones where mutants are highly beneficial and act either as outflows in the strong migration regime or refugia in the weak regime.

I liked that the authors were able to arrive at results that hold for arbitrary spatial structures and migrational symmetries by starting from a couple of special spatial structures (star and line) and strong migrational asymmetries. The manuscript is motivated by natural microbial populations and, in addition to its mathematical rigour, is understandable to a general audience in theoretical evolution or bacterial population growth.

My main complaint with the manuscript is that it does not provide a clear definition of how environment heterogeneity is conceptualized and implemented straight from the Introduction. Evolutionary readers who are already familiar with the rich literature on environmental heterogeneity, environmental gradients, and gene flow (e.g., Kirkpatrick & Barton 1997 DOI: 10.1086/286054, Lenormand 2002 DOI: 10.1016/S0169-5347(02)02497-7) will be primed to assume that environmental heterogeneity refers to antagonistic selective effects between environments. But this is not the case! For Fruet et al., the mutant is always beneficial even though the strength of the beneficial effect is variable between demes. This is an important novelty of the work and should already be made clear from the Introduction, probably somewhere in the second last paragraph. In addition, it would be nice if the Discussion can verbally contrast the current results with those from population genetics (e.g., the classic works listed above, or newer) or at least address the vast theoretical evolutionary literature on environmental heterogeneity that lies outside of evolutionary graph theory. In particular, I wonder how amplification (or suppression), from the field of evolutionary graph theory, relates to swamping, from the field of population genetics (i.e., if they are/can be related; or otherwise to explain that these concepts are entirely unrelated).

My other comments are relatively minor:

On the bottom of pg 3 the “first order-coefficient (slope) in st” is introduced. This is referred to in the caption of Figure 1 as the “first order-coefficient (slope)” but later on as “first order in st” or simply “first order”. I found the coefficient difficult to understand, and the inconsistent terminology throughout the manuscript made it even more confusing. Consistent phrasing would greatly improve clarity.

I really like the schematics of the spatial models that are included throughout the text, they are incredibly helpful! But it took me a really long time to understand what is \sigma_C in Figure 1A because this value is not clearly defined in the caption and because the schematic focuses the reader’s attention to the migration rates. Maybe find a way to label \sigma_C in the schematic of Figure 1A so that the reader can very quickly understand the whole plot. (It might also be helpful to make the \alpha on the schematic more visible as \alpha is important for panel B and later).

Figure 2. I got confused that the schematics on the right side are referred to in the main text before panel A is referred to. This is especially confusing as these schematics do not have a panel label. I would suggest moving them to the left side and **labeling them** panel A (that way they can be directly referred to in the main text, middle of pg.4).

To help the population geneticists in the audience, note that the “clique” spatial structure was first called the “island model” by Sewall Wright and the “line” called the “stepping stone” by Motoo Kimura.

Figure 3D took me a long time to figure out because the inverse \alpha and inverse \beta values are being used. I know it will be rather annoying to fix this (and seems trivial to an expert) but I think it will really reduce a lot of cognitive load on the part of the reader if these axes are simply \alpha and \beta.

I found it very hard to understand the results of Figure 4 and its associated main text on pgs 8-9, mainly because I’m not sure that I understand what is the second order-coefficient in st. As that will take too long to explain, I think the most efficient way to make this section more accessible is to address the following questions: What is the significance of this result; why does it matter? Can you provide an intuition/feeling for the reader?

It took me quite a while to understand the difference between panels A & B in Figure 5. This can be easily be fixed by overlaying a rectangle on panel B corresponding to the range of panel A (or you can make panel A an inset of B, but that sounds like a lot of work and might take up a lot of space).

Version 1:

Reviewer comments:

Reviewer #1

(Remarks to the Author)
The authors have revised their manuscript comprehensively and with love to detail. I warmly recommend publication in present form.

(Remarks on code availability)

Reviewer #2

(Remarks to the Author)
The paper is significantly improved by the rewrite.
The set of results was already very interesting.
Moreover, many interesting cases were added (deleterious mutations, variable D).
The flow is better, and everything is clearer.

(Remarks on code availability)

Reviewer #3

(Remarks to the Author)
I acted in the capacity of reviewer 3 in the first round. I am happy with the changes that the authors made to the manuscript in addressing all my comments from the first round. I find the readability of the manuscript is very much improved, especially with the reorganization and highlighting of novel results as suggested by reviewer 2.

As such, my remaining comments are incredibly superficial and I entirely endorse the publication of the manuscript.

1. In Figure 5 (and S13), I believe from Convention 1 of SI Section 7 that there are only 2 discrete values for the migration probability m (m_F and m_B)? If this is true, then these 2 discrete values should be shown clearly by using a discrete scale for m in order to better highlight for the reader the model conventions that were applied. If this is not true, then feel free to disregard and keep the scale bar as it is.

2. Now that the results for generic graph structures are presented just prior to the results for low migration rate, it makes me wonder: Would it be reasonable to expect that generic graph structures with a smaller degree than the line and star would have a stronger refugia effect under the low migration regime? I'm *not* asking for any further analysis or simulations! It's just that the narrative flow of the paper now makes me wonder if I understood the results correctly.

(Remarks on code availability)

Open Access This Peer Review File is licensed under a Creative Commons Attribution 4.0 International License, which permits use, sharing, adaptation, distribution and reproduction in any medium or format, as long as you give appropriate credit to the original author(s) and the source, provide a link to the Creative Commons license, and indicate if changes were made.
In cases where reviewers are anonymous, credit should be given to 'Anonymous Referee' and the source.
The images or other third party material in this Peer Review File are included in the article's Creative Commons license, unless indicated otherwise in a credit line to the material. If material is not included in the article's Creative Commons license and your intended use is not permitted by statutory regulation or exceeds the permitted use, you will need to obtain permission directly from the copyright holder.
To view a copy of this license, visit https://creativecommons.org/licenses/by/4.0/

Reviewer #1

This study examines how environmental heterogeneity influences the fixation of mutants in spatially structured populations, where subpopulations (demes) are located on the nodes of a graph. The research explores how differences in local environments interact with migration between demes to shape evolutionary outcomes, particularly the probability and speed of mutant fixation.

When migration between demes is frequent, environmental heterogeneity is shown to amplify natural selection. Under these conditions, both mutant fixation and extinction occur more rapidly, promoting the swift establishment of beneficial mutants. This effect is demonstrated in specific network structures such as the star graph, even more strongly in the line graph, and also in a broader class of graphs. Amplification arises when mutants possess a greater fitness advantage in demes characterized by stronger migration outflow. In circulation graphs, where migration inflow and outflow are balanced at every deme, environmental heterogeneity exerts no influence at first order but increases the fixation probability of beneficial mutants at second order.

When migration between demes is infrequent, environmental heterogeneity can also enhance the amplification of selection. In this regime, demes that provide a sufficient fitness advantage to

mutants may serve as refuges, protecting mutant lineages and facilitating their eventual spread throughout the population.

These are interesting and novel results. The manuscript is very comprehensive and well-written. Overall, I have enjoyed reading and I am in favour of publication subject to the consideration of the following comments.

>>> We thank the reviewer for the positive and encouraging feedback, as well as for the nice suggestions, which allowed us to improve our manuscript.

1) Research on the importance of spatially structured populations in evolution has in the past considered significantly more realistic interaction networks. In this regard the work is missing out on the opportunity to consider more complex networks, beyond the star graph, the line graph, and the currently considered artificial graphs. While I appreciate that these interaction graphs are to a degree still analytically traceable, simulation procedures would allow the consideration of more general networks with properties that correspond more closely to real world complex networks. Scale-free networks, small-world networks, and the most recently considered higher-order networks (e.g. Evolutionary dynamics of higher-order interactions in social networks, Nat. Hum. Behav. 5, 586-595 (2021)) come to mind. The authors should consider this extension to their current research, at least in discussion and future work, if actual results are for some reason unattainable.

>>> We thank the reviewer for raising this important point. To address it, we have substantially extended our analysis and revised the manuscript accordingly.

First, we added a new section in the Results titled “Amplification exists across graph structures with strong migration outflow from demes where mutants are advantaged”, where we systematically analyze all connected graphs with five demes. Under a convention ensuring stronger migration outflow from demes where mutants are advantaged, we show that amplification of selection exists in all of these graphs, except the cycle and the clique, which are circulations. As shown in the manuscript, circulations have the same fixation probability as well-mixed populations to first order in the effective mutant selective advantage. These results, illustrated in the new Figure 5, demonstrate that the amplification mechanism identified in our work is not restricted to specific analytically tractable graphs (such as the star or the line), but extends to a diverse set of graph structures. We provide additional details in the new SI Section 7, where we also analyze these graphs under two alternative conventions for migration and environment heterogeneity. In these cases, we recover suppression of selection, suggesting that suppression remains the most generic effect of spatial structure under frequent migrations in the branching process regime, consistent with previous results for homogeneous environments (see Ref. 36). Amplification therefore requires specific conditions, such as the overall migration flow going in the same direction as the environment gradient.

Second, we expanded the Discussion to explicitly address extensions to larger and more complex graphs. We now discuss the perspectives of studying scale-free and small-world networks. We also mention possible generalizations to group interactions and higher-order networks, and we cite the interesting reference suggested by the reviewer. The reason we did

not include explicit results for scale-free or small-world networks in the present work is that such properties typically emerge only in large graphs. In this regime, the number of possible configurations of migration probabilities and environment heterogeneities grows rapidly, making a systematic exploration challenging and beyond the scope of the current study. We believe that extending our framework to these more complex settings is a promising direction for future work.

2) When referring to the importance of models on graphs and evolutionary graph theory, and in particular that specific graphs, under specific update rules defining the dynamics, can amplify or suppress natural selection, I would recommend the reviews Evolutionary dynamics of group interactions on structured populations: A review, J. R. Soc. Interface 10, 20120997 (2013) and Coevolutionary games - A mini review, BioSystems 99, 109-125 (2010), the later also when authors mention time-varying environments as promising future research avenues.

>>> We thank the reviewer for bringing these interesting references to our attention. Our manuscript is centered on mutants whose fitness depends on the deme but not on interactions between individuals within a deme or on deme composition. However, social interactions, evolutionary games, and the evolution of cooperation are situations where it would be of clear interest to study the impact of environment heterogeneity in spatially structured populations. We added a new paragraph at the end of the Discussion to address this interesting perspective, and we cited the references suggested by the reviewer there.

3) The abstract open prominently with the fact that complex spatial structure and environment heterogeneity shape the evolution of natural populations, but the work then does make clear which are these natural populations the authors have in mind and for which this research would be most applicable to. The applications and implications should be spelled out more explicitly, and I would expect here also a delineation whether this research applies just to biological system or also to social systems.

>>> We thank the reviewer for raising this important point. To address it, we have revised and expanded the end of the Discussion (last three paragraphs). There, we discuss the relevance of our work to experimental studies. Furthermore, we mention concrete natural populations where the results have relevant implications, including the gut microbiota and bacterial populations subjected to antibiotic gradients. Since our study is centered on mutants whose fitness depends on the deme but not on interactions between individuals within a deme or on deme composition, the applications we have in mind are primarily biological populations, as opposed to social systems. However, we agree that the latter are a very interesting perspective for future work. We now explicitly discuss the interesting perspective of extending our work to social interactions in the last paragraph of the Discussion.

If a revision is granted, I will be happy to review a revised manuscript.

Reviewer #2

Summary:

The environmental heterogeneity arises in many real-world situations and has a subtle interplay with population structure.
The authors extend their previous work to consider the effects of environmental heterogeneity in structured populations.
They adapt the Wright-Fisher-like model to describe the heterogeneity.
Multiple demes with capacity K are connected in a migration graph.
First, all demes experience exponential growth for time t, where the mutants have deme-dependent fitness \delta_i s for deme i.
Then, to every deme, the new population of size K is sampled from the deme itself and all neighboring demes given by migration probabilities.
The process repeats until complete fixation or extinction of the mutants.

The model is robust and captures many real-world scenarios.
This work is an initial intensive exploration of the model.
The results are not surprising, but they explain the most important scenarios.

In general, it is good work that requires some rewriting.

One important result is for circulations, where the effects of the first order are always the same (proportional to st). The heterogeneity can increase the effects of the second order.

In general, heterogeneous graphs are amplifiers if demes with higher outflow than inflow have a higher than average mutant advantage.

These effects are examined on the star (with simulations).

On the line, there is an elegant formula for the amplification of mutants.
(It is the effect of consecutive biases in the outflows and inflows)

Finally, the rare migration regime is examined where the deme first homogenizes, and then it spreads.

>>> We thank the reviewer for the positive and encouraging comments, as well as for the thorough reading and for the nice suggestions, which allowed us to improve our manuscript, in particular regarding its organization. While some results are intuitive, the simultaneous amplification of selection and acceleration of fixation and extinction is unexpected in the context of evolutionary graph theory. Indeed, in previous studies, amplifiers of selection generally slow down fixation (see Refs. [26–29, 69]), so our results highlight a novel and nontrivial effect of environment heterogeneity on structured populations.

Major Comments:

The model is described in three places: the first paragraph of Results, Models and Methods, and SI, section 1.1.
The results are not understandable without the model described in SI.
The work would benefit from describing the model directly in the Results section.
(It should definitely include Figure S1, at least panel A. I like panel B too.)
To understand any result, knowledge of the model is vital.

>>> We thank the reviewer for pointing out the need to clarify the model in the main text, especially early on, and to better delineate the separation between different sections. We have made substantial changes to address this point, as follows.

First, we expanded the first subsection of the Results, titled "Including environment heterogeneity in deme-structured populations on graphs", to include all the key model points necessary to understand the rest of the Results. In particular, we explicitly define the baseline effective fitness advantage, st, used throughout the manuscript. In addition, we define the elementary steps of the serial dilution model, illustrate them in Fig 1 (previously Fig S1A), following the reviewer's suggestion, and delineate the connection of this model to structured Wright-Fisher models.

Second, we reorganized the Supplementary Information to clarify that it contains analytical derivations rather than a definition of the model. In particular, the former SI section titled "Model" has been renamed and reorganized to focus on analytical derivations (branching-process analysis and Poisson approximation). The opening of this section now explicitly states that the stochastic process is defined in the main text, and is only briefly restated in the SI to fix notation for the calculations, ensuring that the Results are fully interpretable from the main text alone, with the SI providing technical derivations.

The order of the presentation of results in the main text is not good.
The graph that achieves the biggest change in fixation probability (probably deemed the most interesting result) is stated first.
However, there are three sources of heterogeneity: (i) the mutant fitness heterogeneity, (ii) the migration inflow and outflow heterogeneity, and (iii) the graph heterogeneity.
I suggest changing the order of presentation such that points (ii) and (iii) are added first separately and then combined.
(This means the first should be either circulations or complete graphs with strong outflow deme)

>>> We thank the reviewer for providing helpful guidance on logical flow. We initially presented the star and the line first because they are broadly studied, analytically tractable, and display non-trivial effects on mutant fixation probability. Furthermore, we found novel impacts of environment heterogeneity there, which hold to first order and are therefore quantitatively important. That said, we agree that starting with simpler cases and baselines, and gradually adding complexity, facilitates logical flow. We have done substantial reordering and rewriting to address this point, as follows.

We added a new paragraph at the end of the first subsection of the Results (titled "Including environment heterogeneity in deme-structured populations on graphs"). In this

paragraph, we briefly recall how spatial structure impacts mutant fixation in deme-structured populations on graphs with homogeneous environments (i.e. (ii) and (iii) without (i)).

The next subsection of the Results is now devoted to circulations with heterogeneous environments. This subsection thus corresponds to incorporating (i) in the simplest category of spatial structures. We moved this section to the beginning of the Results; previously, circulations were described later. Since we show that the circulation theorem holds to first order, circulation graphs, in particular the clique, then serve as a baseline that is independent of environment heterogeneity to first order.

Then, we have retained the initial order for more complex structures, i.e. the star, the line and the fully-connected graph with a special deme. While starting from the latter would in principle be possible, we feel that starting with well-known structures in the field, such as the star and the line, enhances readability and accessibility. Furthermore, we view the fully-connected graph with a special deme as a first generalization of our results. To clarify the logic, we now explain that we identified a mechanism for amplification in the cases of the star and the line, and that we then investigate whether these findings hold beyond these structures. Specifically, we investigate what happens when migration asymmetries arise solely from differences in migration probabilities, rather than from the graph connection pattern.

Finally, we consider further generalizations, including in particular a new section titled "Amplification exists across graph structures with strong migration outflow from demes where mutants are advantaged", where we systematically analyze all connected graphs with five demes. Under a convention ensuring stronger migration outflow from demes where mutants are advantaged, we show that amplification of selection exists in all of these graphs, except the cycle and the clique, which are circulations. These results, illustrated in the new Figure 5, demonstrate that the amplification mechanism is not restricted to analytically tractable graphs (such as the star or the line), but extends to a diverse set of graph structures. This addresses point (iii) more fully than in the first version of our manuscript.

In addition to these modifications of the main text, we reorganized the Supplementary Information to use a matching order of sections.

There should also be a paragraph (before results) that considers baselines that the results are compared against.
This would unify the results.

>>> As mentioned in the previous response, we have done substantial reordering and rewriting to improve logical flow. This includes starting with two baseline categories for comparison.

In the new paragraph at the end of the first subsection of the Results (titled "Including environment heterogeneity in deme-structured populations on graphs"), we briefly recall how spatial structure impacts mutant fixation in deme-structured populations on graphs with homogeneous environments.

In the next subsection of the Results, we now consider circulations. Because the circulation theorem holds to first order, circulation graphs, in particular the clique, serve as a baseline that is independent of environment heterogeneity.

Minor Comments:

I would like to see the explanations of the observed and proven effects.
Something like: Inhabitants of a deme with strong outflow are more likely to survive, which means that any increase in fitness in this deme helps mutants more than in other demes.

>>> We agree that this intuitive explanation is useful, and have added it to the Discussion: "Qualitatively, individuals in a deme with strong outflow contribute more to next generations. Thus, a fitness increase in this deme helps mutants more than in other demes, contributing to amplification of selection."

I would like to see how the effects change for large D (the size of the deme).
The only figure that varies the constant D is S3.

>>> We thank the reviewer for this insightful suggestion, which prompted us to investigate the impact of the number D of demes on our results. It is now explicitly addressed in the second paragraph of the new Results subsection titled "Amplification of selection extends to deleterious mutants and large graphs". Details of this new analysis are provided in the new SI Section 6. Briefly, we vary D for the star, the line, and the highly symmetric fully connected structure with one special deme. We show that amplification of selection can persist in large graphs with heterogeneous environments, when overall migration flows from demes with the strongest mutant fitness advantage to those with weaker advantage. These results demonstrate the generality of our findings, while also showing that specific conditions on migration and environment are required to maintain significant amplification in large graphs.

Some writing is not very inviting, for instance, in SI, section 1.1, end of the third paragraph, the index i appears for m_{ij} and m_{ki} in one sentence.

>>> We changed the indices to ensure that they are consistent in that sentence.

For the high migration regime, \delta_i \ge 0, what would \delta_i < 0 change in computations?

>>> We thank the reviewer for the interesting question.

Our branching process calculation in the frequent migration regime requires that mutants are beneficial, i.e. delta_i st > 0. Starting from Eq. 6, the branching process predicts certain extinction when st < 0 or st = 0. Hence, our analytical approach cannot be directly extended to the case where st < 0 or delta_i < 0.

To nevertheless explore deleterious mutants, we performed simulations with s<0. This analysis is summarized in the first paragraph of the new subsection of Results titled "Amplification of selection extends to deleterious mutants and large graphs". Details are presented in the new SI Section 5. Our simulations indicate that amplification extends to deleterious mutants in all cases considered.

Reviewer #3

Fruet et al., use evolutionary graph theory to understand how different spatial structures impact the fixation probability of beneficial mutations (s) whose advantage over the wild-type differ between demes (d_i), herein called “environment heterogeneity”. In a couple of special spatial structures as well as in a generalized spatial framework, they show that environment heterogeneity can lead to amplifier demes with an increased fixation probability and decreased fixation time; this is in contrast to previous models of homogeneous environments that found amplifier (or suppressor) demes had a positive relationship between fixation probability and fixation time. They identify the conditions when amplification happens (\beta < \alpha < 1 or 1 < \alpha < \beta) and show that these boundaries hold even without strong migrational symmetry. The intuition is that amplifiers demes are ones where mutants are highly beneficial and act either as outflows in the strong migration regime or refugia in the weak regime.

I liked that the authors were able to arrive at results that hold for arbitrary spatial structures and migrational symmetries by starting from a couple of special spatial structures (star and line) and strong migrational asymmetries. The manuscript is motivated by natural microbial populations and, in addition to its mathematical rigour, is understandable to a general audience in theoretical evolution or bacterial population growth.

>>> We thank the reviewer for the positive and constructive feedback, as well as for the helpful suggestions, which allowed us to improve our manuscript, in particular regarding connections to the broader context and literature.

My main complaint with the manuscript is that it does not provide a clear definition of how environment heterogeneity is conceptualized and implemented straight from the Introduction. Evolutionary readers who are already familiar with the rich literature on environmental heterogeneity, environmental gradients, and gene flow (e.g., Kirkpatrick & Barton 1997 DOI: 10.1086/286054, Lenormand 2002 DOI: 10.1016/S0169-5347(02)02497-7) will be primed to assume that environmental heterogeneity refers to antagonistic selective effects between environments. But this is not the case! For Fruet et al., the mutant is always beneficial even though the strength of the beneficial effect is variable between demes. This is an important novelty of the work and should already be made clear from the Introduction, probably somewhere in the second last paragraph. In addition, it would be nice if the Discussion can verbally contrast the current results with those from population genetics (e.g., the classic works listed above, or newer) or at least address the vast theoretical evolutionary literature on environmental heterogeneity that lies outside of evolutionary graph theory. In particular, I wonder how amplification (or suppression), from the field of evolutionary graph theory, relates to swamping, from the field of population genetics (i.e., if they are/can be related; or otherwise to explain that these concepts are entirely unrelated).

>>> We thank the reviewer for the helpful suggestion, which allowed us to better contextualize our findings within the literature. We did the following modifications to address this point.

First, we amended the second to last paragraph of the Introduction, as suggested by the reviewer, and clarified that we considered beneficial mutants, in contrast with many previous studies. Specifically, we added the following sentence: “In population genetics, environment heterogeneity has been studied in continuous spatially extended populations, but in the context of species range limits and local adaptation rather than mutant fixation in the whole population [37–43]. Importantly, these studies typically consider antagonistic effects of selection across space, while here, our main focus is on mutants that are beneficial throughout the structure, with the strength of their beneficial effect varying across demes.”

Second, in the Discussion, we now discuss the relationship between our findings and swamping in population genetics. At the end of the fourth paragraph, we added: “In population genetics, environment heterogeneity in spatially extended populations has also been studied in the context of species range limits. In these works, when a mutation is beneficial in one region but deleterious in another, gene flow from the region where the mutation is deleterious can hinder local adaptation in the one where it is beneficial, an effect known as swamping [37–43]. These studies typically consider continuous spatial structures and antagonistic effects of selection across space [38, 40, 41] or quantitative traits whose optimum varies spatially [39, 42], and focus on range expansion rather than mutant fixation or extinction. Despite these important differences, it is interesting to note that in swamping, migration from regions where a mutant is disadvantaged to regions where it is advantaged hampers adaptation, echoing our finding that the opposite migration flow (from more favorable to less favorable regions) can amplify natural selection.”

My other comments are relatively minor:

On the bottom of pg 3 the “first order-coefficient (slope) in st” is introduced. This is referred to in the caption of Figure 1 as the “first order-coefficient (slope)” but later on as “first order in st” or simply “first order”. I found the coefficient difficult to understand, and the inconsistent terminology throughout the manuscript made it even more confusing. Consistent phrasing would greatly improve clarity.

>>> We apologize for being slightly cryptic. To clarify this important point, we did the following modifications:

- We removed all instances of “slope” and now consistently refer to the “first-order coefficient in the expansion of the mutant fixation probability in the baseline effective mutant fitness advantage st”.
- We added Eq. 1 at the beginning of the Results to explicitly define what we mean by the first-order and second-order coefficients in the expansion of the fixation probability in st. Later references point to that equation, and mention that the first-order coefficient corresponds to a in that equation, thus improving clarity for the reader.

I really like the schematics of the spatial models that are included throughout the text, they are incredibly helpful! But it took me a really long time to understand what is \sigma_C in Figure 1A because this value is not clearly defined in the caption and because the schematic focuses the reader’s attention to the migration rates. Maybe find a way to label \sigma_C in the schematic of

Figure 1A so that the reader can very quickly understand the whole plot. (It might also be helpful to make the \alpha on the schematic more visible as \alpha is important for panel B and later).

>>> We clarified this figure (now Figure 2), following the reviewer's suggestions.

- We labeled the schematic as Figure 2A.
- We added a histogram below the schematic to visually define sigma_C.
- We spelled out the formal definition of sigma_C in the figure caption.
- We spelled out the definition of alpha just below the title of Figure 2A to make it more visible for later reference.

Figure 2. I got confused that the schematics on the right side are referred to in the main text before panel A is referred to. This is especially confusing as these schematics do not have a panel label. I would suggest moving them to the left side and **labeling them** panel A (that way they can be directly referred to in the main text, middle of pg.4).

>>> We clarified this figure (now Figure 3), following the reviewer's suggestions.

- We reordered panels so that the schematics appear first, at the top left of the figure.
- We labeled the schematics as Figure 3A, allowing direct reference in the main text.

For consistency, we also labeled the schematics on Figure 4.

To help the population geneticists in the audience, note that the "clique" spatial structure was first called the "island model" by Sewall Wright and the "line" called the "stepping stone" by Motoo Kimura.

>>> We thank the reviewer for this useful remark. We have added references to the island model and the linear and circular stepping-stone models, noting that they correspond, respectively, to the clique, the line, and the cycle. We also cite the original works by Sewall Wright and Motoo Kimura, as well as studies by Takeo Maruyama that distinguish between linear and circular stepping-stone models.

Figure 3D took me a long time to figure out because the inverse \alpha and inverse \beta values are being used. I know it will be rather annoying to fix this (and seems trivial to an expert) but I think it will really reduce a lot of cognitive load on the part of the reader if these axes are simply \alpha and \beta.

>>> To improve clarity, we redefined migration asymmetry \tilde{\alpha}=m1/m2 and environment heterogeneity beta=delta1/delta2 in the fully-connected structure with a special deme. Note that these definition choices are arbitrary and do not impact the results (in the sense that one can call migration asymmetry either m1/m2 or m2/m1). This new choice allowed us to simplify Figure 4E (previously Figure 3D), so that its axes now directly show \tilde{\alpha} and beta without involving inverses. Because the mapping to the star structure is more direct when using m2/m1, we renamed migration asymmetry \tilde{\alpha} instead of alpha. The mapping is explained in the Supplementary Information (Section 4.1.1).

I found it very hard to understand the results of Figure 4 and its associated main text on pgs 8-9, mainly because I'm not sure that I understand what is the second order-coefficient in st. As that will take too long to explain, I think the most efficient way to make this section more accessible is to address the following questions: What is the significance of this result; why does it matter? Can you provide an intuition/feeling for the reader?

>>> We thank the reviewer for raising these points. We apologize for being slightly cryptic regarding the interpretation and significance of the second-order term for circulations.

To clarify the definition of the second order coefficient, we added Eq. 1 at the beginning of the Results. Later references point to that equation, and mention that the second-order coefficient corresponds to -b in that equation.

That said, this second order effect on fixation probabilities is quantitatively small, and heterogeneous circulations essentially follow the circulation theorem, which holds to first order, and therefore provides a natural baseline for assessing the effects of heterogeneity in non-circulation graphs. We now clarify these important points at the end of the Results subsection titled “Environment heterogeneity in circulation graphs: a baseline”: “These findings demonstrate that, beyond first order, environment heterogeneity has non-trivial theoretical effects even in circulation graphs, leading to a breakdown of the circulation theorem to second order. However, this effect remains quantitatively small, as scales as (st)^2. This motivates turning to non-circulation graphs to investigate whether environment heterogeneity has stronger effects. Importantly, circulation graphs, and in particular the clique, will serve as a baseline independent of environment heterogeneity to first order in st.”

Our main results are the amplification of selection and the acceleration of fixation and extinction that are observed in the star and the line, and the fact that amplification can be generic when mutants have a stronger fitness advantage upstream of the overall migration flow. All these results hold to first order in st, and are thus quantitatively important. To focus the main narrative around first-order, quantitatively dominant effects, we decided to shorten the paragraph on these second-order effects, and to move Figure 4 to the Supplementary Information (it is now Figure S3).

It took me quite a while to understand the difference between panels A & B in Figure 5. This can be easily be fixed by overlaying a rectangle on panel B corresponding to the range of panel A (or you can make panel A an inset of B, but that sounds like a lot of work and might take up a lot of space).

>>> In Figure 6 (previously Figure 5), we added a rectangle on panel B corresponding to the range of panel A, following the reviewer's suggestion.

Reviewer #1 (Remarks to the Author):

The authors have revised their manuscript comprehensively and with love to detail. I warmly recommend publication in present form.

>>> We thank the reviewer for their support and for their helpful suggestions in the previous round.

Reviewer #2 (Remarks to the Author):

The paper is significantly improved by the rewrite.
The set of results was already very interesting.
Moreover, many interesting cases were added (deleterious mutations, variable D).
The flow is better, and everything is clearer.

>>> We thank the reviewer for the positive feedback, and for their constructive recommendations in the previous round, which helped improve the manuscript.

Reviewer #3 (Remarks to the Author):

I acted in the capacity of reviewer 3 in the first round. I am happy with the changes that the authors made to the manuscript in addressing all my comments from the first round. I find the readability of the manuscript is very much improved, especially with the reorganization and highlighting of novel results as suggested by reviewer 2.

As such, my remaining comments are incredibly superficial and I entirely endorse the publication of the manuscript.

>>> We thank the reviewer for their positive comments, and for their useful recommendations, both in the previous round and in this revision, which strengthened the manuscript.

1. In Figure 5 (and S13), I believe from Convention 1 of SI Section 7 that there are only 2 discrete values for the migration probability m (m_F and m_B)? If this is true, then these 2 discrete values should be shown clearly by using a discrete scale for m in order to better highlight for the reader the model conventions that were applied. If this is not true, then feel free to disregard and keep the scale bar as it is.

>>> The reviewer is correct: in Convention 1, there are only two discrete values (m_F and m_B) for migration probabilities between demes. However, the probability to stay in a given deme i can take other values, as it is determined by $m_{ii} = 1 - \Sigma_{j\neq i}\, m_{ji}$, given the convention that, for all i, $\Sigma_j\, m_{ji} = 1$. We chose a continuous scale bar to also capture these values. To clarify this point, we have now indicated the important values m_F and m_B in our continuous scale bar for migration

probabilities, both in Figure 5 and in Figure S13. We also corrected a typo in the tick labels of this scale bar. We thank the reviewer for this suggestion, which allowed us to clarify our figures.

2. Now that the results for generic graph structures are presented just prior to the results for low migration rate, it makes me wonder: Would it be reasonable to expect that generic graph structures with a smaller degree than the line and star would have a stronger refugia effect under the low migration regime? I'm *not* asking for any further analysis or simulations! It's just that the narrative flow of the paper now makes me wonder if I understood the results correctly.

>>> We thank the reviewer for this interesting question. The mechanism of amplification associated with environmental heterogeneity differs between the rare migration regime and the frequent migration regime. While it would be interesting to study generic graphs in the rare migration regime, analytical expressions are challenging to obtain in these cases, which is why we focused on the star and the line. Our analysis demonstrates that, in the rare migration regime, the key ingredient is that (at least) one deme acts as a refugia. The comparison between the star and the line suggests that the effect is stronger when this special deme has a higher degree, in contrast with the frequent migration regime. A more general study of spatial structures with heterogeneous environments under rare migrations would be an interesting direction for future work.
